# A mononucleotide repeat in PRRT2 is an important, frequent target of mismatch repair deficiency in cancer

**DOI:** 10.18632/oncotarget.13464

**Published:** 2016-11-19

**Authors:** Inês Teles Alves, David Cano, René Böttcher, Hetty van der Korput, Winand Dinjens, Guido Jenster, Jan Trapman

**Affiliations:** ^1^ Department of Urology, Erasmus MC, Rotterdam, The Netherlands; ^2^ Department of Pathology, Erasmus MC, Rotterdam, The Netherlands

**Keywords:** PRRT2, mismatch repair, mononucleotide repeat, prostate cancer, colorectal cancer

## Abstract

The DNA mismatch repair (MMR) system corrects DNA replication mismatches thereby contributing to the maintenance of genomic stability. MMR deficiency has been observed in prostate cancer but its impact on the genomic landscape of these tumours is not known. In order to identify MMR associated mutations in prostate cancer we have performed whole genome sequencing of the MMR deficient PC346C prostate cancer cell line. We detected a total of 1196 mutations in PC346C which was 1.5-fold higher compared to a MMR proficient prostate cancer sample (G089). Of all different mutation classes, frameshifts in mononucleotide repeat (MNR) sequences were significantly enriched in the PC346C sample. As a result, a selection of genes with frameshift mutations in MNR was further assessed regarding its mutational status in a comprehensive panel of prostate, ovarian, endometrial and colorectal cancer cell lines. We identified *PRRT2* and *DAB2IP* to be frequently mutated in MMR deficient cell lines, colorectal and endometrial cancer patient samples. Further characterization of *PRRT2* revealed an important role of this gene in cancer biology. Both normal prostate cell lines and a colorectal cancer cell line showed increased proliferation, migration and invasion when expressing the mutated form of PRRT2 (ΔPRRT2). The wild-type PRRT2 (PRRT2^wt^) had an inhibitory effect in proliferation, consistent with the low expression level of *PRRT2* in cancer versus normal prostate samples.

## INTRODUCTION

DNA mismatch repair (MMR) is a highly conserved repair mechanism responsible for the correction of mismatched base pairs occurring during the DNA replication process [1]. Defects in this repair mechanism are associated with mutations in microsatellite repeat sequences [2], which consist of tandemly repeated motifs of one (mono) to six (hexa) nucleotides [3]. The mismatch repair heterodimers MSH2/MSH6 and MSH2/MSH3 detect replication errors and recruit the MLH1/PMS2 complex which in turn degrades and resynthesizes the mutated stretch [4]. When MMR is defective, these errors will not be corrected and will lead to frequent microsatellite instability (MSI) [2].

Most microsatellite sequences are located in non-coding DNA with limited consequences to cell functions [5]. But recently, defects in MMR were shown to originate a mutator phenotype with an increased rate of frameshift mutations in genes including those with coding microsatellites [6]. Affected genes are involved in signal transduction pathways (*TGFβRII, IGFIIR* and *PTEN*) [7–9], apoptosis (*BAX* and *CASP5*) [10, 11], DNA repair (*MBD4*) [12], and transcriptional regulation (*TCF-4, EPHB2, AXIN2*) [13–15]. The proteins resulting from these frameshift mutations can contribute to tumour progression by functional inactivation, a dominant negative effect, or a gain of function [16–18].

Germline mutations in MMR genes are present in over 90% of Lynch syndrome patients, which are characterized by MSI and an increased risk of colorectal cancer and several other cancer types [19]. The MSI phenotype is also observed in approximately 15% of sporadic colorectal cancers [20], 12% of ovarian cancers [21], 20% of endometrial cancers [22, 23], 30% of gastric cancers [24] and less frequent in other cancer types [25]. MMR deficiency is also frequent in *in vitro* growing prostate cancer cell lines. LNCaP cells do not express MSH2 due to gene deletion and *MLH1* is mutated in DU145, resulting in expression of an unstable truncated MLH1 protein [26, 27]. The frequency of MSI reported in prostate cancer patients varies considerably between different studies but it is conclusively lower than in other sporadic cancer types [28, 29].

So far, no whole genome sequencing study has focused on identifying novel genes commonly targeted by MMR deficiency in prostate cancer. Whole genome sequencing of two prostate cancer samples, one with a functional MMR system (G089, a late stage prostate cancer patient) and other with deficiency of the MMR system (PC346C, a prostate cancer cell line with point mutations in both alleles of the MSH2 gene) [30] identified numerous novel gene mutations. Mutations in mononucleotide repeats (MNR) were most specific for the PC346C sample. A set of 17 candidate genes with mutations in MNR was defined and further evaluated in a larger panel of prostate, colorectal, endometrial and ovarian cancer cell lines. We identified proline-rich transmembrane protein 2 (*PRRT2*) and DAB2 interacting protein (*DAB2IP*) to be frequently mutated in all different cancer cell line types. Further analysis showed that both genes were also frequently mutated in colorectal and endometrial cancer patient samples. Functional studies revealed PRRT2 to be implicated in cellular proliferation and migration with the truncated MSI-derived PRRT2 form promoting both processes.

## RESULTS

### Overview of microsatellite mutations in gene sequences

**Figure 1:**
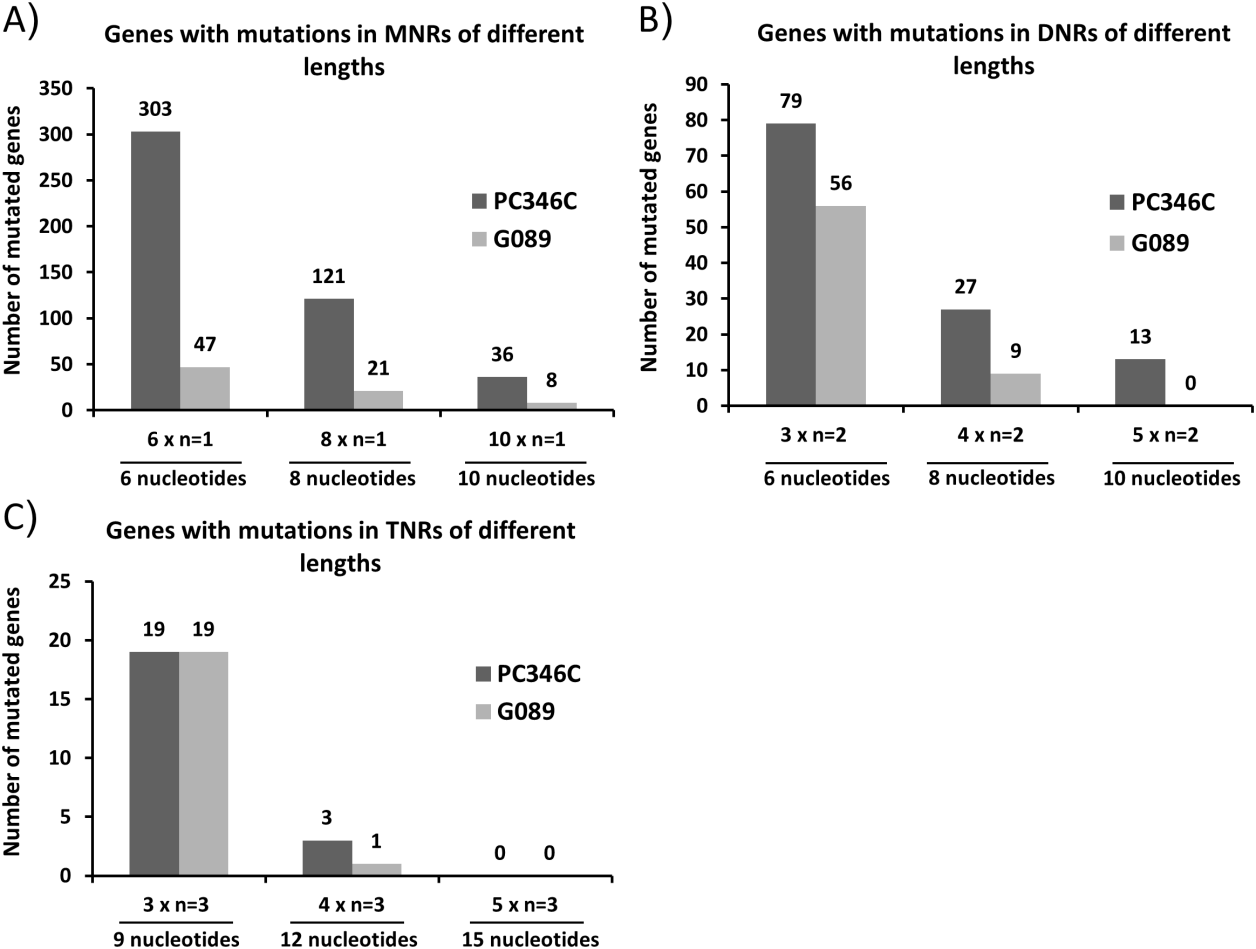
Overview of the mutation spectrum in G089 and PC346C genomic DNA Genes with mutations in mononucleotide repeats (MNRs) **A**., dinucleotide repeats (DNRs) **B**. and trinucleotide repeats (TNRs) **C**. were distributed according to the length of the mutated repeat sequence. The repeat unit length for MNRs is one nucleotide (n=1), for DNRs it is two nucleotides (n=2) and for TNRs it is three nucleotides (n=3). The x-axis shows the number of repeat units: 6, 8 and 10 in case of MNRs; 3x, 4x and 5x in case of DNRs and TNRs.

To identify novel gene mutations caused by MMR deficiency in prostate cancer, we analyzed the whole genome sequence of the MMR-deficient prostate cancer cell line PC346C and the MMR-proficient prostate cancer patient sample (G089). We selected for mutations that would likely disrupt the normal protein function (insertion/deletion introducing frameshifts, and mutations in the start or stop codon). The sequence flanking each gene mutation (10 nucleotides up and downstream) was retrieved using the UCSC genome browser. We selected for mutations occurring in microsatellite sequences in the PC346C sample and absent in G089, thereby enriching for mutations that are caused by MMR deficiency. First, the type and length of repeats were used to attain an overview of the mutation spectrum in both prostate cancer samples (Figures 1A, and 1C). We included in our studies repeats with three or more repeat units, depending on the repeat composition (mono-, di- or tri-nucleotide repeat). Overall, we detected 1196 gene mutations in PC346C and 760 gene mutations in G089 (Supplementary Tables 1 and 2). From the 1196 gene mutations in PC346C, 303 were in mononucleotide repeats (MNRs), 79 in dinucleotide repeats (DNRs) and 19 intrinucleotide repeats (TNRs) (6x n_1_ for MNR, 3x n_2_ for DNR and 3 x n_3_ for TNR) (Supplementary Figure 1A). In G089, 47 gene mutations were in MNRs, 56 in DNRs and 19 in TNRs (6x n_1_ for MNR, 3x n_2_ for DNR and 3 x n_3_ for TNR) (Supplementary Figure 1B).

**Table 1:** List of microsatellite instability target genes in PC346C

Gene	Name	Length of repeat	Mutation in PC346C
CNOT1	CCR4-NOT transcription complex, sub 1	A13	del AA
DAB2IP	DAB2 interacting protein	C8	del C
PRRT2	proline-rich transmembrane protein 2	C9	ins CC
TTC3	tetratricopeptide repeat domain 3	A8	del A
CEP164	centrosomal protein 164kDa	A11	del A
PHACTR4	phosphatase and actin regulator 4	A10	del A
TROVE2	TROVE domain family, member 2	A9	del A
ZFR	zinc finger RNA binding protein	T9	del T
MLL3	myeloid/lymphoid or mixed-lineage leukemia 3	T9	del T
ANLN	anillin, actin binding protein	A7	del A
ANUBL1	AN1, ubiquitin-like, homolog	A7	del A
EPRS	glutamyl-prolyl-tRNA synthetase	T6	ins T
KCNMA1	potassium large conductance calcium-activated channel, subfam M, α1	T9	del T
PDS5A	PDS5, regulator of cohesion maintenance, homolog A (S. cerevisiae)	A6	del A
SFRS12IP1	SFRS12-interacting protein	T10	ins T
TSHZ2	teashirt zinc finger homeobox 2	C7	del C
USP42	ubiquitin specific peptidase 42	A8	del A

Genes are ordered first by the frequency of mutation in MMR deficient prostate cancer samples and second alphabetically. The 3 MMR associated genes used as a positive control are depicted in red.

**Table 2:** Number of MSI positive (MSI) and negative (MSS) prostate, colorectal, endometrial and ovarian cancer cell lines with mutations in the set of MSI target genes in PC346C

	Prostate Cancer		Colorectal Cancer		Endometrial Cancer		Ovarian Cancer	
	MSS	MSI	MSS	MSI	MSS	MSI	MSS	MSI
CNOT1	0/3	4/4	0/3	9/10	0/3	4/4	0/2	1/3
DAB2IP	0/3	3/4	0/3	6/10	0/3	1/4	0/2	0/3
PRRT2	0/3	3/4	0/3	4/10	0/3	4/4	0/2	2/3
TTC3	0/3	2/4	0/3	5/10	0/3	1/4	0/2	0/3
CEP164	0/3	3/4	0/3	8/10	0/3	3/4	0/2	2/3
PHACTR4	0/3	3/4	0/3	7/10	0/3	3/4	0/2	1/3
TROVE2	0/3	1/4	0/3	5/10	0/3	4/4	0/2	1/3
ZFR	0/3	2/4	0/3	4/10	0/3	3/4	0/2	1/3
MLL3	0/3	2/4	0/3	8/10	0/3	3/4	0/2	1/3
ANLN	0/3	1/4	0/3	0/10	0/3	0/4	0/2	0/3
ANUBL1	0/3	1/4	0/3	2/10	0/3	0/4	0/2	0/3
EPRS	0/3	1/4	0/3	0/10	0/3	0/4	0/2	0/3
KCNMA1	0/3	1/4	0/3	6/10	0/3	1/4	0/2	1/3
PDS5A	0/3	1/4	0/3	0/10	0/3	1/4	0/2	0/3
SFRS12IP1	0/3	1/4	0/3	6/10	0/3	2/4	0/2	1/3
TSHZ2	0/3	1/4	0/3	10/10	0/3	3/4	0/2	3/3
USP42	0/3	1/4	0/3	4/10	0/3	4/4	0/2	0/3

The 3 control genes are depicted in red. Three MSS cell lines were available for prostate, colorectal and endometrial cancer and one MSS cell line for ovarian cancer. The number of MSI cell lines available for prostate, colorectal, endometrial and ovarian cancer were four, ten, four and three respectively.

PC346C had a 1.5-fold increase in the number of mutated genes as compared to G089. The number of mutations found in MNR sequences of 6 nucleotides was 6-fold higher in PC346C. A 6-fold increase in MNR gene mutations in PC346C was also observed for MNRs of length n_8_. Since MNRs showed, compared to di- and tri-nucleotide repeats, a higher incidence of mutations in MSI+ cancers we focused on these repeats.

We separated the mutated genes in PC346C and G089 in A, T, C and G MNRs categories of different lengths (Supplementary Table 3). The UCSC build hg18 was used as reference for gene annotation. The number of C and G MNRs in reference genes was considerable lower than that of A and T repeats. As expected the mutations in C and G MNRs in PC346C and G089 was also much lower than that of A and T MNRs (Supplementary Table 3).

### Novel target genes of microsatellite instability

To specifically select novel gene mutations associated with MMR deficiency with expected functional impact we first filtered for mutations in MNR sequences in PC346C, which were 6 nucleotides or longer. We excluded LOC annotated gene symbols and genes already known to be associated with MMR deficiency or MSI by using the text mining software Anni 2.0. (http://biosemantics.org/index.php/software/anni). We identified over 35 mutated genes in PC346C, which were already known as targets of MSI or are associated with MMR deficiency including *TGFβRII*, *PTEN*, *BAX*, *MLH3*, *MSH6* and *MSH2*. The frequent identification of known MMR-associated genes confirmed the validity of our approach. This procedure left 119 genes for further selection. Next, Ingenuity Pathway Analysis (IPA) was used to identify genes with functions most likely to be cancer-associated, like cell cycle, cellular growth, apoptosis and other important cell signaling pathways. Finally, we checked the expression profile of candidate genes using Affymetrix exon array data of 90 prostate cancer RNA samples and 17 normal controls (Supplementary Materials and Methods). A final subset of 14 novel genes showing MNR mutations and differential expression between normal and prostate cancer samples was assessed for their mutation status in a larger panel of cancer cell lines (Table 1). As positive controls we added three known MSI target genes (*PHACTR4*, *MLL3* and *CEP164*).

### Frequency of microsatellite instability target genes

**Figure 2:**
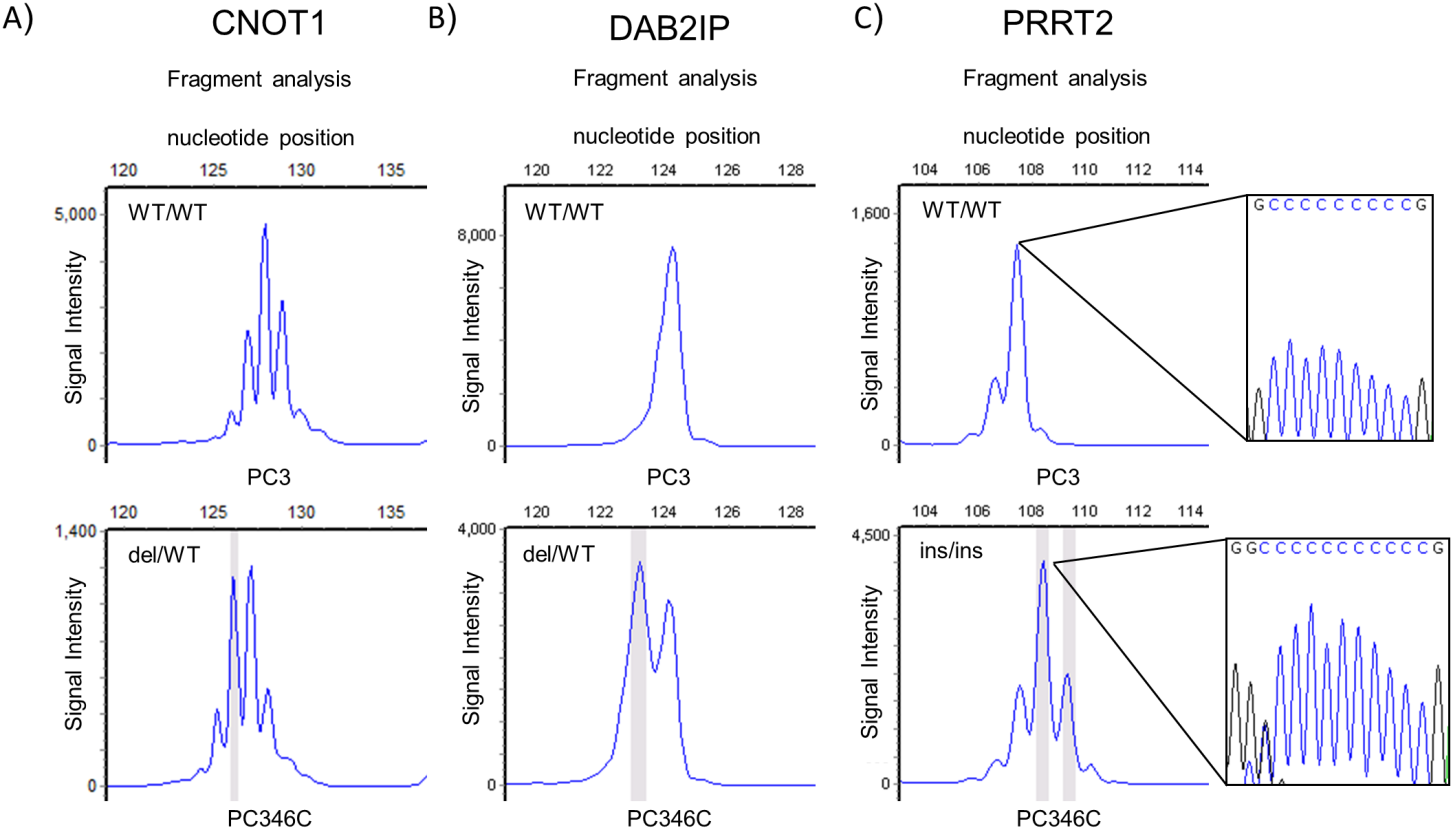
MNR repeat analysis of top mutated genes in prostate cancer in genomic DNA from prostate cancer cell lines PC346C (MMR deficient) and PC3 (MMR proficient) Fragment size analyses of PCR amplified fragments of *CNOT1* **A**., *DAB2IP* **B**. and *PRRT2* **C**. containing the repeat are presented. The highlighted peaks represent a mutant allele. PCR primers are presented in supplementary data. WT: wild type; del: deletion; ins: insertion. Indicated *PRRT2* PCR fragments are flanked by sequence analysis of the repeat.

Microsatellite status of the set of target genes was examined in three MMR-proficient prostate cancer cell lines and in four MMR-deficient prostate cancer cell lines (Figure 2, Supplementary Table 4, Supplementary Figure 2). None of the MMR-proficient prostate cancer cell lines showed MSI in the selected gene panel. Controls *CEP164*, *PHACTR4* and *MLL3* were mutated in two or more of the MMR-deficient cell lines. We found that *CNOT1*, *DAB2IP* and *PRRT2* were mutated in at least three MMR-deficient cell lines (Figure 2, Supplementary Table 4).

Next, we expanded the cell line panel with ten colorectal, four endometrial and three ovarian cancer MMR-deficient cell lines together with control MMR-proficient cell lines of each cancer type. As observed in the MMR-proficient prostate cancer cell lines, colorectal, endometrial and ovarian cancer MMR-proficient cell lines showed no mutations in the analysed genes (Table 2 and Supplementary Table 5). The control genes *CEP164*, *PHACTR4* and *MLL3* displayed as expected high mutation frequencies, ranging from 30% to 80% in the MSI cancer cell lines (Table 2 and Supplementary Table 5). Also here we identified *CNOT1*, *DAB2IP* and *PRRT2* as novel MSI target genes, in the MMR-deficient cancer cell lines, although with varying frequency. The genes displayed mostly deletions and to a lesser extend insertions (Table 2 and Supplementary Table 5). The mutations shifted the open reading frame of the affected genes and consequently predicted to result in synthesis of truncated proteins due to premature termination (Supplementary Table 1). *CNOT1* contains a n_13_ A repeat in the open reading frame located 10 nucleotides upstream of the stop codon. A frameshift mutation at this position might not be critical for the protein and no further research was conducted for this gene. The n_9_ C repeat in *PRRT2* and the n_8_ C repeat in *DAB2IP* displayed a mutation pattern reflecting both insertions and deletions. (Supplementary Figure 3A, 3B).

### Frequency of *PRRT2* and *DA2IP* repeat mutations in MSI patient cancer samples

Next, we checked whether the mutation frequencies of *PRRT2* and *DAB2IP* observed in the cancer cell line panels could be confirmed in primary cancer samples. We tested a total of 80 prostate cancer patient samples, including late stage and metastasised cancers. None of the samples displayed the mutations in either of the genes (data not shown), not unexpected because MSI is not frequent in clinical prostate cancer. Further, we tested 24 colorectal and 24 endometrial MSI patient cancer samples. We found *PRRT2* to be mutated in 15 out of 24 colorectal cancer patients and in 11 out of 24 endometrial cancer patients (Supplementary Figure 4, Supplementary Table 6). Although the mutation frequency in the *DAB2IP* MNR was similar (11/23), only one endometrium cancer sample displayed a mutation in the repeat. To further evaluate the significance of the mutation frequency of *PRRT2* and *DAB2IP* in clinical MSI colorectal and endometrial cancer patient samples, we compared their frequency to the average mutation frequency for common MSI target genes published in databases (SelTarbase [31]) (Supplementary Table 7). Compared to other genes, *PRRT2* displayed a very high mutation frequency in both colorectal and endometrial cancer and was selected for functional studies.

### The *PRRT2* gene

**Figure 3:**
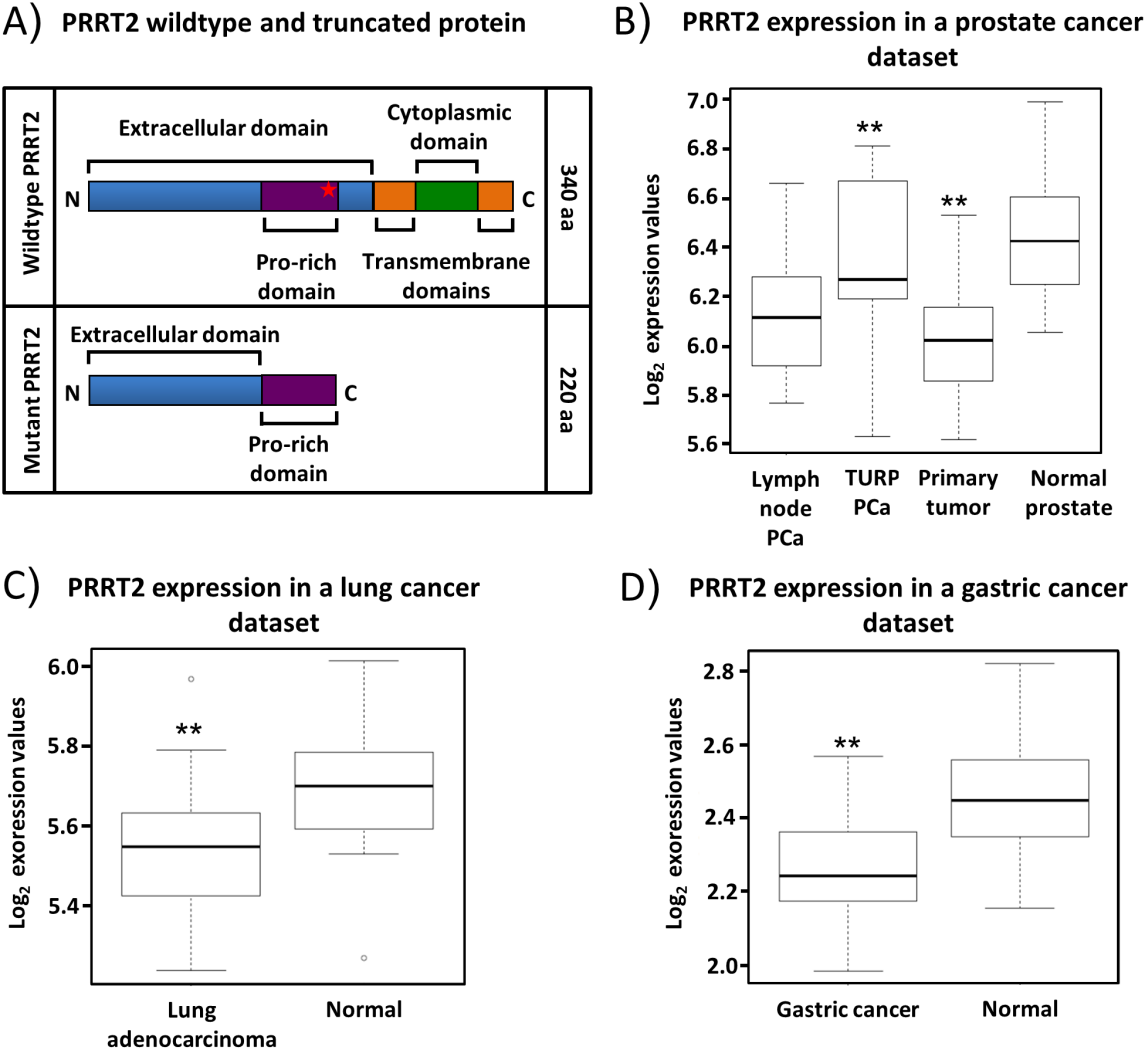
Expression pattern of PRRT2 mRNA **A**. Schematic representation of the wildtype and the mutant PRRT2. The insertion of C and CC in the 9C repeat of PC346C leads to a truncated protein lacking the predicted transmembrane and cytoplasmatic domains. The frameshift occurs within the proline-rich region of PRRT2. **B**. Box plot analysis of *PRRT2* mRNA in prostate cancer lymph node metastasis (LN-PCa, 12 samples), transurethral resections (TURP- PCa, 10 samples), primary prostate cancer (PCa, 56 samples) and normal adjacent prostate tissue (NAP, 12 samples). **C**. Expression levels of *PRRT2* in lung adenocarcinoma (22 samples) and normal lung tissue (22 samples). **D**. Expression levels of *PRRT2* in gastric cancer (20 samples) and normal gastric tissue (20 samples). Wilcoxon testing was used in (B), (C) and (D) to determine significant differences in expression levels.

*PRRT2* encodes a 340 amino acids protein that has two predicted transmembrane domains. This protein is poorly characterised, although recently a link between *PRRT2* mutations and a rare neurological disease, paroxysmal kinesigenic dyskinesia, was established [32]. Whether *PRRT2* has a role in cancer is unknown. Frameshifts in the C-repeat would result in expression of truncated proteins of 222 or 227 amino acids that lack the predicted transmembrane domains (Figure 3A). The PC346C prostate cancer cell line is the only cell line we identified, which has a mutation in both *PRRT2* alleles. To investigate a possible role of *PRRT2* in cancer we first documented *PRRT2* expression levels by RNA sequencing in available cancer data sets (Supplementary Materials and Methods). Overall, *PRRT2* showed a consistent decrease in expression in cancer as compared to normal in prostate, lung and gastric tissue samples (Figures 3B, 3C, 3D). Both lung and gastric cancers have been associated with MSI. In order to exclude non-mediated mRNA decay of the mutated *PRRT2* transcript we investigated the expression level of *PRRT2* in PC346C. The elevated expression level detected suggested that the stability of the *PRRT2* transcript is not affected by the mutation (Supplementary Figure 5).

### Biological activity of PRRT2

To assess whether the truncated PRRT2 (ΔPRRT2) influenced cell proliferation, we stable transfected the normal prostate epithelial cell line PNT2C2 with lentiviral constructs expressing wild type PRRT2 (PRRT2^wt^) or ΔPRRT2. Efficiency of transfection was assessed by the expression of GFP, independently transcribed under the CMV promoter. Cell proliferation was measured during 4 days after plating of equal numbers of control (GFP only), PRRT2^wt^ and ΔPRRT2 transfected cells.

**Figure 4:**
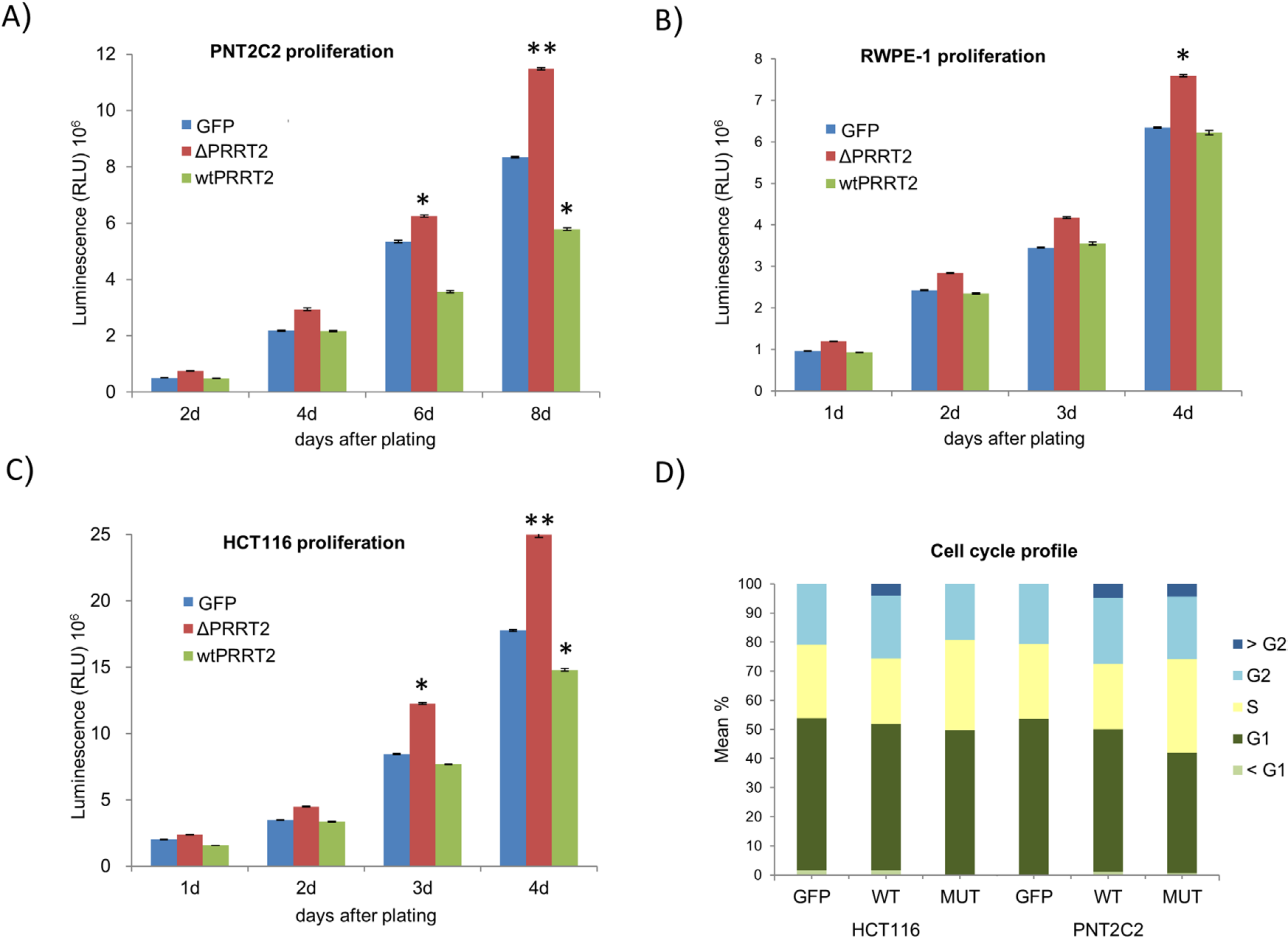
PRRT2 affects cellular proliferation **A**. PNT2C2, **B**. RWPE-1 and **C**. HCT116 cells were stably transduced with lentiviral vectors expressing the truncated form of PRRT2 (ΔPRRT2), the wildtype PRRT2 (wtPRRT2) and the control GFP vector. Cellular proliferation was measured 1, 2, 3 and 4 days after plating of the RWPE-1 and HCT116 cells, and 2, 4, 6 and 8 days after plating of the PNT2C2 cells. Cellular viability was measured by the reduction of luciferin into ocyluciferin in the presence of ATP. The y-axis displays the RLU (relative light units). Asterisks indicate *P≤0.02 and **P≤0.002, respectively, in a two-tailed Student's t-test. **D**. The cell cycle profile of HCT116 and PNT2C2 cells expressing GFP, ΔPRRT2 and wtPRRT2 was determined measuring PI staining levels by flow cytometry.

A significant increase in proliferation capacity of PNT2C2 cells expressing ΔPRRT2 (37.5%) was observed as compared to the control cells at day 8. Conversely, the growth of PNT2C2 cells expressing PRRT2^wt^ was impaired especially as compared to the PNT2C2 cells expressing ΔPRRT2 (Figure 4A). The ΔPRRT2 effect on cellular proliferation was further validated in the RWPE-1 prostate epithelial cell line and the HCT116 colorectal cancer cell line. Both cell lines displayed the same growth promoting effect of ΔPRRT2. RWPE-1 cells stably expressing ΔPRRT2 showed an increase of 20% in proliferation capacity whereas PRRT2^wt^ did not stimulate proliferation (Figure 4B). HCT116 cells expressing ΔPRRT2 showed an increase of 41% in proliferation capacity as compared to the control. Similar to PNT2C2, cells expressing PRRT2^wt^ showed a decrease in cellular proliferation (Figure 4C). The cell cycle analysis of HCT116 and PNT2C2 cells revealed an increase in the percentage of cells in S-phase when ΔPRRT2 is present (Figure 4D and Supplementary Table 8).

The influence of PRRT2 expression on apoptosis was addressed in a caspase 3/7 activation assay in all ΔPRRT2 and PRRT2^wt^ expressing cell lines described above. In none of the cases we observed a clear difference in apoptosis levels between ΔPRRT2, PRRT2^wt^ and control GFP expressing cells (data not shown).

**Figure 5:**
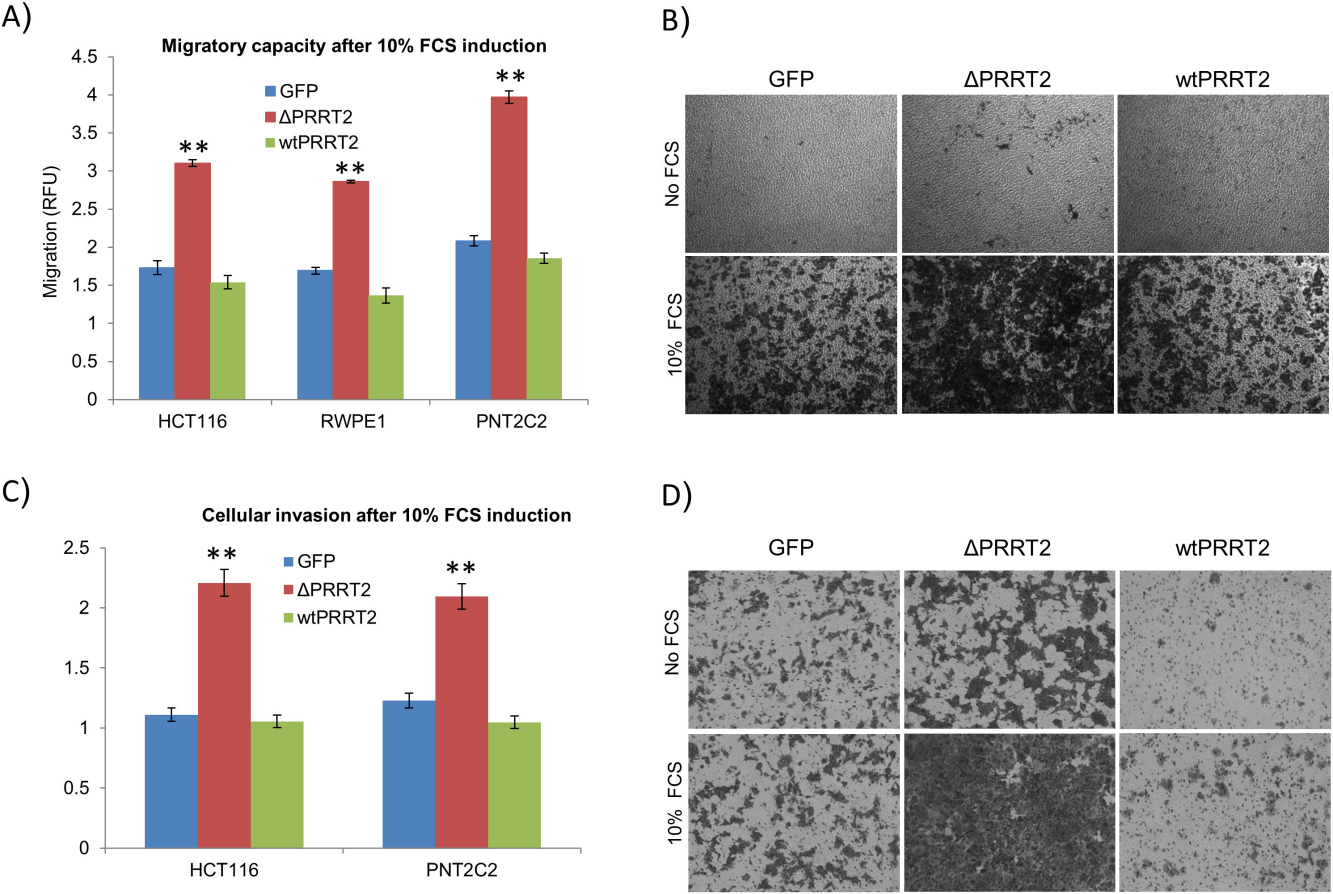
PRRT2 plays a role in cellular migration and invasion **A**. Migratory capacity of HCT116, PNT2C2 and RWPE-1 cells was assayed by measuring the cells migrating through a 5 μm polycarbonate membrane. Migration was induced by 10% FCS. Cells were assayed using CyQuant® GR fluorescent dye. All samples were normalized to the fluorescent detection in the absence of chemo attractant (10% FCS). RFU represents the relative fluorescent units. **P≤0.002 was calculated using a two-tailed Student's t-test. **B**. Representative illustrations of migration of HCT116 cells expressing ΔPRRT2, wtPRRT2 or control GFP in the presence and absence of 10% FCS (see also legend to Figure 4). **C**. Cellular invasion of HCT116 and PNT2C2 cells was assayed using collagen-coated membranes. Invasion was induced by 10% FCS. Cells were assayed using the same method as for migration. **D**. Representative illustrations of invasion of HCT116 cells expressing ΔPRRT2, wtPRRT2 or control GFP in the presence and absence of 10% FCS.

Finally we addressed the effect of PRRT2 on cell migration and invasion. PNT2C2, RWPE-1 and HCT116 expressing ΔPRRT2, PRRT2^wt^ and GFP were cultured in migration chambers for 24h in both presence and absence of 10% FCS. We detected a significant increase in migratory capacity of ΔPRRT2 expressing cells as compared to the GFP controls (Figure 5A and 5B). Migration of PRRT2^wt^ expressing cells was consistently lower than the GFP control cells, but in all cases, not significantly different. In order to determine whether PRRT2 also promotes cell invasion we cultured PNT2C2 and HCT116 cells expressing ΔPRRT2, PRRT2^wt^ and GFP in collagen invasion chambers during 24h. Again, ΔPRRT2 expressing cells were more invasive compared to GFP and PRRT2^wt^ expressing cells. (Figure 5C and 5D).

## DISCUSSION

### Microsatellite instability in cancer

The most prominent characteristic allowing cancer cells to survive, proliferate and disseminate is the development of genomic instability [33]. DNA repair mechanisms are the cells’ surveillance systems and defects in this machinery are responsible for accelerating the rate at which cells can accumulate favourable genotypes. The MMR system eliminates base-base mismatches and insertion-deletion loops that arise during DNA replication due to slippage of the DNA polymerase [1]. Simple repetitive DNA sequences known as microsatellites are particularly prone to insertions and deletions due to defective MMR [34]. As a result, cells with a defective MMR system might have mutation rates 100-1000 fold higher than normal cells [35]. Several studies have shown that instability of microsatellites is present in PCa [36, 37]. The frequency of microsatellite instability detected in PCa is variable between different studies and ranges from 8% [38] to 35% [39, 40]. To elucidate the role of MMR in PCa we have sequenced the complete genome of the MMR-deficient PC346C cell line. This cell line was derived from a transurethral resection of a primary tumor and lacks a functional MSH2 protein due to point mutations in both alleles [30].

The use of whole genome sequencing allowed an unbiased detection of all gene mutations and it is a powerful strategy to uncover genes implicated in the disease. Using this approach we identified a total of 1196 gene mutations in PC346C. Approximately one third of these mutations occurred in nucleotide repeat sequences (see also ref [41]). The total number of gene mutations detected in G089 was 1.5 fold lower than in PC346C, which fits to the mutational phenotype due to MMR deficiency. Mutations in MNRs were enriched by 6-fold in PC346C as compared to G089. The bias in MNR mutations in PC346C as compared to G089 was used to select for gene mutations triggered by the MMR system. Several of the downstream target genes of MMR deficiency have shown to be key players in proliferation and apoptosis pathways.

### Identification of novel MMR target genes in PCa

A list of 381 genes showing mutations in mononucleotide repeats in PC346C was used to select for novel target gene mutations caused by deficiency in the MMR system in PCa. Since the percentage of PCa cases with MSI was low we extended the test panel to include ovarian, endometrial and colorectal cancer cell lines. A final list of 13 candidate genes was further assessed for their mutation status in cell lines with and without microsatellite instability. We have first selected for the genes with the highest mutation frequencies in our prostate cancer cell line panel. Although *TSHZ2* showed the highest mutation frequencies for colorectal and ovarian cancer cell lines only one out of the four MSI prostate cancer cell lines was positive for the *TSHZ2* mutation. *CNOT1* was mutated in all MSI prostate cancer cell lines but this was, in all cases, an heterozygous mutation and occurred closed to the end of the coding region. Both *PRRT2* and *DAB2IP* were mutated in three out of the four MSI prostate cancer cell lines. Whereas *PRRT2* mutations were also frequent in both colorectal, endometrial and ovarian cancer cell lines *DAB2IP* mutations were mostly restricted to prostate and colorectal cancer samples. DAB2IP has recently been identified as a regulator of tumour growth and apoptosis. The expression of *DAB2IP* is often downregulated in PCa and this downregulation causes activation of the RAS signalling cascade and inactivation of the ASK1-JNK pathway leading to growth stimulation and suppression of apoptosis [42, 43]. The observation that *DAB2IP* is mutated at high frequency in MMR deficient prostate and colorectal cell lines indicates a link between MMR and this gene. But so far, epigenetic suppression was suggested as the major mechanism behind *DAB2IP* downregulation [44]. Truncating mutations in PRRT2 were first identified in 2011 by Chen WJ *et al*. in paroxysmal kinesigenic dyskinesia (PKD) [32]. Although PRRT2 appears to be an important factor in several neurological-related conditions [45] there is no correlation between PRRT2 and other diseases. Also, there is little functional data regarding the PRRT2 protein with only one protein-protein interaction known so far with the synaptosomal-associated protein 25 (SNAP25) [46].

### PRRT2 and mismatch repair deficiency

The mutation status of *PRRT2* and *DAB2IP* was assessed in a cohort of 24 colorectal and 24 endometrial MSI patient samples. We observed a high number of patients with mutations in *PRRT2* and to a lesser extent mutations in *DAB2IP* were detected. The finding that *DAB2IP* was mutated in only one endometrial cancer sample indicates tissue specificity for this particular gene mutation. *PRRT2* was frequently mutated in both colorectal and endometrial cancer patients. The frequency of *PRRT2* mutation in patient samples is only second to the frequency of TGFβRII in colorectal cancer and the highest in endometrial cancer of all MSI genes (Supplementary Table 7) [47]. Although most targets of MMR deficiency are considered to be tumor suppressor genes that through the frameshift outcome lose their function there are also studies showing activating mutations such as the frameshifts in the *TCF4* gene [48]. The two *PRRT2* frameshift mutations found in PC346C, the insertion of one or two Cs, occur in a C9 repeat in the second coding exon and introduce a premature stop codon in the mRNA. As a consequence, the PC346C cell line lacks a wild type PRRT2 protein. Although it is claimed that the non-sense mediated mRNA decay (NMR) mechanisms recognises premature stop codons and eliminate aberrant transcripts [49] we found a high level of expression of the *PRRT2* transcript in the PC346C cell line. The expression level of *PRRT2* is overall lower in cancer as compared to normal, which would indicate it as a potential tumour suppressor gene. However mutations in this gene only affect one of the copies in almost all affected cancers, leaving a wild type *PRRT2* copy intact. The PC346C prostate cancer cell line and patient from which the cell line is derived are the only samples with homozygous mutations in the 9C repeat. Despite the lower expression of *PRRT2* in cancer versus normal tissue our data suggests a dominant negative effect of the truncated PRRT2 protein

### PRRT2 in proliferation and migration

To investigate the functional role of both the wild-type (PRRT2^wt^) and the truncated PRRT2 protein (ΔPRRT2) we generated stable PRRT2^wt^ and ΔPRRT2 expressing cell lines. Two normal prostate cell lines (PNT2C2 and RWPE-1) and one colorectal cancer cell line (HCT116) were used to assess the effect of PRRT2 in proliferation, apoptosis and migration. Remarkably, we observed an oncogenic effect of ΔPRRT2. Both proliferation, migration and invasion were enhanced by stable ΔPRRT2 expression. Also, the cell cycle profile showed an enrichment of cells in S-phase and lesser cells in G1 suggesting these cells are cycling faster. Conversely, PRRT2^wt^ appeared to decrease the proliferation rate, being this effect more evident in the PNT2C2 and HCT116 cell lines as compared to RWPE-1. This decrease in proliferation by PRRT2^wt^ overexpression suggests PRRT2 to participate in cell cycle regulation. The frameshift mutation creates a truncated PRRT2 protein that increases both proliferation and migration in cell lines with a wild type PRRT2 protein. Based on our findings, we hypothesize that wild type *PRRT2* inhibits slightly proliferation, whereas truncated PRRT2 can stimulate, and overrules wild type PRRT2. In this way it can function as an oncogene by promoting both proliferation and migration. Although genes are usually dichotomized in either oncogenes or tumor suppressors [50] many can actually exert both functions such as the *Tp53* tumor suppressor gene which can lead to tissue invasion, metastasis and increased proliferation when mutated [51].

In conclusion, a focused genome-wide sequencing approach, followed by subsequent expression and functional studies indicate mutated *PRRT2* as a novel dominant oncogene, and wt*PRRT2* as a candidate tumor suppressor gene. The finding that the same *PRRT2* mutation detected in this study has also been documented in colorectal, pancreatic and stomach cancer samples (depicted as A214P in Supplementary Figure 6) supports a more general role of PRRT2 in cancer biology.

## MATERIALS AND METHODS

### DNA samples used in whole genome and data analysis

The DNA of two prostate cancer samples, the PC346C prostate cancer cell line [52] and the G089 prostate cancer patient, were sequenced by Complete Genomics (Complete Genomics Inc, CA). Tissue sample G089 and other patient materials described below has been collected according to national legislation concerning ethical requirements. Use of the clinical samples has been approved by the Erasmus MC Medical Ethics Committee according to the Medical Research Involving Human Subjects Act (MEC-2004-261). NCBI build 36 (hg18) was used as a reference genome during the mapping and data analysis process. Complete Genomics Inc. pipeline generated a report with all single nucleotide variants present in the two samples. Further information on the Complete Genomics Inc. data processing is present in supplementary materials and methods.

### DNA from additional cell lines and tissue samples

Additional genomic DNA was isolated from MMR-proficient prostate cancer cell lines PC3, PC135 and PC295 and MMR-deficient prostate cancer cell lines DU145, LNCaP and PC374. Cell pellets from thirteen colorectal, seven endometrial and three ovarian cancer cell lines were kindly provided by Dr. W. Dinjens, Dept Pathology, Erasmus MC, Rotterdam, The Netherlands.

Also, genomic DNA was isolated from frozen 31 primary prostate cancer patient tissues, 37 transurethral resection of the prostate (TURP) samples and 11 lymph node prostate cancer metastasis samples. Genomic DNA from paraffin embedded formalin fixed tissue samples of 24 microsatellite instable colorectal cancer patients and 24 microsatellite instable endometrial cancer patients were kindly provided by Dr. W. Dinjens.

### DNA isolation

DNA isolation from prostate cancer samples was performed using the QIAamp DNA Blood Midi Kit (Qiagen) according to the manufacturers’ instructions. Cell pellets were resuspended in 1 ml PBS (BioWhittaker) and instructions were followed according to the protocol used for 1 ml whole blood. DNA was eluted in 200 μl elution buffer.

DNA isolation from the colorectal, endometrial and ovarian cancer cell lines pellets was performed using the Gentra Puregene Cell Kit (Qiagen) (approx. 15 μg DNA from 2×106 cells) following the manufacturer's protocol.

Concentration and purity of the DNA was assessed using the NanoDrop ND 1000 spectrophotometer (Nanodrop) by absorption measurements at 260 nm. DNA was stored at -20 degrees Celsius.

### RNA isolation

Total RNA was isolated from PC346C, RWPE-1, VCaP, HCT116 and PNT2C2 using the RNeasy kit (Qiagen) according to the manufacturer's protocol. RNA was eluted in 50 μl RNase free water. Concentration and purity of RNA was assessed using the NanoDrop ND 1000 spectrophotometer (Nanodrop) by absorption measurements at 260 nm. RNA was stored at -80 degrees Celsius.

### Cell culture

The PC346C cell line was cultured in DMEM-F12 (BioWhittaker), supplemented with 2% (V/V) FCS (PAN Biotech), 1% insulintransferrin-selenium (GIBCO BRL), 0.01% BSA (Boehringer-Mannheim), 10 ng/ml epidermal growth factor (Sigma-Aldrich) and 500 U penicillin-streptomycin (BioWhittaker), 100 ng/ml bronectin (Harbor Bio Products), 20 mg/ml fetuine (ICN Biomedicals), 0.1 nM R1881 (Sigma-Aldrich), 50 ng/ml cholera toxin (Sigma-Aldrich), 0.1 mM phosphoethanolamine (Sigma-Aldrich), 0.6 ng/ml triodothyronine (Sigma-Aldrich), and 500 ng/ml dexamethasone (Sigma-Aldrich). The PC346C cell line expresses the wild-type androgen receptor and secretes high levels of PSA. RWPE-1 cells were cultured in keratinocyte medium (GIBCO BRL), supplemented with 5 ng/ml epidermal growth factor, 1% penicillin-streptomycin (BioWhittaker), and 50 mg/L bovine pituitary extract. RWPE-1 is a normal prostate epithelium cell line that is androgen-independent and expresses both the androgen receptor and PSA. The colorectal cancer cell line HCT116 and the normal prostate cell line PNT2C2 cells were cultured in RPMI-1640 (GIBCO BRL), supplemented with 10% FCS (PAN Biotech) and 1% penicillin-streptomycin (BioWhittaker). All cell lines were cultured at 37 degrees in a 5% CO2 atmosphere.

### Mononucleotide repeat analysis

A microsatellite instability assay was used to detect changes in the length of mononucleotide repeats by PCR amplification. Primers were designed using the Primer3Plus program (http://www.bioinformatics.nl/cgi-bin/primer3plus/primer3plus.cgi). The final fragment size was set to vary between 100 and 130 bp in order to allow precise fragment sizing and robust amplification of all the genes tested. Primer sequences are described in Supplementary materials and methods. The M13 sequence (GTAAAACGACGGCCAGT) was added at the 5’ end of each forward primer. This enabled the use of a M13-FAM primer to fluorescently label each PCR fragment. PCR reactions were performed in a total volume of 16 μl containing 12.5 ng genomic DNA, 3 μl 10x PCR Buffer (Quiagen), 2 μl 5x Q-Solution (Quiagen), 0.9 μl 25 mM MgCl2 (Quiagen), 0.3 μl dNTPs (10mM), 0.5 μl forward primer (100ng/μl), 0.5 μl M13-FAM primer (100ng/μl), 1 μl reverse primer (100ng/μl), and 1 U HotStarTaq Polymerase (Quiagen). An initial denaturation step of 15 min at 95 degrees Celsius was used to activate the HotstarTaq, followed by 35 cycles denaturation at 95 degrees Celsius for 30 sec, an annealing step at 54 degrees Celsius for 30 sec and elongation at 72 degrees Celsius for 1 min. A final elongation step at 72 degrees Celsius for 10 min was used. PCR products were checked by electrophoresis in a 1% agarose gel.

### Fragment analysis

The PCR products were separated by capillary electrophoresis using an ABI PRISM 3130 sequencing analyzer. This method allows single base pair resolution of PCR fragments. A mixture of 1.5 μl of the PCR products with 9.8 μl highly-deionized formamide (HiDi) and 0.2 μl GeneScan-500 LIZ size standard (Applied Biosystems) was prepared. This mixture was heated for 2 min at 95 degrees Celsius and subsequently loaded to the sequence analyzer. Data was analyzed with the Peak Scanner software (Applied Biosystems).

### Sequencing

PCR products have been sequenced bi-directionally using standard Sanger sequencing. The sequencing reactions were carried out using the same forward and reverse primers as indicated above in the PCR but at different concentrations (3 ng primer per reaction). The final reaction volume was 20 μl. The sequencing products were precipitated using isopropanol and resuspended in 20 ul formamide (Sigma-Aldrich). The PCR product was sequenced on an ABI Model 3730 automated sequencer and analyzed using DNAMAN (Lynnon Corporation).

### PRRT2 expression constructs

The sequence validated I.M.A.G.E clone IRATp970E0579D corresponding to the BC053594 clone accession number was purchased from Source Bioscience, Nottingham, UK. This plasmid contains the wt*PRRT2* cDNA sequence. Primers 5’ GAT CGA ATT CGT TTG CCG CTG TCT CT 3’ (Fw) and 5’ GAT CGC GGC CGC TCA CTT ATA CAC GCC 3’ (Rv) were used to amplify the wt*PRRT2* cDNA and subsequently clone the fragment into the pCDH-CMV-MCS-EF1-GFP-T2A-Puro vector (System Biosciences). Primers 5’ GAT CGA ATT CGT TTG CCG CTG TCT CT 3’ (Fw) and 5’ GAT CGC GGC CGC CCC TTC TCA TTC GAT 3’ (Rv) were used to generate a truncated *PRRT2* cDNA fragment. This fragment was integrated into the pCDH-CMV-MCS-EF1-GFP-T2A-Puro vector (System Biosciences) for expression of ΔPRRT2 protein. The amplified fragments were ligated to the vector using the Rapid DNA Ligation Kit according to the manufacturer's protocol (Roche). JM109 competent bacteria (Promega) were transformed with the ligation products using the heat shock method and plated in Ampicillin LB agar plates. DNA isolation was performed on selected colonies (Quiagen) and restriction enzyme reactions (BamH^I^ and Not^I^ ) confirmed the proper ligation of fragment to vector.

### Lentivirus production

HEK293T cells were co-transfected with pCDH-wtPRRT2-GFP-Puro or pCDH-ΔPRRT2-GFP-Puro or pCDH-GFP-Puro expression vectors and pPAX2 and pMD2.G (kind gift of Prof Didier Trono, Switserland) using the calcium phosphate precipitation method. Virus was harvested from the supernatant and used for transduction of PNT2C2, RWPE-1 and HCT116 cells. Pools of infected cells were propagated.

### Functional role of PRRT2

The functional role of PRRT2 was assessed in RWPE-1, HCT116 and PNT2C2 cell lines expressing the truncated form of *PRRT2* (ΔPRRT2), the wildtype *PRRT2* (wtPRRT2) and the control GFP. These cell lines were propagated in medium supplemented with 1 μg/mL puromycin (Millipore). Briefly, cells were plated in 96-well culture plates at a density of 1000 cells per well (100 μl). Proliferation was assessed using the CellTiter-Glo Luminescent Cell Viability Assay (Promega), according to manufacturer's protocol. Cell cycle analysis was performed by propidium iodide staining of the cell lines involved. Following cell pellet collection, fixation with 70% ethanol was performed. Fixated cells were washed in PBS-0.05% Tween20 and ressuspended in 500 μL of PBS-0.05% Tween20, RNases and PI. Samples were measured in FACS calibre following a 15 minute incubation with PI. Apoptosis was assessed using the ApoLive-Glo™ Multiplex Assay (Promega), which measures both the number of viable cells as a marker of cytotoxicity and caspase activation as a marker of apoptosis within a single assay well. The assay was performed according to the manufacturer's protocol. As positive control of apoptosis an apoptosis inducer set (Millipore) was used. A mix containing 700x dilutions of Actinomycin D (10 mM), Camptothecin (2 mM), Cycloheximide (100 mM), Dexamethasone (10 mM) and Etoposide (10 mM) was supplemented for 24h to induce apoptosis. The QCM Chemotaxis Cell Migration Assay, 96-well (8 μm), fluorimetric (Millipore) was performed according to the manufacturer's protocol. CytoSelect™ 24-Well Cell Invasion Assay, Collagen I was used to determine cell invasion according to the manufacturer's protocol.

FP7 Marie Curie Initial Training Network PRO-NEST (grant number 238278)

## SUPPLEMENTARY MATERIALS FIGURES AND TABLES






