# Supplementary material for: A mononucleotide repeat in PRRT2 is an important, frequent target of mismatch repair deficiency in cancer
Source: Oncotarget. 2016 Nov 19;8(4):6043–56. doi: 10.18632/oncotarget.13464 (PMC5351611; doi:10.18632/oncotarget.13464)
Supplement: Supplementary file 1 [file oncotarget-08-6043-s001.pdf]

A mononucleotide repeat in *PRRT2* is an important, frequent target of mismatch repair deficiency in cancer

SUPPLEMENTARY DATA

SUPPLEMENTARY MATERIALS AND METHODS

DNA sequencing

Paired-end sequencing of the G089 and PC346C DNA samples was performed with the Complete Genomics service provider using a proprietary sequencing-by-ligation technology and primary data analysis, including image analysis, base calling, alignment and variant calling [1]. Reads were mapped to the NCBI Build 36.1 reference genome and mappings were expanded by local de novo assembly on all regions of the genome that contain single nucleotide variations (SNVs) relative to the reference genome [1]. SNVs, insertions and deletions (indels) and substitutions are reported in the variation files. Additional information on the nomenclature used by Complete Genomics can be found in 'http://www.completegenomics.com/FAQs/Variant-Calls-SNPs-and-Small-Indels/'.

Public exon array datasets

We used a publicly available dataset of Affymetrix Human Exon Arrays to determine the expression levels of PRRT2. The prostate cancer dataset contains 48 previously published prostate cancer samples (GSE41408, [2]) as well as additional cancerous and control samples, accessible via GEO accession number GSE59745. The datasets comprised samples from normal adjacent prostate (NAP), localized prostate cancer obtained via radical prostatectomy (PCa) and transurethral resection of the prostate (TURP), as well as metastasis in lymph node (LN PCa). Public datasets of lung cancer (GSE12236, [3]) and gastric cancer (GSE13195) were used to confirm the expression pattern of PRRT2.

REFERENCES

1. Carnevali P, et al., Computational Techniques for Human Genome Resequencing Using Mated Gapped Reads. Journal of Computational Biology, 2012. 19: p. 279-292.
2. Boormans JL, et al., Identification of TDRD1 as a direct target gene of ERG in primary prostate cancer, Int J Cancer. 2013; 133:335-345
3. Xi L, et al., Whole genome exon arrays identify differential expression of alternatively spliced, cancer-related genes in lung cancer. Nucleic Acids Res. 2008; 36:6535-6547

Table 1: Primer sequences for microsatellite analysis

Gene symbol	Primer	Sequence: 5’ - 3’	Fragment length (bp)
PRRT2	Forward	CTCACTCACCACCCTCAAAAA	107
	Reverse	TTCTCATTCGATCCTCCTCAAC	
TSHZ2	Forward	AAGCACGCTCTGTCTGACATC	130
	Reverse	CTGACATCCATTTCCAGCTTC	
ANLN	Forward	GTGATTGTTCGGAAGGAAGATG	135
	Reverse	AAGCCCCTTTCCACAAAAGTA	
CEP164	Forward	AACACTATCGGAGCTTGGTGAT	126
	Reverse	GGGGTCTCTGTCCTTCTTGTCT	
CNOT1	Forward	CTGTCAAACATCGTGTTGAGAAT	128
	Reverse	CCATATTTGTTTGATGTCTTCCA	
KCNMA1	Forward	CCTCTCCTTACCTCATCAGCTT	121
	Reverse	CCGTCAACACTATCACCAAAAA	
ANUBL1	Forward	CCAGCATTTTCAGGAAGAAAAC	119
	Reverse	ACTACTAGAGCATGTTGAAAGGAAAA	
EPRS	Forward	ACATCAGGGTCAAAGGAAAAGA	120
	Reverse	ACCCTGGGATAGAAAGACCATT	
TTC3	Forward	TCAGCCTAGAGAAACTAAGACTGAAA	115
	Reverse	TCCTCCTCCATTCTTTCTTGTG	
SFRS12IP1	Forward	CAGGAATAAATGAAGAAGAGGAAA	134
	Reverse	CTCATAGGCCTGCTTTGTTTTT	
SFR	Forward	TGAAGCAAAATGTTTCCTTTTTA	125
	Reverse	GCTGGTATTTTGTGAGGCTTTC	
DAB2IP	Forward	GCGCAGTTGTTAGAAGACGAG	124
	Reverse	CCAGGGTCCAGTTTTACCTTTT	
PHACTR4	Forward	AAGAGCAAGTTCTCAGGCTTTG	117
	Reverse	CCCTTAATATCTCACCTTCTGAAGTC	
MLL3	Forward	TCAGTGTGTATCTGTTGAACCAAA	133
	Reverse	ACTTCCGTTTTTACCTCATTGG	
PDS5A	Forward	CAATGCAGATTCACCAAAGGAC	94
	Reverse	TGTGGTAGGAAAAATGAGAGGAA	
TROVE2	Forward	TTTGCCTTTTTGTTAGGTTTCC	166
	Reverse	AGAACCGGTGTAGTCGATTCAT	
USP42	Forward	CTGACCTCCACAGACACAAAAA	116
	Reverse	ACCCTGGGTAAGTGCAGTTCT	

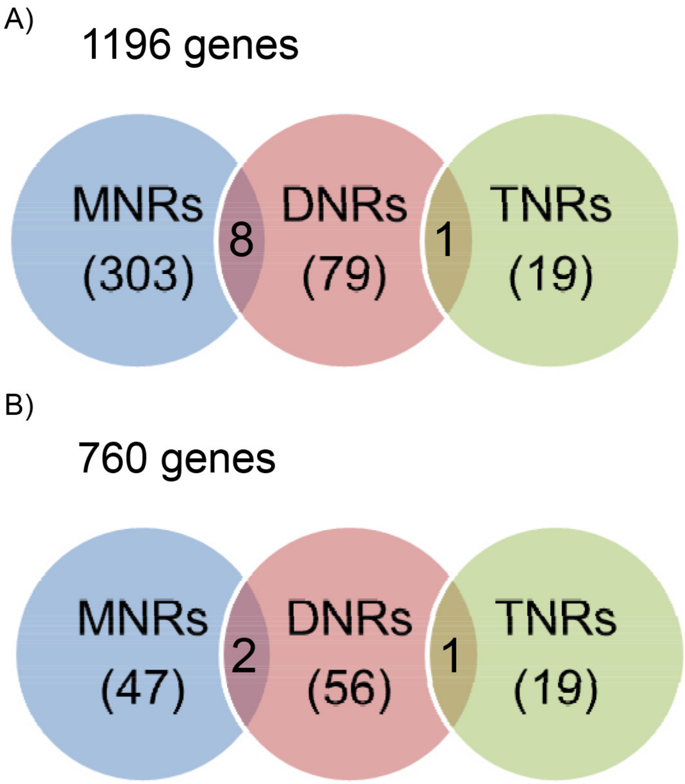

Supplementary Figure 1: Distribution of mononucleotide (MNR), dinucleotide (DNR) and trinucleotide (TNR) repeat mutations of length 6 nucleotides of MNRs and DNRs and length of 9 nucleotides for TNRs in affected genes. A. represents the distribution in the PC346C DNA and **B.** in G089 DNA. The numbers in the intersection of the different categories represent genes with both NR (either MNR and DNR or DNR and TNR). No genes had both MNR and TNR.

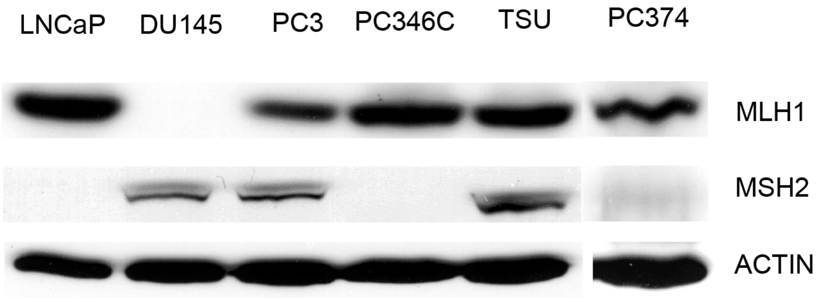

Supplementary Figure 2: Western blot analysis of the MLH1 and MSH2 protein expression in the MMR deficient prostate cancer cell lines LNCaP, DU145 and xenograft transplant PC374 and in the MMR proficient prostate cell lines PC3 and TSU. Actin is displayed as a loading control.

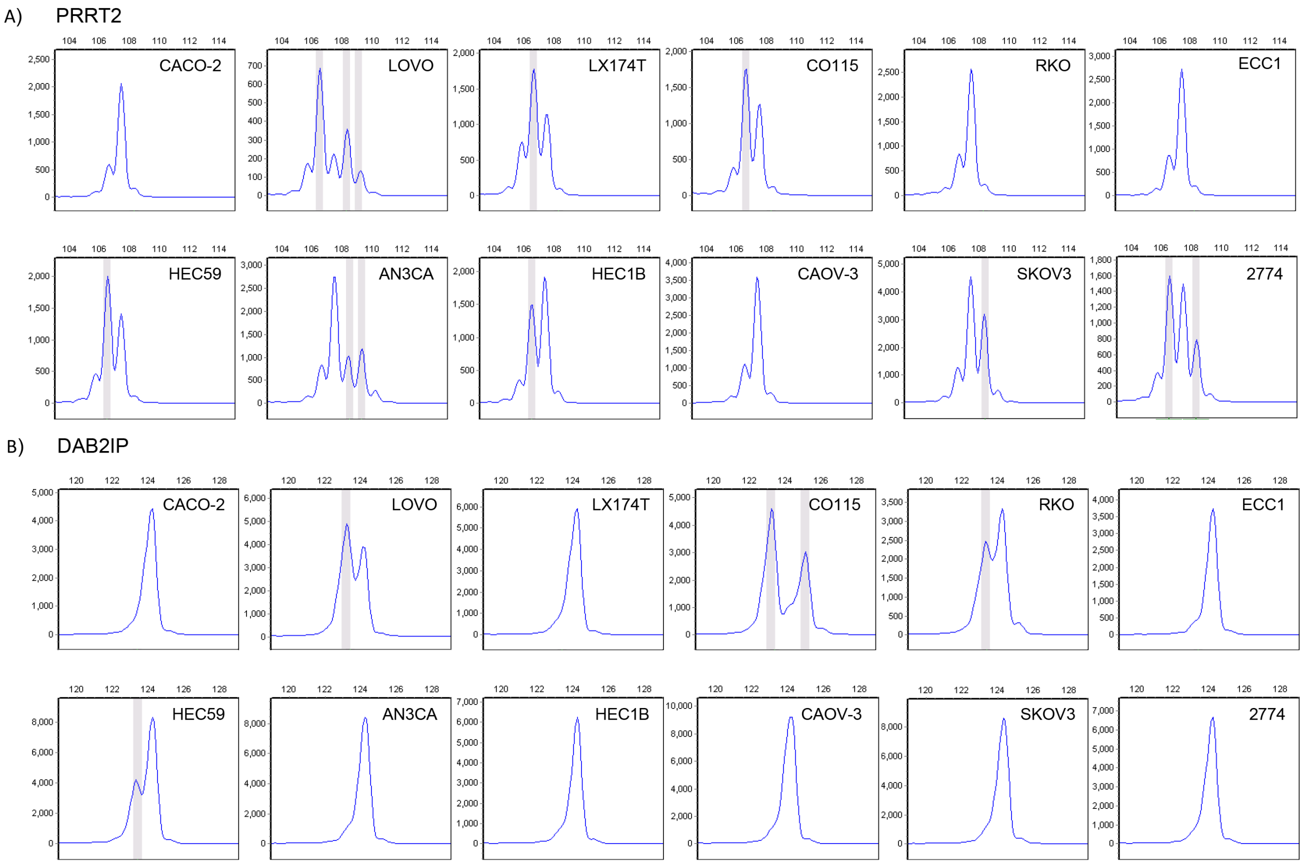

Supplementary Figure 3: Analysis of *PRRT2* A. and *DAB2IP* B. PCR fragments containing repeat sequences (see also Figure 2) in colorectal, endometrial and ovarian cancer cell lines. Samples 1 to 5 are colorectal cancer cell lines, with sample number 1 corresponding to the MMR proficient cancer cell line CACO2 is MMR proficient colon cancer cell line; LOVO, LX174T, CO115 and RKO are MMR deficient colon cancer cell lines, respectively. ECC1 is a MMR proficient endometrial cancer cell line; HEC59, AN3CA and HEC1B are MMR deficient endometrial cancer cell lines, respectively. CAOV-3 is a MMR proficient ovarian cancer cell line; SKOV3 and 2774 are MMR deficient ovarian cancer cell lines, respectively. The highlighted peak represents a mutant allele. The Y-axis corresponds to the intensity of the fluorescent peak. The X-axis represents the fragment size in base pairs. Shoulder peaks with intensities less than 32% of the highest peak were discarded from further analysis. PCR primers are given in Supplementary Materials and Methods.

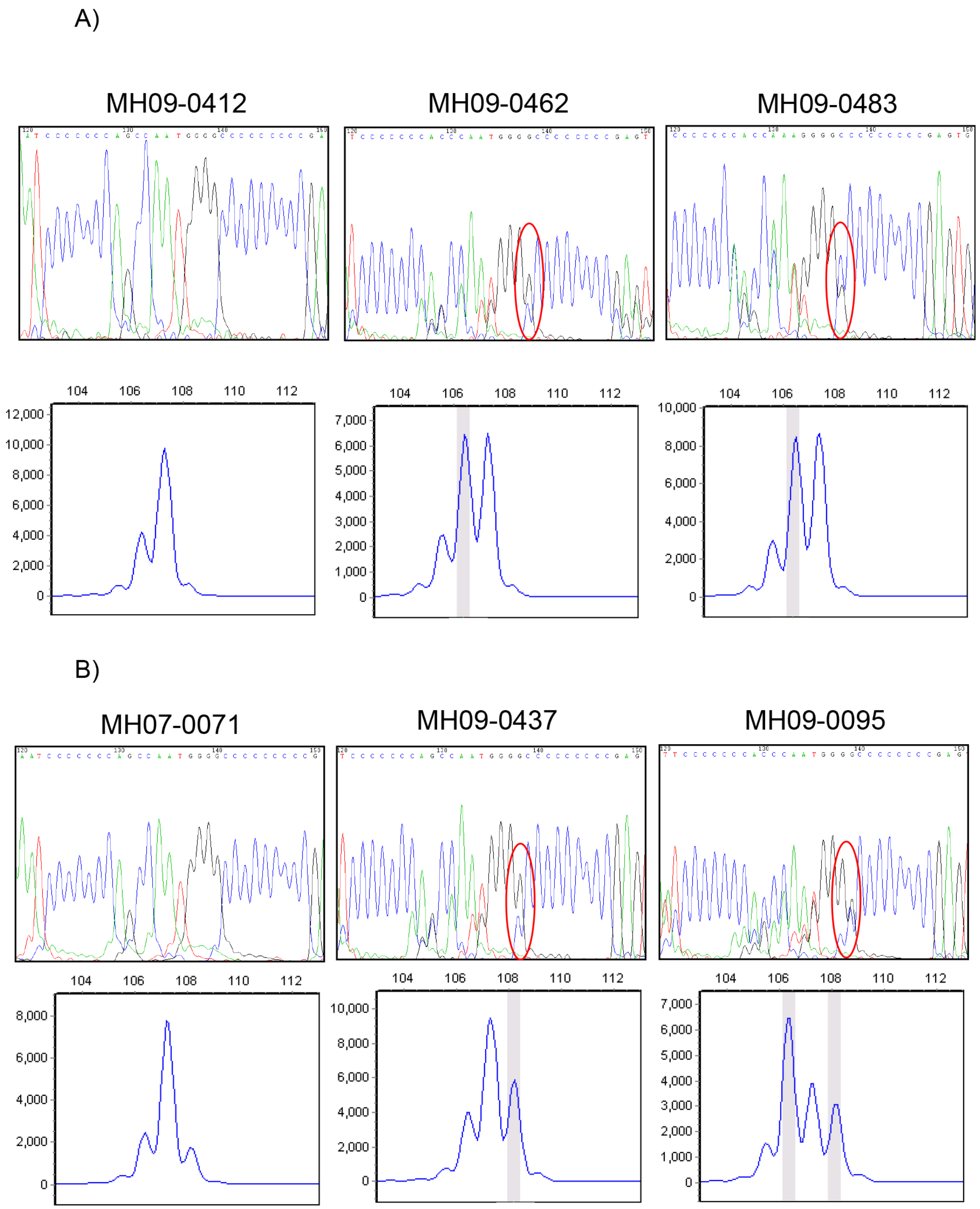

Supplementary Figure 4: Analysis of the *PRRT2* PCR fragment containing the C9 repeat sequence and repeat sequence analysis of MSI colorectal and endometrial cancer patient samples. A. Chromatogram and fragment analysis of three colorectal cancer patients. **B.** Chromatogram and fragment analysis of three endometrial cancer patients. The highlighted peaks (grey outline and red circle) represent mutant alleles. The wildtype 9 C repeat shows a prominent 107 bp fragment and a shoulder peak at length 106 bp. The intensity of shoulder peaks are less than 32% the intensity of the highest peak. Both MH09-0462 and MH09-0483 display peaks of equal intensity at length 106 and 107. The peak at 106 bp is therefore highlighted as a mutant allele in the two samples mentioned above. In (B) MH09-0095 has a lower peak corresponding to the wt 107 fragment. Fragments of 106 and 108 bp are also present in this sample.

A)

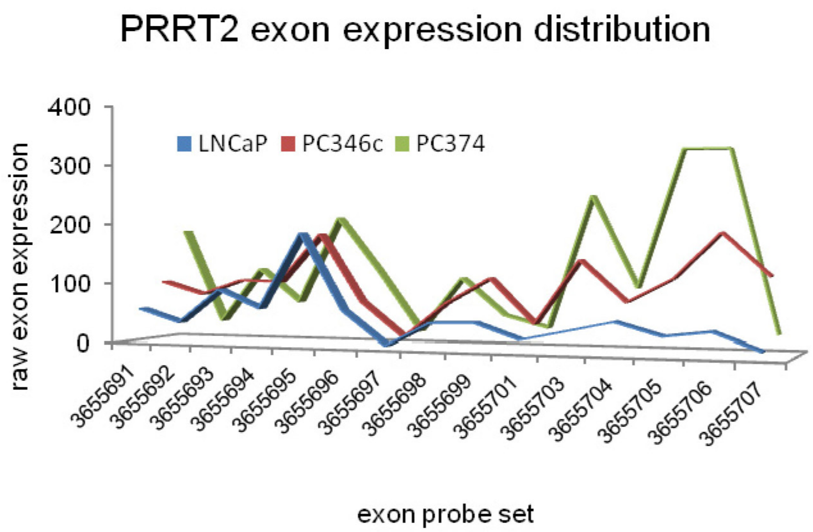

B)

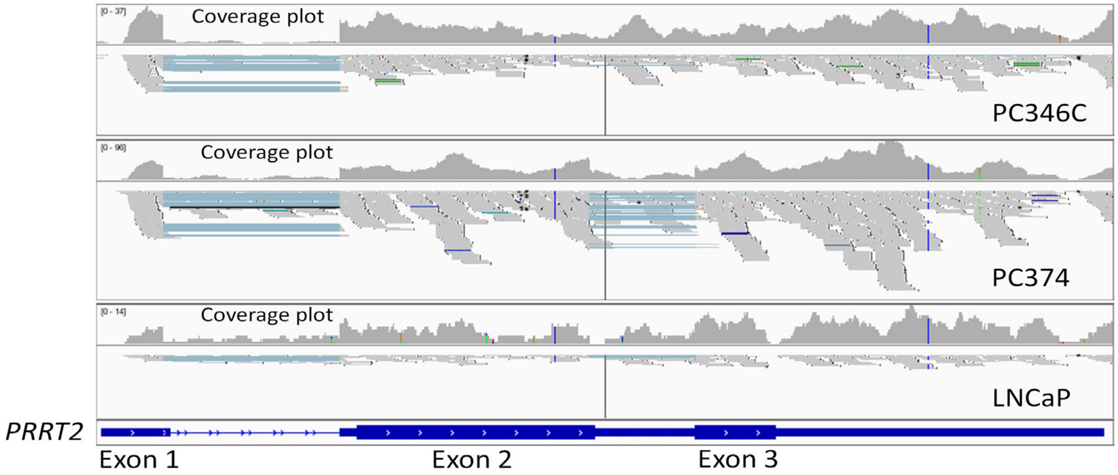

Supplementary Figure 5: Expression of the *PRRT2* transcript in PC346C, PC374 and LNCaP. A. Raw expression values of the exon probes across the *PRRT2* transcript. **B.** RNAseq read distribution of the *PRRT2* transcript. The reads mapping to PRRT2 are plotted bellow the coverage plot. The upper panel represents PC346C, the middle panel PC374 and the lower panel LNCaP. *PRRT2* exons are depicted in blue at the end of the figure.

(A)

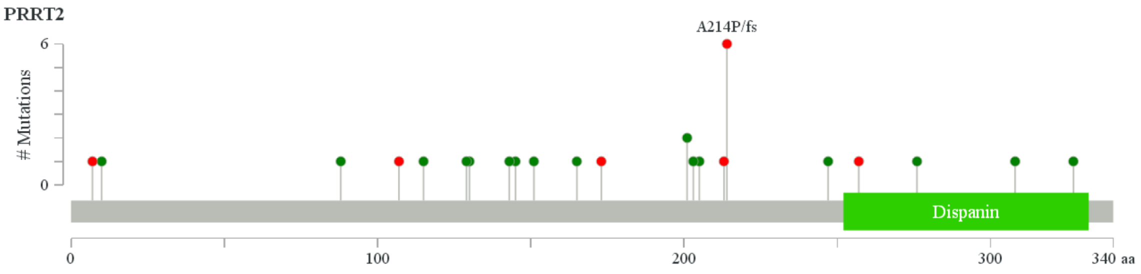

(B)

Sample ID	Cancer Study	AA change	Type	Copy #	COSMIC	#Mut in Sample
TCGA-AA-3715-01	Colorectal (TCGA pub)	A214fs	FS del	diploid	2	1490
TCGA-AA-3715-01	Colorectal (TCGA)	A214fs	FS del	diploid	2	1480
MD-339	MBL (Broad)	A214P	Missense	NA	2	9
TCGA-HV-A5A5-01	Pancreas (TCGA)	A214fs	FS ins	diploid	2	166
TCGA-FZ-5924-01	Pancreas (TCGA)	A214fs	FS del	diploid	2	54
TCGA-IB-7654-01	Pancreas (TCGA)	A214fs	FS del	gain	2	198
TCGA-BR-7703-01	Stomach (TCGA)	A214P	Missense	diploid	2	602

Supplementary Figure 6: Spectrum of somatic alterations in *PRRT2*. The cBIOPortal for cancer genomics [http://www.cbioportal.org/public-portal/] was used to catalogue all the genetic alterations found in the *PRRT2* gene across several cancer datasets. **A.** Diagram of all mutations catalogued in the *PRRT2* gene. Red corresponds to truncating mutations and green to missense mutations. The A214P frameshift corresponds to the mutation also found in the PC346C cell line. **B.** List of the samples containing the A214P mutation. This mutation has additionally been detected in 3 pancreatic adenocarcinoma samples, 1 colorectal adenocarcinoma sample, 1 stomach adenocarcinoma sample and 1 medulloblastoma sample.

Supplementary Table 1: Gene mutations identified in the PC346C sample (except missense mutations). Type includes SNP, deletions (del), insertions (ins), reference (ref) and no call (-). Impact includes misstart (MS), frameshift (FS), disrupt (D), nonsense (NS) and nonstop (N). Position is defined based on the start of the small variant (SV) call. Additional information on the output from complete genomics can be found on (http://www.completegenomics.com/FAQs/Variant-Calls-SNPs-and-Small-Indels/)

See Supplementary File 1

Supplementary Table 2: Gene mutations identified in the G089 sample (except missense mutations). Type includes SNP, deletions (del), insertions (ins), reference (ref) and no call (-). Impact includes misstart (MS), frameshift (FS), disrupt (D), nonsense (NS) and nonstop (N). Position is defined based on the start of the small variant (SV) call. Additional information on the output from complete genomics can be found on (http://www.completegenomics.com/FAQs/Variant-Calls-SNPs-and-Small-Indels/)

See Supplementary File 1

Supplementary Table 3: Distribution of mutated genes in PC346C and G089 and in all UCSC hg18 genes in MNR categories of different type and length

	PC346C (1196 genes)						G089 (760 genes)						UCSC genes					
	n_6	n_7	n_8	n_9	n_{10}	n_{12}	n_6	n_7	n_8	n_9	n_{10}	n_{12}	n_6	n_7	n_8	n_9	n_{10}	n_{12}
A	96	78	54	29	19	4	13	11	7	4	4	0	15100	9501	5768	4025	3013	1880
T	84	66	44	25	15	1	12	7	5	2	2	0	14617	9690	6358	4672	3616	2359
C	68	33	15	5	1	0	10	6	4	0	0	0	8636	2106	522	225	128	44
G	55	26	8	3	1	0	12	9	5	3	2	0	7468	1630	423	157	83	24

The subscript number represents the total nucleotide length of the repeat.

Supplementary Table 4: Allele pattern of microsatellite instability gene targets in MMR-deficient and -proficient prostate cancer cell lines

Gene	MMR-proficient (MSS)			MMR-deficient (MSI)			
	PC3	PC295	PC135	DU145	LNCaP	PC346C	PC374
CNOT1	wt	wt	wt	m1wt	m1m2wt	m1m2wt	m1wt
DAB2IP	wt	wt	wt	wt	m1wt	m1wt	wtp1
PRRT2	wt	wt	wt	wtp1p2	wt	p1p2	wtp1
TTC3	wt	wt	wt	m1wt	wt	m1wt	m1wt
CEP164	wt	wt	wt	wt	m1wt	m1wt	m1wt
PHACTR4	wt	wt	wt	wt	m1wt	m1wt	m1wtp1
TROVE2	wt	wt	wt	wt	wt	m1wt	m1wt
ZFR	wt	wt	wt	wt	m1wt	m1wt	wt
MLL3	wt	wt	wt	wt	m1wt	m1wt	wt
ANLN	wt	wt	wt	wt	wt	m1wt	wt
ANUBL1	wt	wt	wt	wt	wt	m1wt	wt
EPRS	wt	wt	wt	wt	wt	wtp1	wt
KCNMA1	wt	wt	wt	wt	wt	m1wt	wt
PDS5A	wt	wt	wt	wt	wt	m1wt	wt
SFRS12IP1	wt	wt	wt	wt	wt	wtp1	wt
TSHZ2	wt	wt	wt	wt	wt	m1wt	wt
USP42	wt	wt	wt	wt	wt	m1wt	wt

Genes are ordered first by the frequency of mutation in MMR-deficient prostate cancer samples and secondly alphabetically. The three MMR associated genes used as positive controls are depicted in red. MSS stands for microsatellite stable and MSI for microsatellite instable. WT represents the wildtype allele. M1 and M2 correspond to deletion of one or two nucleotides, respectively. P1 and P2 correspond to insertion of one or two nucleotides.

Supplementary Table 5: Mutation profiling of the selected gene panel in all colorectal, endometrial and ovarian cancer cell lines used to assess the mutation status. The MMR proficient cell lines are depicted in blue and the MMR deficient cells lines are in orange. ND represents not defined. WT represents the wildtype. M1, M2 and M3 correspond to deletion of one, two or three nucleotides, respectively. P1 and P2 correspond to insertion of one or two nucleotides.

See Supplementary File 2

Supplementary Table 6: Allele pattern of *PRRT2* and *DAB2IP* in MSI colorectal and endometrial cancer patients

Patient	Type of cancer	PRRT2	DAB2IP
MH09-0412	Colon	wt	wt
MH09-0419	Colon	m1wt	wt
MH09-0421	Colon	wt	wt
MH09-0443	Colon	m1	wt
MH09-0456	Colon	wt	wt
MH09-0462	Colon	m1wt	wt
MH09-0467	Colon	wt	m1wt
MH09-0477	Colon	wt	wtp1
MH09-0483	Colon	m1wt	wt
MH09-0496	Colon	wt	wt
MH09-0503	Colon	wt	wt
MH09-0515	Colon	wtp1	wt
MH09-0541	Colon	wt	wt
MH09-0543	Colon	wt	wt
MH09-0569	Colon	wtp1	m1wt
MH09-0575	Colon	wtp1	wt
MH09-0586	Colon	m1wt	wt
MH09-0587	Colon	m1wt	wt
MH09-0609	Colon	m1wt	wt
MH09-0648	Colon	m1wt	wtp1
MH10-0016	Colon	m1wt	wt
MH10-0017	Colon	m1wt	wtp1
MH10-0030	Colon	m1wt	wt
MH10-0122	Colon	m2m1wt	m1wt
MH07-0071	Uterus	wtp1	wt
MH07-0120	Uterus	m1wt	wt
MH08-0225	Uterus	wt	wt
MH08-0249	Uterus	wt	wt
MH08-0265	Uterus	wt	wt
MH08-0318	Uterus	wt	wt
MH09-0060	Uterus	wtp1	wt
MH09-0071	Uterus	ND	ND
MH09-0095	Uterus	wtp1	wt
MH09-0099	Uterus	wt	wt
MH09-0109	Uterus	wt	wt
MH09-0203	Uterus	wt	ND
MH09-0204	Uterus	wt	ND
MH09-0308	Uterus	m1wt	wt
MH09-0363	Uterus	wt	-
MH09-0393	Uterus	wtp1	wt
MH09-0437	Uterus	m1wtp1	wt
MH09-0440	Uterus	wt	m1wt
MH09-0479	Uterus	m1wt	wtp1
MH09-0486	Uterus	wt	wt
MH09-0535	Uterus	m1wt	wt
MH09-0572	Uterus	m1wt	wt
MH09-0656	Uterus	wt	wt
MH09-0657	Uterus	m1wt	-

ND represents not defined. WT represents the wildtype allele. M1 and M2 correspond to deletion of one or two nucleotides, respectively. P1 corresponds to insertion of one nucleotide.

Supplementary Table 7: Frequency of mutations in MNRs in known MSI sensitive target genes, and in *PRRT2* and *DAB2IP*

Gene	CRC %	EC %	CRCc %
TGFβR2	74.6	14.6	88.6
BAX	41.7	24.0	56.5
Caspase 5	47.6	11.4	84.6
PTEN	18.7	11.6	0.0
MSH6	24.3	15.8	37.0
IGFR2	20.0	16.0	22.7
CEP164	38.1	-	-
PHACTR4	23.8	-	70.0
MLL3	47.6	-	70.0
PRRT2	62.5	45.8	40.0
DAB2IP	25.0	8.3	60.0

CRC is colorectal cancer, EC is endometrial cancer and CRCc is colorectal cancer cell. *PRRT2* and *DAB2IP* are depicted in red. A – means not determined.

Supplementary Table 8: Percentage of cells in different cell cycle phases (< G1, G1, S, G2 and > G2)

		< G1	G1	S	G2	> G2
**HCT116**	GFP	1.5	52.4	25.2	20.9	0
	WT	1.6	50.3	22.5	21.6	4
	MUT	0.1	49.6	31.1	19.2	0
**PNT2C2**	GFP	0.2	53.4	25.8	20.6	0
	WT	1.1	49	22.5	22.6	4.8
	MUT	0.62	41.4	32.2	21.4	4.38

HCT116 and PNT2C2 cells expressing GFP, $PRRT2^{wt}$ and ΔPRRT2 were sampled 48h after plating. ΔPRRT2 expressing cells had less cells in G1 and more cells in S-phase as compared to GFP and $PRRT2^{wt}$ expressing cells.
