# Supplementary material for: A mononucleotide repeat in PRRT2 is an important, frequent target of mismatch repair deficiency in cancer
Source: Oncotarget. 2016 Nov 19;8(4):6043–56. doi: 10.18632/oncotarget.13464 (PMC5351611; doi:10.18632/oncotarget.13464)
Supplement: Supplementary file 2 [file oncotarget-08-6043-s002.docx]

Supplementary Table 1: Gene mutations identified in the PC346C sample (except missense mutations). Type includes SNP, deletions (del), insertions (ins), reference (ref) and no call (-). Impact includes misstart (MS), frameshift (FS), disrupt (D), nonsense (NS) and nonstop (N). Position is defined based on the start of the small variant (SV) call. Additional information on the output from complete genomics can be found on (<http://www.completegenomics.com/FAQs/Variant-Calls-SNPs-and-Small-Indels/>)

| Symbol | Chr# | Position | Type | Ref | Call | Impact | Nuc  POS | Prot  POS | | Ref | | Sample |
| --- | --- | --- | --- | --- | --- | --- | --- | --- | --- | --- | --- | --- |
| AADACL3 | 1 | 12698930 | snp | A | T | MS | 226 | 0 | | M | | L |
| AARS2 | 6 | 44380407 | del | C |  | FS | 1706 | 567 | | Q | | Q |
| ABCA13 | 7 | 48289455 | ins |  | A | FS | 8143 | 2706 | | D | | R |
| ABCA9 | 17 | 64551223 | snp | C | T | D | 942 | 266 | |  | | ATAAAT |
| ABCC5 | 3 | 185147958 | del | A |  | FS | 3425 | 1086 | | P | | P |
| ABO | 9 | 135122729 | ref |  |  | FS | 286 | 87 | | T | | P |
| ABP1 | 7 | 150185058 | del | C |  | FS | 657 | 189 | | R | | G |
| ACACB | 12 | 108089163 | del | G |  | FS | 777 | 256 | | D | | I |
| ACADM | 1 | 75999586 | snp | G | T | NS | 1328 | 379 | | G | | * |
| ACADVL | 17 | 7068424 | - | AGTGGCGAGCTGGTAAGT | ?AGC | D | 1690 | 513 | |  | | ?AGC |
| ACBD3 | 1 | 224440969 | ref | GAGTCGCTCTGCGTTCAGCACCGCCGCCATCTCCGGCTGCTG | GAGTCGCTCTGCGTTCAGCACCGCCGCCATCTCCGGCTGCTG | MS | 47 | 0 | | MAAVLNAERLEVSV | | QQPEMAAVLNEVSV |
| ACIN1 | 14 | 22598353 | del | TC |  | FS | 4194 | 1289 | | E | | E |
| ACSF3 | 16 | 87739911 | snp | C | T | NS | 1836 | 522 | | R | | * |
| ACSM4 | 12 | 7364586 | snp | G | A | D | 921 | 307 | |  | | TTCTCCATCATCCAA |
| ACTR6 | 12 | 99125581 | del | T |  | FS | 321 | 88 | | L | | * |
| ACVR2A | 2 | 148373518 | ins |  | A | FS | 437 | 95 | | D | | R |
| ADAL | 15 | 41428413 | del | A |  | FS | 1070 | 216 | | K | | K |
| ADAM7 | 8 | 24405313 | del | A |  | FS | 1477 | 454 | | K | | K |
| ADAM8 | 10 | 134926670 | ins |  | C | FS | 2505 | 818 | | E | | G |
| ADAMDEC1 | 8 | 24305777 | del | A |  | FS | 193 | 48 | | K | | N |
| ADAMTS8 | 11 | 129786531 | del | G |  | FS | 2097 | 579 | | C | | C |
| ADAMTSL4 | 1 | 148796720 | ins |  | C | FS | 2410 | 724 | | P | | P |
| ADCY8 | 8 | 132121144 | del | G |  | FS | 2710 | 205 | | A | | A |
| ADCY9 | 16 | 4105433 | ref | GGGGAAGCCATGTTGTCGA | GGGGAAGCCATGTTGTCGA | MS | 539 | 0 | | MASPPHQ | | STT* |
| AEBP1 | 7 | 44112976 | del | C |  | FS | 865 | 186 | | P | | P |
| AGAP1 | 2 | 236314420 | del | C |  | FS | 980 | 128 | | P | | R |
| AGK | 7 | 140999065 | del | G |  | FS | 1166 | 380 | | G | | A |
| AHCTF1 | 1 | 245073854 | snp | T | A | D | 6397 | 2130 | |  | | TTCAACTTTTTTTTG |
| AIM2 | 1 | 157299119 | del | T |  | FS | 1262 | 339 | | K | | K |
| AJAP1 | 1 | 4672450 | del | AC |  | FS | 1041 | 220 | | T | | H |
| AKAP10 | 17 | 19821565 | ref | AGGGCCCGGCTCCCCTCATTCAGCAACCGGCCCGGACTTCCG | AGGGCCCGGCTCCCCTCATTCAGCAACCGGCCCGGACTTCCG | MS | 137 | 0 | | MRGAGPSPRQSPRT | | RKSGPVAPRQSPRT |
| AKAP6 | 14 | 32084779 | del | A |  | FS | 1295 | 389 | | P | | P |
| AKAP9 | 7 | 91528534 | del | CAGA |  | FS | 5848 | 1875 | | TE | | S |
| AKR1CL2 | 10 | 4879402 | snp | C | T | NS | 1015 | 300 | | R | | * |
| ALDH3B1 | 11 | 67551956 | ins |  | C | FS | 1344 | 423 | | Q | | P |
| ANKRD11 | 16 | 87874495 | del | CT |  | FS | 6414 | 1984 | | Q | | Q |
| ANLN | 7 | 36426834 | del | A |  | FS | 2323 | 706 | | N | | I |
| ANO10 | 3 | 43622224 | del | T |  | FS | 294 | 41 | | K | | K |
| ANP32D | 12 | 47152974 | snp | T | A | NS | 260 | 86 | | C | | * |
| ANTXR2 | 4 | 81125013 | sub | C | GG | FS | 1607 | 356 | | A | | P |
| ANUBL1 | 10 | 45441411 | del | A |  | FS | 2080 | 621 | | V | | V |
| ANXA13 | 8 | 124775146 | ins |  | T | FS | 721 | 216 | | K | | K |
| AP4M1 | 7 | 99539673 | ins |  | T | FS | 774 | 188 | | L | | F |
| APAF1 | 12 | 97595344 | del | A |  | FS | 2348 | 590 | | N | | T |
| APEX1 | 14 | 19994000 | ins |  | C | FS | 412 | 48 | | P | | P |
| APOB | 2 | 21083519 | ins |  | G | FS | 9853 | 3241 | | L | | L |
| APOBEC3H | 22 | 37827453 | - | AAGTAGGAAA | ?GGT? | D | 484 | 139 | |  | | ?GGT? |
| ARAP3 | 5 | 141013693 | ref | CCTAGGGTCA | CCTAGGGTCA | N | 4710 | 1544 | | * | | R |
| ARHGAP21 | 10 | 24963943 | snp | A | G | D | 794 | 119 | |  | | GCGCTG |
| ARHGAP27 | 17 | 40828581 | ref | CCGCCCCAGTCACAGGCCAGCAGT | CCGCCCCAGTCACAGGCCAGCAGT | N | 2080 | 548 | | * | | W |
| ARHGEF10L | 1 | 17807015 | ins |  | G | FS | 608 | 188 | | V | | G |
| ARPC1A | 7 | 98795214 | snp | G | T | NS | 1047 | 300 | | E | | * |
| ASAH2 | 10 | 51675206 | del | A |  | FS | 83 | 27 | | H | | H |
| ASB11 | X | 15243559 | del | A |  | FS | 88 | 29 | | V | | V |
| ASCL2 | 11 | 2248136 | ref | ATCGCG | ATCGCG | MS | 620 | 0 | | MD | | RD |
| ASF1B | 19 | 14108260 | ref | TTGGCCATCGCCTCGCCTCGCCGCGCCGCAGCAGGGGCAGGGGCTGTG | TTGGCCATCGCCTCGCCTCGCCGCGCCGCAGCAGGGGCAGGGGCTGTG | MS | 172 | 0 | | MAKVSVLNVAVLENPS | | HSPVSVLNVAVLENPS |
| ASH1L | 1 | 153715625 | del | T |  | FS | 4138 | 1219 | | Q | | Q |
| ASMTL | X | 1482163 | del | A |  | FS | 1974 | 621 | | * | | ? |
| ASPSCR1 | 17 | 77547839 | del | C |  | FS | 858 | 253 | | G | | G |
| ASTE1 | 3 | 132215746 | del | T |  | FS | 2325 | 627 | | S | | S |
| ATAD3C | 1 | 1393957 | snp | A | G | N | 373 | 122 | | * | | W |
| ATF3 | 1 | 210859321 | snp | G | T | D | 631 | 116 | |  | | TTGCTTGTCCCCCAT |
| ATG4C | 1 | 63072321 | del | TG |  | FS | 1168 | 319 | | V | | G |
| ATG9B | 7 | 150344836 | ins |  | C | FS | 2370 | 764 | | E | | E |
| ATN1 | 12 | 6915454 | del | C |  | FS | 993 | 254 | | P | | H |
| ATP5E | 20 | 57040679 | ref | CCTGTCTCCAGTAGGCCACCATGCT | CCTGTCTCCAGTAGGCCACCATGCT | MS | 116 | 0 | | MVAYWRQAG | | SMVAYWRPG |
| ATP6V0D2 | 8 | 87234286 | snp | C | T | NS | 1086 | 339 | | R | | * |
| ATP8A1 | 4 | 42353641 | ref | GGGCATCGCGGCGGCGGCTGCAG | GGGCATCGCGGCGGCGGCTGCAG | MS | 232 | 0 | | MPTMRRTV | | LQTMRRTV |
| ATP8B1 | 18 | 53516045 | ins |  | T | FS | 606 | 202 | | K | | K |
| B3GALT2 | 1 | 191416465 | snp | G | A | NS | 1544 | 283 | | R | | * |
| B3GNT6 | 11 | 76429252 | ref | T | T | FS | 1094 | 335 | | L | | L |
| BAI1 | 8 | 143555842 | del | C |  | FS | 1500 | 439 | | Q | | S |
| BAT2L | 9 | 133330265 | del | A |  | FS | 1754 | 566 | | N | | T |
| BAX | 19 | 54150789 | del | G |  | FS | 189 | 40 | | E | | R |
| BBX | 3 | 108912054 | snp | C | T | NS | 377 | 19 | | R | | * |
| BCL2L12 | 19 | 54862130 | snp | C | T | NS | 1084 | 134 | | R | | * |
| BCORL1 | X | 129017697 | del | C |  | FS | 5155 | 1680 | | P | | Q |
| BEGAIN | 14 | 100074042 | ref | GCCTGCACGCAGGCGCTCAGTTG | GCCTGCACGCAGGCGCTCAGTTG | N | 1847 | 591 | | LN* | | LAG |
| BEST4 | 1 | 45022458 | ref | AGGCGAGACCTCAGGGCTCCAGG | AGGCGAGACCTCAGGGCTCCAGG | N | 1458 | 469 | | ALEP* | | AEVSP |
| BNIP1 | 5 | 172511224 | del | T |  | FS | 331 | 75 | | F | | L |
| BOP1 | 8 | 145483792 | del | G |  | FS | 167 | 33 | | P | | P |
| BRD1 | 22 | 48553870 | ref | TGGCGGCCGGGCCGTCA | TGGCGGCCGGGCCGTCA | N | 3661 | 1058 | | * | | P |
| BTNL2 | 6 | 32478948 | del | G |  | FS | 449 | 149 | | I | | I |
| BTNL8 | 5 | 180307126 | del | T |  | FS | 834 | 227 | | F | | S |
| BZW1 | 2 | 201391756 | del | A |  | FS | 930 | 284 | | N | | T |
| C10orf113 | 10 | 21454767 | ins |  | A | FS | 448 | 142 | | T | | T |
| C10orf114 | 10 | 21824529 | ref | AATTTTCAA | AATTTTCAA | N | 855 | 135 | | D* | | EI |
| C10orf88 | 10 | 124687388 | snp | T | A | NS | 1149 | 308 | | K | | * |
| C10orf97 | 10 | 15916607 | ins |  | T | FS | 811 | 196 | | I | | I |
| C11orf40 | 11 | 4549285 | ins |  | CA | FS | 597 | 199 | | C | | C |
| C11orf57 | 11 | 111458783 | del | AAGA |  | FS | 1440 | 253 | | KR | | E |
| C13orf1 | 13 | 49408369 | ref | CATCGCGCA | CATCGCGCA | MS | 105 | 0 | | MAT | | CAT |
| C13orf26 | 13 | 30429008 | snp | G | A | D | 331 | 104 | |  | | TGTTTGCTTTGATAA |
| C14orf105 | 14 | 57018132 | snp | T | C | D | 529 | 130 | |  | | TGTTGTCTTTCATGG |
| C14orf106 | 14 | 44781827 | snp | C | A | D | 793 | 181 | |  | | AAAATAAAACCTTAT |
| C14orf149 | 14 | 59020781 | ref | CTCCATGG | CTCCATGG | MS | 39 | 0 | | MES | | PWS |
| C14orf174 | 14 | 76916508 | snp | C | T | NS | 1719 | 573 | | R | | * |
| C15orf42 | 15 | 87970021 | snp | G | C | D | 5476 | 1825 | |  | | CTGAGT |
| C16orf35 | 16 | 78773 | ins |  | G | FS | 1565 | 488 | | S | | S |
| C16orf5 | 16 | 4503017 | del | G |  | FS | 507 | 96 | | P | | Q |
| C17orf100 | 17 | 6496272 | ins |  | G | FS | 491 | 105 | | A | | G |
| C17orf56 | 17 | 76817283 | ref | AGCAGCTGAAGCTGAAGACTCCAGGGCCAGGCCATCGGGTCAGGCGT | AGCAGCTGAAGCTGAAGACTCCAGGGCCAGGCCATCGGGTCAGGCGT | N | 1592 | 523 | | NA* | | TAA |
| C18orf8 | 18 | 19364459 | snp | C | T | NS | 1900 | 593 | | R | | * |
| C1orf175 | 1 | 54917066 | del | G |  | FS | 2307 | 666 | | G | | G |
| C1orf9 | 1 | 170820850 | snp | T | C | D | 1786 | 522 | |  | | GCACCT |
| C1QL4 | 12 | 48013097 | ref | GCCGGCT | GCCGGCT | N | 1427 | 238 | | * | | C |
| C20orf132 | 20 | 35241207 | ins |  | ATAGACAGGGCCCCGCGGCCGGCACTCTT | FS | 181 | 30 | | K | | KSAGRGALSI |
| C21orf59 | 21 | 32906417 | ref | GAACCATGGCGGTCCCG | GAACCATGGCGGTCCCG | MS | 113 | 0 | | MVLLHV | | RDLLHV |
| C21orf62 | 21 | 33088060 | ins |  | T | FS | 792 | 180 | | L | | L |
| C2CD3 | 11 | 73559439 | del | T |  | FS | 272 | 15 | | K | | K |
| C2orf67 | 2 | 210595931 | del | T |  | FS | 3157 | 983 | | K | | K |
| C2orf83 | 2 | 228184383 | snp | C | T | NS | 509 | 140 | | W | | * |
| C3 | 19 | 6671582 | ref | ACCTGAGGTGGGTCCCATGGT | ACCTGAGGTGGGTCCCATGGT | MS | 62 | 0 | | MGPTSGP | | TMGPTSP |
| C5orf20 | 5 | 134810348 | snp | T | A | NS | 588 | 116 | | R | | * |
| C6 | 5 | 41217322 | del | C |  | FS | 976 | 273 | | Q | | Q |
| C6orf1 | 6 | 34322470 | del | G |  | FS | 869 | 92 | | S | | S |
| C6orf118 | 6 | 165635568 | snp | G | A | NS | 267 | 77 | | Q | | * |
| C6orf132 | 6 | 42218158 | ref | ATGCTGCCGC | ATGCTGCCGC | MS | 533 | 0 | | MKKK | | AKKK |
| C6orf167 | 6 | 97727705 | del | T |  | FS | 3058 | 930 | | G | | G |
| C6orf204 | 6 | 119060444 | snp | C | T | NS | 683 | 31 | | W | | * |
| C7orf57 | 7 | 48058835 | del | A |  | FS | 831 | 206 | | N | | T |
| C9orf102 | 9 | 97678108 | snp | A | G | MS | 388 | 0 | | M | | V |
| C9orf165 | 9 | 34511596 | ins |  | A | FS | 521 | 32 | | A | | V |
| C9orf5 | 9 | 110922008 | ref | GGCCATCG | GGCCATCG | MS | 32 | 0 | | MAD | | RWD |
| C9orf84 | 9 | 113493994 | ins |  | T | FS | 4026 | 1297 | | K | | K |
| CACHD1 | 1 | 64820438 | del | A |  | FS | 225 | 40 | | I | | S |
| CACNA1E | 1 | 179946738 | ins |  | AG | FS | 1246 | 360 | | V | | E |
| CADPS | 3 | 62714468 | del | C |  | FS | 924 | 191 | | R | | L |
| CALML5 | 10 | 5531180 | ref | TTC | TTC | FS | 327 | 72 | | AR | | AK |
| CAMK2N1 | 1 | 20684456 | ref | CATGGTCGC | CATGGTCGC | MS | 856 | 0 | | MSE | | ASE |
| CAMTA1 | 1 | 7646081 | del | G |  | FS | 1094 | 295 | | G | | G |
| CARD8 | 19 | 53429517 | snp | A | T | NS | 342 | 9 | | C | | * |
| CARS2 | 13 | 110091896 | ins |  | T | FS | 1706 | 561 | | S | | I |
| CASP12 | 11 | 104266309 | snp | T | C | D | 1072 | 0 | |  | | GTAAGT |
| CBLB | 3 | 106935595 | sub | TGA | CTAT | FS | 1469 | 382 | | VK | | DR |
| CBX4 | 17 | 75422349 | ref | CGGCTACACCGTCACGTACTCCTTGAAAGTAACGGTGAGGCAGT | CGGCTACACCGTCACGTACTCCTTGAAAGTAACGGTGAGGCAGT | NS | 1805 | 547 | | NCLTVTFKEYVTV* | | SLTVTFKEYVTV* |
| CCDC108 | 2 | 219611501 | snp | T | G | MS | 124 | 0 | | M | | L |
| CCDC144B | 17 | 18438785 | ins |  | A | FS | 1949 | 621 | | I | | I |
| CCDC28A | 6 | 139139029 | del | A |  | FS | 504 | 116 | | N | | M |
| CCDC53 | 12 | 100943940 | ins |  | G | FS | 600 | 147 | | P | | P |
| CCDC67 | 11 | 92743893 | ins |  | A | FS | 688 | 196 | | Q | | T |
| CCDC7 | 10 | 32847439 | snp | G | T | D | 1563 | 331 | |  | | TTTGGA |
| CCDC88A | 2 | 55426871 | del | TT |  | FS | 1730 | 327 | | E | | G |
| CCHCR1 | 6 | 31232827 | snp | C | T | NS | 422 | 77 | | W | | * |
| CCL4L2 | 17 | 31665560 | snp | A | G | D | 267 | 63 | |  | | CTCCTTGTTCTACGG |
| CCNO | 5 | 54565105 | ref | CATGATGC | CATGATGC | MS | 157 | 0 | | MVT | | AVT |
| CCR9 | 3 | 45918071 | del | TT |  | FS | 863 | 250 | | F | | C |
| CCT8L1 | 7 | 151775148 | ins |  | A | FS | 1892 | 551 | | I | | N |
| CD3G | 11 | 117725800 | del | A |  | FS | 249 | 70 | | K | | N |
| CDC27 | 17 | 42589718 | sub | TT | CA | NS | 632 | 168 | | K | | * |
| CDC7 | 1 | 91739952 | del | A |  | FS | 216 | 30 | | N | | T |
| CDCP2 | 1 | 54377907 | ins |  | C | FS | 2071 | 407 | | P | | P |
| CDH23 | 10 | 73112297 | del | C |  | FS | 2338 | 649 | | P | | L |
| CDKN2A | 9 | 21984412 | del | C |  | FS | 77 | 13 | | R | | R |
| CDKN2B | 9 | 21998950 | ref | ATTCCGCAGCCCCCAG | ATTCCGCAGCCCCCAG | MS | 360 | 0 | | MREENK | | LREENK |
| CDKN2C | 1 | 51208676 | del | G |  | FS | 447 | 16 | | D | | T |
| CDR2 | 16 | 22266557 | ins |  | T | FS | 902 | 198 | | K | | K |
| CDSN | 6 | 31191779 | del | CTT |  | FS | 1615 | 529 | | * | | ? |
| CEACAM16 | 19 | 49894552 | del | T |  | FS | 297 | 54 | | L | | * |
| CEBPE | 14 | 22658135 | ref | GACATG | GACATG | MS | 174 | 0 | | MS | | HV |
| CELSR1 | 22 | 45309895 | del | C |  | FS | 1835 | 611 | | L | | L |
| CELSR3 | 3 | 48653758 | snp | A | G | D | 9305 | 3008 | |  | | GCACAG |
| CENPF | 1 | 212885455 | del | A |  | FS | 6093 | 1973 | | T | | P |
| CENPN | 16 | 79613941 | snp | T | G | N | 1402 | 204 | | * | | E |
| CEP120 | 5 | 122741117 | del | T |  | FS | 2288 | 735 | | E | | E |
| CEP164 | 11 | 116727867 | del | A |  | FS | 493 | 115 | | K | | R |
| CEP290 | 12 | 87036445 | ins |  | T | FS | 2000 | 552 | | K | | K |
| CEP350 | 1 | 178331434 | del | A |  | FS | 9045 | 2887 | | N | | I |
| CEP70 | 3 | 139696542 | ref | GTTTT | GTTTT | N | 1991 | 597 | | * | | C |
| CEP76 | 18 | 12689835 | del | T |  | FS | 443 | 95 | | L | | L |
| CHAD | 17 | 45900742 | del | G |  | FS | 483 | 143 | | S | | S |
| CHCHD10 | 22 | 22438188 | ref | CCTCTTC | CCTCTTC | N | 507 | 142 | | * | | W |
| CHD3 | 17 | 7748614 | del | G |  | FS | 4234 | 1341 | | R | | R |
| CHD4 | 12 | 6566968 | del | CCTC |  | FS | 3881 | 1239 | | EG | | E |
| CHD9 | 16 | 51820506 | del | T |  | FS | 2488 | 759 | | F | | L |
| CHST6 | 16 | 74071223 | ref | ACATGCTGA | ACATGCTGA | MS | 180 | 0 | | MWL | | SGL |
| CIB3 | 19 | 16136655 | ins |  | C | FS | 415 | 138 | | G | | G |
| CIT | 12 | 118612615 | del | G |  | FS | 5835 | 1927 | | T | | S |
| CKAP2L | 2 | 113238641 | ref | ACCATGA | ACCATGA | MS | 11 | 0 | | MVG | | SWG |
| CLASP1 | 2 | 121989804 | snp | G | A | NS | 939 | 183 | | R | | * |
| CLCN6 | 1 | 11802155 | ins |  | T | FS | 416 | 101 | | V | | C |
| CLDN14 | 21 | 36755131 | ref | GGCTGTGGGGACTCACACGTA | GGCTGTGGGGACTCACACGTA | N | 1578 | 237 | | YV* | | PTA |
| CLDN16 | 3 | 191588766 | sub | GG | C | FS | 232 | 54 | | RA | | S |
| CLN6 | 15 | 66287511 | ref | CAGAGCCTGGTGCCAGGGACTCAGTGCCGA | CAGAGCCTGGTGCCAGGGACTCAGTGCCGA | N | 1029 | 308 | | SRH* | | SQAL |
| CLPX | 15 | 63237158 | ins |  | T | FS | 1107 | 345 | | N | | K |
| CLTCL1 | 22 | 17569004 | ins |  | C | D | 3675 | 1200 | |  | | CCCTATTATCCGTAGG |
| CNNM2 | 10 | 104669757 | del | C |  | FS | 1654 | 510 | | L | | * |
| CNOT1 | 16 | 57134827 | del | AA |  | FS | 4909 | 1538 | | CF | | C |
| CNRIP1 | 2 | 68400031 | ref | CCCATGTCTGGGCGA | CCCATGTCTGGGCGA | MS | 311 | 0 | | MGDLP | | SPDLP |
| CNTLN | 9 | 17406157 | snp | C | T | NS | 3110 | 1028 | | R | | * |
| CNTNAP4 | 16 | 74869104 | ins |  | T | FS | 429 | 14 | | L | | F |
| COL12A1 | 6 | 75941626 | del | C |  | FS | 2722 | 852 | | G | | G |
| COL13A1 | 10 | 71317213 | del | T |  | FS | 904 | 122 | | F | | F |
| COL18A1 | 21 | 45748867 | del | CCCAGGCCC | FRAMESHIFT | 3398 | 1125 | PRGY | | P | |  |
| COL21A1 | 6 | 56143616 | del | A |  | FS | 1046 | 271 | | V | | V |
| COL25A1 | 4 | 109959911 | del | T |  | FS | 2398 | 622 | | E | | E |
| COL28A1 | 7 | 7537946 | snp | G | A | NS | 354 | 79 | | Q | | * |
| COL7A1 | 3 | 48579566 | ins |  | C | FS | 8108 | 2702 | | R | | R |
| COPG2 | 7 | 129945490 | del | T |  | FS | 1033 | 314 | | A | | E |
| COQ2 | 4 | 84404541 | snp | T | C | D | 997 | 329 | |  | | ATTCCTCTCTTGCGG |
| COTL1 | 16 | 83209014 | ref | TGGCCATCGCCGCGGAGCCGC | TGGCCATCGCCGCGGAGCCGC | MS | 149 | 0 | | MATKIDK | | AAPKIDK |
| COX19 | 7 | 981663 | ref | GTCGACATG | GTCGACATG | MS | 90 | 0 | | MST | | HVD |
| CRAT | 9 | 130904640 | del | C |  | FS | 783 | 162 | | L | | L |
| CRBN | 3 | 3167622 | snp | G | A | NS | 1273 | 418 | | R | | * |
| CREB3L3 | 19 | 4110725 | snp | C | T | NS | 638 | 174 | | R | | * |
| CROT | 7 | 86860076 | snp | T | C | D | 1783 | 532 | |  | | GCAATA |
| CRTAC1 | 10 | 99645732 | snp | C | T | D | 1571 | 405 | |  | | CTGCATTGTTTGTAA |
| CRYBB1 | 22 | 25342211 | del | C |  | FS | 141 | 23 | | K | | K |
| CSMD3 | 8 | 113310210 | del | A |  | FS | 10565 | 3431 | | P | | P |
| CSNK1G2 | 19 | 1931017 | snp | T | C | D | 1716 | 397 | |  | | GCAAGG |
| CST5 | 20 | 23806139 | snp | A | G | D | 411 | 114 | |  | | GCATGT |
| CTSC | 11 | 87710487 | ref | TGCTG | TGCTG | MS | 98 | 0 | | MG | | LG |
| CUBN | 10 | 17153829 | snp | A | G | D | 2497 | 815 | |  | | GCAAGG |
| CXCL14 | 5 | 134942383 | ref | CGGCGTGGGAGCAGGGACATGGGGAGGGCGCTGG | CGGCGTGGGAGCAGGGACATGGGGAGGGCGCTGG | MS | 465 | 0 | | MSLLPRRAPPVS | | PAPSPCPAPPVS |
| CXCL3 | 4 | 75123191 | ref | TGGGGCTCA | TGGGGCTCA | MS | 162 | 0 | | MAH | | LAH |
| CYB561 | 17 | 58868636 | ref | CCATGCTGAGGCAAACGCTGCAAGA | CCATGCTGAGGCAAACGCTGCAAGA | MS | 130 | 0 | | MEGGAAAAT | | R* |
| CYB5D2 | 17 | 3999935 | snp | C | T | NS | 752 | 84 | | R | | * |
| CYFIP2 | 5 | 156654444 | ins |  | C | FS | 538 | 94 | | Q | | P |
| CYHR1 | 8 | 145660474 | del | C |  | FS | 730 | 140 | | W | | W |
| DAB2IP | 9 | 123576464 | del | C |  | FS | 2847 | 900 | | H | | T |
| DAP | 5 | 10734165 | ref | GCTCACTTGCGT | GCTCACTTGCGT | N | 506 | 99 | | PRK* | | PQVS |
| DARS | 2 | 136385419 | snp | A | G | D | 1525 | 447 | |  | | GCAAAC |
| DCLK1 | 13 | 35598262 | ref | GCCGAAGGACATGATGGACTTCAAGTAGG | GCCGAAGGACATGATGGACTTCAAGTAGG | MS | 212 | 0 | | MSFGRDMELE | | PT* |
| DCP1A | 3 | 53299859 | del | TGGCAC |  | D | 1536 | 481 | |  | |  |
| DDX27 | 20 | 47291917 | del | A |  | FS | 2080 | 690 | | K | | R |
| DDX31 | 9 | 134535433 | ref | CTGAGAAGCGAGATCCGGCGCCATCTCACCGA | CTGAGAAGCGAGATCCGGCGCCATCTCACCGA | MS | 152 | 0 | | MAPDLASQRHS | | SVRWRRISRHS |
| DDX59 | 1 | 198860671 | del | T |  | FS | 1874 | 574 | | K | | K |
| DEAF1 | 11 | 643959 | snp | A | G | D | 2285 | 530 | |  | | GCAGGT |
| DEFB126 | 20 | 74313 | del | CC |  | FS | 334 | 105 | | P | | R |
| DENND5B | 12 | 31540009 | snp | C | T | D | 422 | 78 | |  | | ATATGT |
| DGKK | X | 50137887 | ins |  | G | FS | 3119 | 1019 | | G | | G |
| DHDH | 19 | 54137585 | snp | C | T | NS | 736 | 232 | | Q | | * |
| DHH | 12 | 47769905 | ref | CGCTCAGCCCAGTAGCTCCTCC | CGCTCAGCCCAGTAGCTCCTCC | NS | 1479 | 390 | | AEELLG* | | AELLG* |
| DHX15 | 4 | 24195099 | ref | GTGCCGCTTGGACATCCTCGCACTCTTC | GTGCCGCTTGGACATCCTCGCACTCTTC | MS | 168 | 0 | | MSKRHRLDLG | | EECEDRLDLG |
| DHX29 | 5 | 54614785 | snp | A | G | D | 2084 | 654 | |  | | GCAAGC |
| DIAPH1 | 5 | 140978648 | ref | CCGCCGGGCGGCTCCATGTCCCGG | CCGCCGGGCGGCTCCATGTCCCGG | MS | 82 | 0 | | MEPPGGSL | | PGHGAASL |
| DIXDC1 | 11 | 111358318 | ins |  | C | FS | 645 | 60 | | P | | P |
| DKFZp434J1015 | 7 | 6623439 | del | G |  | FS | 619 | 206 | | G | | A |
| DKFZp686O24166 | 11 | 17350614 | del | TT |  | FS | 1860 | 620 | | L | | T |
| DMRTB1 | 1 | 53703036 | del | C |  | FS | 944 | 296 | | P | | R |
| DNAI1 | 9 | 34479448 | snp | T | C | D | 581 | 129 | |  | | GCAGGA |
| DNAJB7 | 22 | 39587067 | del | T |  | FS | 1008 | 292 | | K | | K |
| DNTTIP2 | 1 | 94116003 | del | A |  | FS | 112 | 24 | | S | | S |
| DOCK3 | 3 | 50893034 | snp | G | T | D | 221 | 66 | |  | | TTGAAT |
| DOCK9 | 13 | 98336064 | snp | G | A | NS | 2215 | 720 | | Q | | * |
| DOK3 | 5 | 176863580 | ref | GCTGGGCGTT | GCTGGGCGTT | N | 1490 | 496 | | * | | C |
| DOK6 | 18 | 65557178 | snp | A | C | D | 789 | 199 | |  | | TCTCTATTCTCACCG |
| DRD2 | 11 | 112786640 | ref | TGCGGGCAGGCAGCAGAGTCAGCAGTG | TGCGGGCAGGCAGCAGAGTCAGCAGTG | N | 1488 | 441 | | HC* | | LPA |
| DSC2 | 18 | 26916199 | snp | A | G | D | 1705 | 420 | |  | | GCAAGA |
| DSCAM | 21 | 41140452 | ref | CACATGCCCCTGCCGCTC | CACATGCCCCTGCCGCTC | MS | 452 | 0 | | MWILAL | | ERILAL |
| DSN1 | 20 | 34832866 | del | T |  | FS | 458 | 59 | | K | | K |
| DULLARD | 17 | 7095336 | ref | CATCCCGATG | CATCCCGATG | MS | 446 | 0 | | MMRT | | HMRT |
| DUOX2 | 15 | 43179316 | snp | G | A | NS | 3452 | 1083 | | R | | * |
| DUSP8 | 11 | 1534288 | ref | GGCTGCGGGCGGCGGGGCCGAGGGCAGCGGAGGGGTCAGGACACCTCGATGACCTCC | GGCTGCGGGCGGCGGGGCCGAGGGCAGCGGAGGGGTCAGGACACCTCGATGACCTCC | N | 1984 | 618 | | VEVIEVS* | | VSAPPPAA |
| DYRK1B | 19 | 45008458 | ins |  | G | FS | 1906 | 542 | | P | | P |
| DYRK4 | 12 | 4589628 | del | A |  | FS | 1222 | 354 | | R | | D |
| DZIP1L | 3 | 139264443 | del | CT |  | FS | 2569 | 735 | | Q | | Q |
| E2F6 | 2 | 11523436 | ref | GCCGGCCGCTGCTGACTCATGCTGC | GCCGGCCGCTGCTGACTCATGCTGC | MS | 292 | 0 | | MSQQRPARK | | AA* |
| EAPP | 14 | 34068417 | del | T |  | FS | 475 | 122 | | K | | K |
| ECEL1 | 2 | 233057762 | ins |  | G | FS | 1256 | 350 | | T | | T |
| ECSIT | 19 | 11484959 | del | G |  | FS | 725 | 216 | | R | | G |
| EDEM2 | 20 | 33167167 | sub | T | GC | FS | 1547 | 488 | | Q | | R |
| EDNRB | 13 | 77368576 | ref | ATT | ATT | N | 1542 | 436 | | * | | Y |
| EEA1 | 12 | 91720447 | del | T |  | FS | 2797 | 844 | | K | | K |
| EFR3A | 8 | 133028041 | snp | C | T | NS | 546 | 115 | | Q | | * |
| EFS | 14 | 22904057 | ref | GACGTGGCAATGGCCATGGC | GACGTGGCAATGGCCATGGC | MS | 608 | 0 | | MAIATSV | | AMAIATV |
| EGF | 4 | 111148796 | del | G |  | FS | 3773 | 1110 | | G | | V |
| EGR4 | 2 | 73373949 | ref | GCATGGCGCGGCGCCGGCTGTGGG | GCATGGCGCGGCGCCGGCTGTGGG | MS | 228 | 0 | | MLHLSEFS | | PIHLSEFS |
| EI24 | 11 | 124957513 | ins |  | C | FS | 1090 | 282 | | P | | P |
| EIF3E | 8 | 109297839 | del | T |  | FS | 955 | 309 | | K | | K |
| EIF4G3 | 1 | 21179498 | ins |  | G | FS | 821 | 80 | | P | | P |
| EIF5A2 | 3 | 172107542 | del | T |  | FS | 328 | 66 | | K | | K |
| ELA1 | 12 | 50026654 | sub | CTGGACCATATCCACTTACCATAAAGGAC | ACACCAGGAAGCG | D | 56 | 5 | |  | | CGCTTCCTGGTGT |
| ELAC1 | 18 | 46767370 | del | A |  | FS | 1115 | 336 | | K | | S |
| ELAC2 | 17 | 12861987 | ref | ATGCGCCCGTCTCCACCA | ATGCGCCCGTCTCCACCA | MS | 95 | 0 | | MWALCS | | WWALCS |
| ELFN2 | 22 | 36101517 | ref | CATGGCGCT | CATGGCGCT | MS | 786 | 0 | | MLR | | SLR |
| ELP2 | 18 | 31990573 | del | T |  | FS | 1432 | 474 | | C | | V |
| EMID2 | 7 | 100987391 | ins |  | G | FS | 1373 | 393 | | G | | G |
| EMILIN1 | 2 | 27159329 | ins |  | G | FS | 1386 | 462 | | R | | A |
| EOMES | 3 | 27733761 | del | G |  | FS | 1863 | 621 | | P | | P |
| EPHA7 | 6 | 94185769 | ref | TGAAAAACCATGGTGCATGAGCA | TGAAAAACCATGGTGCATGAGCA | MS | 213 | 0 | | MVFQTRYP | | CSCTTRYP |
| EPPK1 | 8 | 145013370 | del | C |  | FS | 6051 | 1987 | | T | | T |
| EPRS | 1 | 218241112 | ins |  | T | FS | 2441 | 723 | | T | | T |
| ERCC8 | 5 | 60206188 | ref | AAGATGATATTCA | AAGATGATATTCA | N | 1462 | 338 | | * | | L |
| ERF | 19 | 47445211 | del | C |  | FS | 1049 | 297 | | G | | G |
| ERN2 | 16 | 23625601 | del | G |  | FS | 773 | 201 | | T | | T |
| ESSPL | 4 | 152432052 | snp | T | G | N | 984 | 328 | | * | | E |
| ETV1 | 7 | 13995196 | ref | ATCCATGCT | ATCCATGCT | MS | 87 | 0 | | MDG | | SMG |
| EXOC3L | 16 | 65777731 | snp | G | A | NS | 1645 | 468 | | R | | * |
| EXOSC10 | 1 | 11056922 | ins |  | T | FS | 2084 | 678 | | K | | K |
| FAM101B | 17 | 295952 | ref | TGGACTC | TGGACTC | FS | 273 | 69 | | PSP | | ? |
| FAM131B | 7 | 142763999 | ins |  | C | FS | 2470 | 254 | | G | | G |
| FAM155B | X | 68642629 | del | A |  | FS | 827 | 259 | | K | | N |
| FAM160B2 | 8 | 22011999 | ins |  | C | FS | 940 | 296 | | L | | P |
| FAM173B | 5 | 10292377 | del | C |  | FS | 135 | 35 | | W | | W |
| FAM176B | 1 | 36561215 | ref | GGGCATCCATGCTGCTCTGGGGGGCAGC | GGGCATCCATGCTGCTCTGGGGGGCAGC | MS | 292 | 0 | | MDAPRRDMEL | | AAPPRRDMEL |
| FAM187B | 19 | 40410859 | snp | C | T | NS | 608 | 187 | | W | | * |
| FAM83G | 17 | 18848075 | ref | CCATGGCGCCGCCTGCCCGGGCACT | CCATGGCGCCGCCTGCCCGGGCACT | MS | 167 | 0 | | MAFSQVQCL | | SAFSQVQCL |
| FAM92A2 | 15 | 39243360 | del | TTAG |  | FS | 471 | 157 | | LE | | K |
| FANCA | 16 | 88386456 | snp | C | A | D | 1047 | 335 | |  | | TTTCTCCCCCTGCAT |
| FANCD2 | 3 | 10053017 | del | T |  | FS | 563 | 161 | | F | | L |
| FANCE | 6 | 35533698 | del | C |  | FS | 1113 | 309 | | P | | Q |
| FANK1 | 10 | 127687965 | del | A |  | FS | 1110 | 335 | | K | | S |
| FARP2 | 2 | 241991913 | snp | A | G | D | 300 | 61 | |  | | TCTCTCTCTGCACGG |
| FARSB | 2 | 223215838 | snp | G | A | NS | 278 | 81 | | R | | * |
| FASN | 17 | 77630306 | ref | CCT | CCT | N | 7651 | 2511 | | * | | W |
| FBN1 | 15 | 46589553 | snp | G | A | NS | 2020 | 564 | | R | | * |
| FBN3 | 19 | 8102154 | del | A |  | FS | 2028 | 669 | | C | | A |
| FBXL13 | 7 | 102311357 | del | T |  | FS | 1544 | 372 | | E | | E |
| FBXL18 | 7 | 5487922 | ref | CTCCGCCTCT | CTCCGCCTCT | N | 2250 | 718 | | * | | W |
| FBXO17 | 19 | 44132797 | ref | ATCT | ATCT | MS | 172 | 0 | | MG | | RG |
| FBXO5 | 6 | 153345768 | ref | TGCAGGGGCGCCGGCTCATGCCAGCCGACGTGG | TGCAGGGGCGCCGGCTCATGCCAGCCGACGTGG | MS | 81 | 0 | | MSRRPCSCALR | | PRRLA* |
| FDXR | 17 | 70370473 | ref | CAGCCCTTCCCCTCCCAACACTCATCCCTTCCCTGCTGGGGGCCGGGGCTGGGGCTGGGCTC | CAGCCCTTCCCCTCCCAACACTCATCCCTTCCCTGCTGGGGGCCGGGGCTGGGGCTGGGCTC | N | 1561 | 491 | | * | | L |
| FHAD1 | 1 | 15446290 | ins |  | C | FS | 20 | 6 | | P | | P |
| FHOD1 | 16 | 65838805 | ref | GCCCGCCATGGCTCTGCGGCCGGCTCA | GCCCGCCATGGCTCTGCGGCCGGCTCA | MS | 112 | 0 | | MAGGEDRGD | | * |
| FICD | 12 | 107436932 | del | C |  | FS | 1073 | 309 | | R | | G |
| FIP1L1 | 4 | 54014018 | ins |  | AG | FS | 1603 | 486 | | R | | RG |
| FLJ20433 | 9 | 139387288 | del | G |  | FS | 546 | 116 | | S | | S |
| FLJ21511 | 4 | 48704053 | ins |  | A | FS | 1321 | 379 | | N | | K |
| FLJ21865 | 17 | 74585542 | snp | T | C | D | 424 | 138 | |  | | GCGAGG |
| FLJ22167 | 16 | 74136749 | snp | C | T | D | 644 | 193 | |  | | ATAAGA |
| FLJ33534 | 2 | 11181894 | snp | A | G | D | 1014 | 0 | |  | | GCGAAC |
| FLJ34208 | 3 | 195688279 | ins |  | C | FS | 90 | 30 | | R | | P |
| FLJ35429 | 6 | 29819836 | snp | C | A | D | 1124 | 0 | |  | | TGTCATTATTATTAT |
| FLJ35776 | 18 | 3587159 | del | CTGT |  | FS | 775 | 106 | | VC | | V |
| FLJ39080 | 8 | 75827249 | del | A |  | FS | 305 | 82 | | H | | P |
| FLJ41047 | 11 | 125212970 | snp | A | C | D | 96 | 16 | |  | | CTCTCCACCCTGCCG |
| FLJ42177 | 6 | 109956914 | del | T |  | FS | 1108 | 287 | | R | | G |
| FLJ45139 | 21 | 39172632 | del | A |  | FS | 2273 | 25 | | S | | S |
| FLJ45983 | 10 | 8133206 | ref | CCATTTCTCACCTTTT | CCATTTCTCACCTTTT | MS | 1421 | 0 | | MEPDFL | | KKPDFL |
| FLT3LG | 19 | 54673986 | ins |  | C | FS | 441 | 117 | | S | | Q |
| FMN2 | 1 | 238559301 | snp | C | T | NS | 4776 | 1592 | | R | | * |
| FMNL1 | 17 | 40674559 | ref | G | G | FS | 1560 | 453 | | G | | G |
| FNDC5 | 1 | 33102871 | del | C |  | FS | 612 | 129 | | G | | G |
| FOXA2 | 20 | 22512895 | ref | CATACTGGAAGCCGAGTGCA | CATACTGGAAGCCGAGTGCA | MS | 191 | 0 | | MLGAVKM | | YLGAVKM |
| FOXH1 | 8 | 145671919 | ref | CCAGGCGGGAGCCGCTGCAGGGCCCCATGCGGGACGGTAGACAGCGTGGGCAG | CCAGGCGGGAGCCGCTGCAGGGCCCCATGCGGGACGGTAGACAGCGTGGGCAG | MS | 579 | 0 | | MGPCSGSRLGPPEAESPS | | LPTLSTVPHGPPEAESPS |
| FOXS1 | 20 | 29896990 | ref | GAGGCTGCTGCTGCATGCTGGCCG | GAGGCTGCTGCTGCATGCTGGCCG | MS | 75 | 0 | | MQQQPLPG | | RPACSMPG |
| FRAS1 | 4 | 79405217 | snp | C | T | NS | 686 | 206 | | R | | * |
| FRAT2 | 10 | 99083616 | ref | GT | GT | N | 830 | 233 | | * | | C |
| FRMD4A | 10 | 14412106 | ref | TGGTC | TGGTC | MS | 352 | 0 | | MA | | VA |
| FRYL | 4 | 48206931 | snp | C | A | D | 8899 | 2764 | |  | | CTTCTGATTTGACAT |
| FSD2 | 15 | 81234769 | snp | C | T | NS | 1635 | 489 | | W | | * |
| FTO | 16 | 52295643 | snp | T | C | D | 267 | 15 | |  | | GCATGT |
| FUT10 | 8 | 33366492 | snp | G | A | NS | 1097 | 247 | | R | | * |
| FXR1 | 3 | 182148928 | del | A |  | FS | 736 | 38 | | N | | I |
| FZD4 | 11 | 86339891 | del | CT |  | FS | 1858 | 517 | | K | | K |
| FZD5 | 2 | 208341705 | ref | CATCGCCC | CATCGCCC | MS | 410 | 0 | | MAR | | GAR |
| GABBR1 | 6 | 29685128 | del | G |  | FS | 1948 | 571 | | S | | S |
| GABRD | 1 | 1951462 | del | G |  | FS | 1259 | 413 | | G | | A |
| GALNTL5 | 7 | 151330835 | del | C |  | FS | 985 | 254 | | L | | * |
| GARNL1 | 14 | 35134705 | del | T |  | FS | 5966 | 1858 | | Q | | Q |
| GART | 21 | 33804000 | del | T |  | FS | 2497 | 803 | | E | | E |
| GBGT1 | 9 | 135019465 | snp | G | T | NS | 643 | 120 | | Y | | * |
| GDI2 | 10 | 5878797 | del | G |  | FS | 338 | 62 | | P | | P |
| GDPD4 | 11 | 76632437 | ins |  | A | FS | 1441 | 396 | | N | | N |
| GFM1 | 3 | 159890767 | snp | C | T | NS | 2138 | 677 | | Q | | * |
| GFOD1 | 6 | 13473865 | - | GCCGATGCCTGCGGGTGGGAGGAAGACAGCGGTCAGCGG | ?GAGA? | D | 917 | 84 | |  | | ?TCTC? |
| GGH | 8 | 64113863 | ref | GCAGCCCGGACTGGCCATGGCG | GCAGCCCGGACTGGCCATGGCG | MS | 59 | 0 | | MASPGCLL | | RHGQSGLL |
| GHR | 5 | 42730837 | del | A |  | FS | 371 | 109 | | N | | T |
| GIT2 | 12 | 108918366 | del | C |  | FS | 116 | 15 | | G | | G |
| GLG1 | 16 | 73198474 | ref | CACGTCCACACGCCGCCATCTTGAGTCCGCGGCGAG | CACGTCCACACGCCGCCATCTTGAGTCCGCGGCGAG | MS | 25 | 0 | | MAACGRVRRMFR | | LAADSRLRRMFR |
| GLIS1 | 1 | 53832667 | del | G |  | FS | 1061 | 165 | | P | | P |
| GMIP | 19 | 19615335 | ref | CATAT | CATAT | MS | 117 | 0 | | MD | | ID |
| GNPAT | 1 | 229468413 | del | T |  | FS | 946 | 267 | | F | | L |
| GNRH2 | 20 | 2974363 | ins |  | CCGCC | FS | 371 | 106 | | P | | PRH |
| GOLSYN | 8 | 110724130 | snp | A | G | D | 741 | 75 | |  | | GCACAG |
| GORASP1 | 3 | 39124030 | ref | GCCCATGGCAGCGGCTCC | GCCCATGGCAGCGGCTCC | MS | 98 | 0 | | MGLGVS | | GALGVS |
| GOT1L1 | 8 | 37913463 | ins |  | T | FS | 794 | 231 | | Q | | Q |
| GP6 | 19 | 60235684 | snp | C | T | D | 94 | 22 | |  | | ATGAGT |
| GPATCH4 | 1 | 154831674 | ins |  | CA | FS | 1195 | 355 | | T | | TV |
| GPBAR1 | 2 | 218835898 | ins |  | C | FS | 567 | 69 | | T | | H |
| GPHB5 | 14 | 62854161 | ins |  | G | FS | 155 | 51 | | G | | G |
| GPR113 | 2 | 26422596 | ref | GGGTCGTCATGGCTGGCTACGAATACG | GGGTCGTCATGGCTGGCTACGAATACG | MS | 583 | 0 | | MTTRKLSAH | | RIRRKLSAH |
| GPR171 | 3 | 152399806 | del | A |  | FS | 296 | 18 | | Y | | Y |
| GPR175 | 3 | 128778148 | snp | C | T | D | 788 | 165 | |  | | ATAAGA |
| GPR37 | 7 | 124174224 | snp | G | A | NS | 2082 | 477 | | R | | * |
| GPR75 | 2 | 53934493 | snp | G | A | NS | 1113 | 301 | | R | | * |
| GREB1 | 2 | 11644957 | snp | G | A | D | 1475 | 386 | |  | | TCTTGCCCACTGCAA |
| GREM2 | 1 | 238722888 | ref | CGCTC | CGCTC | N | 785 | 168 | | * | | S |
| GRHL3 | 1 | 24535736 | del | CCTG |  | FS | 318 | 55 | | PA | | Q |
| GRID2 | 4 | 94595994 | del | CT |  | FS | 1962 | 568 | | L | | M |
| GRIN2B | 12 | 13910124 | del | C |  | FS | 493 | 94 | | Q | | Q |
| GRIP2 | 3 | 14536633 | ins |  | G | FS | 1308 | 436 | | P | | P |
| GSTM3 | 1 | 110084431 | ref | TGG | TGG | MS | 131 | 0 | | M | | L |
| GTF3C6 | 6 | 111386670 | ins |  | C | FS | 215 | 1 | | A | | A |
| GTSE1 | 22 | 45090782 | del | C |  | FS | 1279 | 394 | | P | | P |
| HAND1 | 5 | 153837760 | ref | TGTTGGAGCGGCTACTGGCCTG | TGTTGGAGCGGCTACTGGCCTG | MS | 256 | 0 | | MNLVGSYA | | LNLVGSYA |
| HCFC2 | 12 | 103000671 | del | A |  | FS | 937 | 308 | | N | | I |
| HDAC4 | 2 | 239667765 | del | G |  | FS | 3488 | 898 | | D | | D |
| HDGF2 | 19 | 4442818 | del | G |  | FS | 757 | 221 | | G | | D |
| HEL308 | 4 | 84593612 | del | T |  | FS | 985 | 268 | | L | | L |
| HELLS | 10 | 96296225 | del | AG |  | FS | 238 | 44 | | E | | A |
| HES2 | 1 | 6401513 | ref | GAGGGGC | GAGGGGC | N | 620 | 173 | | * | | Y |
| HGFAC | 4 | 3417630 | sub | G | CC | FS | 1169 | 388 | | P | | PR |
| HIBCH | 2 | 190892719 | snp | A | G | MS | 84 | 0 | | M | | T |
| HIPK3 | 11 | 33325537 | snp | T | C | D | 2289 | 673 | |  | | GCTGGT |
| HKR1 | 19 | 42545458 | snp | C | T | NS | 1190 | 307 | | R | | * |
| HLA-DPB1 | 6 | 33156670 | sub | CA | G | FS | 403 | 114 | | PM | | P |
| HLA-DPB2 | 6 | 33204500 | snp | T | G | D | 756 | 0 | |  | | GGGAGA |
| HLA-DRB1 | 6 | 32659929 | ref | CCCGC | CCCGC | FS | 361 | 99 | | RRA | | SAG |
| HMG20B | 19 | 3526563 | del | GA |  | FS | 445 | 125 | | EK | | E |
| HNF1A | 12 | 119916499 | del | G |  | FS | 886 | 287 | | G | | G |
| HNF1B | 17 | 33178969 | ref | ACGTGAGCTTGGACACCATTTTCCA | ACGTGAGCTTGGACACCATTTTCCA | MS | 221 | 0 | | MVSKLTSLQ | | WKMVSKPLQ |
| HOXB8 | 17 | 44047061 | ref | TCATTTTAT | TCATTTTAT | MS | 235 | 0 | | MSS | | ISS |
| HSPB6 | 19 | 40939747 | ref | ATCCTGC | ATCCTGC | MS | 21 | 0 | | MEI | | AEI |
| HTR4 | 5 | 147869471 | sub | CC | A | FS | 978 | 271 | | W | | L |
| HYDIN | 16 | 69618995 | snp | A | G | N | 3174 | 1017 | | * | | Q |
| IFNA10 | 9 | 21197036 | snp | A | T | NS | 105 | 19 | | C | | * |
| IFNA17 | 9 | 21218003 | ins |  | T | FS | 218 | 56 | | H | | Q |
| IGFBP1 | 7 | 45898064 | snp | C | T | NS | 821 | 176 | | R | | * |
| IL17RB | 3 | 53864393 | del | A |  | FS | 553 | 171 | | K | | S |
| IL17REL | 22 | 48781365 | snp | G | A | NS | 392 | 54 | | Q | | * |
| IL34 | 16 | 69251500 | snp | C | A | NS | 1021 | 212 | | Y | | * |
| INO80B | 2 | 74536099 | ins |  | A | FS | 162 | 39 | | H | | T |
| INO80E | 16 | 29924161 | del | C |  | FS | 733 | 210 | | P | | P |
| INPP5D | 2 | 233777595 | del | C |  | FS | 3213 | 1020 | | L | | C |
| INPP5F | 10 | 121541418 | snp | G | T | NS | 658 | 164 | | E | | * |
| INPPL1 | 11 | 71619705 | del | A |  | FS | 1526 | 440 | | N | | T |
| IPO11 | 5 | 61882878 | del | T |  | FS | 2587 | 806 | | S | | L |
| ISY1 | 3 | 130362498 | ref | TACTCACCATGGTGTCGCTAAGGGCGC | TACTCACCATGGTGTCGCTAAGGGCGC | MS | 211 | 0 | | MARNAEKAM | | AARNAEKAM |
| ITGAE | 17 | 3603420 | ins |  | T | FS | 1679 | 526 | | G | | G |
| ITIH5 | 10 | 7645083 | del | C |  | FS | 2875 | 932 | | D | | T |
| ITPA | 20 | 3141859 | snp | C | T | NS | 202 | 20 | | Q | | * |
| ITPR2 | 12 | 26726073 | ins |  | TCAC | FS | 1826 | 469 | | R | | R* |
| JAK1 | 1 | 65079591 | del | T |  | FS | 2821 | 857 | | E | | E |
| JRK | 8 | 143743095 | del | CA |  | FS | 1896 | 460 | | VV | | V |
| KANK2 | 19 | 11165259 | del | G |  | FS | 819 | 165 | | P | | P |
| KCNH3 | 12 | 48234590 | del | G |  | FS | 2382 | 707 | | G | | E |
| KCNH4 | 17 | 37586483 | ref | CGGCATGGCCCCGGGG | CGGCATGGCCCCGGGG | MS | 333 | 0 | | MPVMKG | | PRVMKG |
| KCNJ10 | 1 | 158278646 | del | G |  | FS | 449 | 99 | | D | | D |
| KCNMA1 | 10 | 78399799 | del | T |  | FS | 2300 | 707 | | P | | P |
| KCNMB3 | 3 | 180443463 | del | T |  | FS | 1095 | 251 | | G | | G |
| KCNN4 | 19 | 48976852 | ref | TGGCCCCCGGG | TGGCCCCCGGG | MS | 396 | 0 | | MGGD | | LGGD |
| KCTD12 | 13 | 76357298 | ref | TGGGGAGCTCACTCCCTGCAGAAGACGTACTCGGTGTAGCT | TGGGGAGCTCACTCCCTGCAGAAGACGTACTCGGTGTAGCT | N | 1202 | 315 | | SYTEYVFCRE* | | RVRLLQGVSSP |
| KIAA0133 | 1 | 227856716 | del | T |  | FS | 4471 | 1445 | | S | | P |
| KIAA0256 | 15 | 47092193 | del | T |  | FS | 1672 | 512 | | A | | A |
| KIAA0329 | 14 | 101950833 | del | T |  | FS | 736 | 196 | | Y | | T |
| KIAA0528 | 12 | 22526931 | del | T |  | FS | 1790 | 520 | | L | | L |
| KIAA0564 | 13 | 41191721 | ref | CTTACTCCTTT | CTTACTCCTTT | N | 3180 | 1036 | | AKE* | | ARSK |
| KIAA0644 | 7 | 28964186 | ref | TCGC | TCGC | MS | 367 | 0 | | ME | | VE |
| KIAA0664 | 17 | 2550809 | del | G |  | FS | 1056 | 323 | | A | | A |
| KIAA1211 | 4 | 56874266 | ins |  | A | FS | 892 | 167 | | Q | | T |
| KIAA1383 | 1 | 231008523 | del | G |  | FS | 1263 | 377 | | E | | S |
| KIAA1543 | 19 | 7581773 | del | C |  | FS | 1106 | 335 | | R | | G |
| KIAA1602 | 12 | 48478469 | del | G |  | FS | 596 | 131 | | S | | S |
| KIAA1751 | 1 | 1876878 | snp | A | G | N | 2442 | 762 | | * | | Q |
| KIAA1797 | 9 | 20811018 | snp | G | A | NS | 2104 | 580 | | W | | * |
| KIAA1908 | 7 | 1594157 | snp | T | C | N | 1479 | 264 | | * | | R |
| KIAA1919 | 6 | 111694061 | del | T |  | FS | 1025 | 201 | | C | | V |
| KIAA2018 | 3 | 114860181 | del | T |  | FS | 3447 | 1012 | | K | | K |
| KIF18A | 11 | 28066834 | snp | G | A | NS | 890 | 236 | | R | | * |
| KIF22 | 16 | 29721705 | snp | C | T | NS | 1419 | 465 | | R | | * |
| KIF26A | 14 | 103712168 | ins |  | G | FS | 3290 | 1096 | | E | | E |
| KIFC3 | 16 | 56389653 | ref | CATGGCCTGGGGCTCA | CATGGCCTGGGGCTCA | MS | 197 | 0 | | MVPSRR | | * |
| KLC1 | 14 | 103193841 | del | A |  | FS | 775 | 155 | | K | | N |
| KLHL11 | 17 | 37264808 | ins |  | CT | FS | 842 | 278 | | E | | EE |
| KLK12 | 19 | 56226941 | snp | A | G | D | 573 | 152 | |  | | GCAAGG |
| KRT24 | 17 | 36111660 | del | A |  | FS | 722 | 221 | | N | | K |
| KRT76 | 12 | 51453682 | snp | C | A | NS | 855 | 275 | | E | | * |
| KRT9 | 17 | 36977490 | snp | G | A | NS | 1497 | 477 | | R | | * |
| KRTAP10-6 | 21 | 44835685 | ref | TCCAGGGCTGTCAGCAGCTGGA | TCCAGGGCTGTCAGCAGCTGGA | N | 1107 | 362 | | SSC* | | DSPG |
| KRTAP1-1 | 17 | 36451169 | ref | GGCCATGGTGTCAGGAGTTGATCTGAAGGTTGGGT | GGCCATGGTGTCAGGAGTTGATCTGAAGGTTGGGT | MS | 64 | 0 | | MACCQTSFCGFP | | TQCCQTSFCGFP |
| KRTAP12-4 | 21 | 44898613 | ref | CACCTGCTC | CACCTGCTC | N | 382 | 112 | | * | | L |
| KRTAP13-2 | 21 | 30665997 | snp | A | T | NS | 430 | 134 | | C | | * |
| KRTAP19-6 | 21 | 30835852 | del | G |  | FS | 199 | 56 | | F | | F |
| KRTAP2-1 | 17 | 36457042 | ref | CATG | CATG | MS | 58 | 0 | | MT | | HT |
| KRTAP4-2 | 17 | 36587533 | snp | A | G | N | 452 | 136 | | * | | R |
| KRTAP5-3 | 11 | 1586179 | ref | AGAGCAGCCCATG | AGAGCAGCCCATG | MS | 78 | 0 | | MGCSG | | HGLLG |
| LAMA2 | 6 | 129615085 | del | AG |  | FS | 2153 | 682 | | RV | | S |
| LAMP3 | 3 | 184363130 | ref | GGGGCATGGTGGGCGCTGGGCGAGGTTC | GGGGCATGGTGGGCGCTGGGCGAGGTTC | MS | 224 | 0 | | MPRQLSAAAA | | EPRQLSAAAA |
| LCAP | X | 152802902 | ins |  | C | FS | 539 | 11 | | W | | L |
| LEPREL2 | 12 | 6808285 | ins |  | G | FS | 454 | 140 | | L | | A |
| LIG4 | 13 | 107661610 | ref | CAGCCATCAAAG | CAGCCATCAAAG | MS | 235 | 0 | | MAAS | | L* |
| LILRB4 | 19 | 59869686 | snp | G | T | NS | 1337 | 325 | | G | | * |
| LMAN2 | 5 | 176691839 | del | G |  | FS | 923 | 307 | | D | | D |
| LMBRD2 | 5 | 36176975 | del | A |  | FS | 772 | 119 | | F | | F |
| LMOD2 | 7 | 123090318 | del | A |  | FS | 1599 | 480 | | K | | K |
| LOC100127933 | 14 | 105024071 | ins |  | G | FS | 373 | 45 | | Q | | A |
| LOC100127987 | 18 | 75418063 | sub | GCTG | TGCGGC | FS | 2203 | 734 | | PA | | RRK |
| LOC100128055 | 4 | 144654367 | ref | GGCTCAGGCCCGTCGCGGAGGCGCGGCGC | GGCTCAGGCCCGTCGCGGAGGCGCGGCGC | NS | 623 | 109 | | CAAPPRRA* | | SAPPRRA* |
| LOC100128076 | 9 | 93936046 | ins |  | CCAC | FS | 133 | 44 | | Q | | PT |
| LOC100128208 | 7 | 32823414 | ins |  | G | FS | 437 | 145 | | G | | G |
| LOC100128216 | 5 | 1518239 | del | CACA |  | FS | 338 | 112 | | FV | | F |
| LOC100128382 | 5 | 8893741 | ref | ACCATA | ACCATA | MS | 1826 | 0 | | MV | | YG |
| LOC100128406 | 2 | 132806606 | snp | C | T | NS | 188 | 62 | | W | | * |
| LOC100128499 | 6 | 4024732 | del | CTCCTCGGGC | FRAMESHIFT | 703 | 167 | KPEE | | K | |  |
| LOC100128528 | 19 | 54695632 | ins |  | GA | FS | 40 | 13 | | T | | R |
| LOC100128545 | 8 | 61488380 | snp | A | G | N | 351 | 117 | | * | | R |
| LOC100128610 | 6 | 63997562 | ins |  | G | FS | 728 | 137 | | L | | L |
| LOC100128619 | 5 | 172504583 | ref | GCCCCGCCCTCTGACGCTGCTTCAGCATCCT | GCCCCGCCCTCTGACGCTGCTTCAGCATCCT | MS | 37 | 0 | | MLKQRQRAGPS | | RMLKQRQRAAS |
| LOC100128648 | 6 | 13473865 | - | GCCGATGCCTGCGGGTGGGAGGAAGACAGCGGTCAGCGG | ?GAGA? | D | 959 | 319 | |  | | ?GAGA? |
| LOC100128714 | 15 | 23811724 | del | C |  | FS | 352 | 111 | | P | | R |
| LOC100128718 | 19 | 17377627 | snp | G | A | D | 5 | 1 | |  | | TTGGGCCCTTCCCAA |
| LOC100128751 | 1 | 165906233 | del | A |  | FS | 1749 | 21 | | K | | N |
| LOC100128781 | 14 | 87480972 | snp | G | T | D | 52 | 0 | |  | | TTATGT |
| LOC100128792 | 11 | 118328907 | del | AA |  | FS | 454 | 92 | | PF | | P |
| LOC100128829 | 8 | 73127596 | snp | T | C | N | 317 | 96 | | * | | Q |
| LOC100128877 | 4 | 81336933 | ins |  | A | FS | 1725 | 211 | | T | | N |
| LOC100128892 | 3 | 113849658 | ins |  | C | FS | 85 | 28 | | R | | R |
| LOC100128901 | 5 | 177032008 | sub | G | AA | FS | 476 | 158 | | F | | FF |
| LOC100128908 | 14 | 22643968 | ref | TCTC | TCTC | MS | 42 | 0 | | ML | | VL |
| LOC100129010 | 5 | 92949518 | ref | CGCTACGGC | CGCTACGGC | N | 431 | 96 | | LP* | | LVA |
| LOC100129026 | 5 | 172132094 | snp | G | A | NS | 1745 | 372 | | W | | * |
| LOC100129042 | 1 | 15198483 | snp | G | A | NS | 371 | 20 | | W | | * |
| LOC100129058 | 3 | 51883265 | snp | T | C | N | 531 | 117 | | * | | Q |
| LOC100129068 | 11 | 125786267 | del | T |  | FS | 390 | 130 | | * | | ? |
| LOC100129103 | 10 | 131466408 | ins |  | GGCGGCAGGATTTCAGAAGG | FS | 1772 | 590 | | V | | VAAGFQKD |
| LOC100129147 | 6 | 150288769 | del | C |  | FS | 2412 | 83 | | T | | T |
| LOC100129149 | 2 | 37751617 | del | A |  | FS | 0 | 0 | | M | | C |
| LOC100129194 | 3 | 32254857 | snp | T | C | N | 1679 | 172 | | * | | Q |
| LOC100129200 | 1 | 33131451 | snp | C | T | NS | 284 | 94 | | W | | * |
| LOC100129215 | 16 | 86286835 | ins |  | A | FS | 413 | 137 | | G | | G |
| LOC100129240 | 4 | 186145939 | ins |  | TG | FS | 330 | 94 | | G | | GT |
| LOC100129444 | 12 | 119633131 | del | CGCTAGGA |  | FS | 83 | 20 | | | ILA | M |
| LOC100129465 | 10 | 1805308 | ref | CCGCCCCAGGACTGCCACGTGACCATC | CCGCCCCAGGACTGCCACGTGACCATC | MS | 237 | 0 | | MVTWQSWGG | | DGHVAVLGR |
| LOC100129498 | 11 | 117538185 | del | TA |  | FS | 256 | 24 | | L | | L |
| LOC100129502 | 15 | 88242600 | snp | T | C | MS | 692 | 0 | | M | | V |
| LOC100129550 | 3 | 124088663 | del | GA |  | FS | 616 | 52 | | R | | K |
| LOC100129551 | 4 | 144695801 | ins |  | T | FS | 2404 | 235 | | V | | V |
| LOC100129827 | 11 | 10519631 | snp | A | T | NS | 276 | 92 | | R | | * |
| LOC100129835 | 15 | 41210594 | ref | TAGC | TAGC | MS | 142 | 0 | | MA | | VA |
| LOC100129876 | X | 55215188 | ins |  | T | FS | 77 | 25 | | L | | F |
| LOC100129969 | 9 | 34883045 | sub | CC | G | FS | 409 | 117 | | LA | | L |
| LOC100129979 | 15 | 30561050 | del | A |  | FS | 170 | 56 | | E | | E |
| LOC100130030 | 21 | 37660982 | ref | TTTTTAAACCCTTTCGCTTCCCGCCCGAATAATAATAA | TTTTTAAACCCTTTCGCTTCCCGCCCGAATAATAATAA | NS | 3759 | 159 | | FIIIRAGSERV* | | FIIRAGSERV* |
| LOC100130107 | 15 | 57847773 | snp | C | T | NS | 934 | 254 | | W | | * |
| LOC100130135 | 3 | 45610909 | ref | CCGATTCAGCTTCACCCG | CCGATTCAGCTTCACCCG | N | 581 | 193 | | AG* | | ANR |
| LOC100130193 | 1 | 19600482 | snp | G | A | NS | 390 | 130 | | R | | * |
| LOC100130226 | 20 | 61675566 | del | G |  | FS | 360 | 120 | | P | | P |
| LOC100130229 | 2 | 196744190 | ref | ACCGCCT | ACCGCCT | N | 526 | 175 | | * | | C |
| LOC100130230 | 5 | 176988799 | del | T |  | FS | 1287 | 296 | | S | | S |
| LOC100130297 | 18 | 54901992 | snp | T | C | D | 106 | 35 | |  | | GCGCGT |
| LOC100130302 | X | 24296977 | snp | T | A | D | 2426 | 808 | |  | | GACAGT |
| LOC100130315 | 2 | 133592238 | del | T |  | FS | 312 | 104 | | Y | | T |
| LOC100130390 | 16 | 48736024 | snp | G | A | NS | 208 | 20 | | R | | * |
| LOC100130430 | 16 | 1298633 | del | GGAGGGAGGGAATTAGTGAG |  | FS | 762 | 123 | TH* | | | ? |
| LOC100130460 | 11 | 10388945 | snp | A | C | N | 567 | 86 | | * | | C |
| LOC100130536 | 6 | 32229323 | snp | A | C | N | 263 | 80 | | * | | G |
| LOC100130581 | 17 | 38821646 | ref | CGGCGCATGGA | CGGCGCATGGA | MS | 441 | 0 | | MRRL | | SMRL |
| LOC100130587 | 20 | 61468344 | snp | T | G | N | 435 | 115 | | * | | G |
| LOC100130609 | 7 | 138727856 | snp | T | A | NS | 238 | 79 | | L | | * |
| LOC100130656 | 19 | 14356433 | del | GTTCCCCTGCC |  | FS | 403 | 53 | RSPA | | | P |
| LOC100130710 | 19 | 62836685 | snp | C | T | D | 1343 | 302 | |  | | ATGAGC |
| LOC100130761 | 2 | 161999012 | ins |  | AC | FS | 194 | 64 | | T | | TL |
| LOC100130779 | 13 | 43876733 | ref | CTGT | CTGT | N | 467 | 155 | | * | | W |
| LOC100130790 | 17 | 77807688 | del | AAAA |  | FS | 59 | 19 | | KK | | N |
| LOC100130815 | 14 | 95812433 | del | A |  | FS | 38 | 12 | | C | | C |
| LOC100130825 | 12 | 130714627 | snp | G | A | NS | 288 | 96 | | R | | * |
| LOC100130837 | 10 | 1085412 | del | C |  | FS | 120 | 30 | | P | | P |
| LOC100130948 | 1 | 115680052 | ins |  | T | FS | 163 | 54 | | I | | N |
| LOC100130965 | 1 | 232559040 | del | T |  | FS | 247 | 82 | | L | | W |
| LOC100131022 | 7 | 13860772 | snp | C | G | D | 410 | 43 | |  | | ATCTTGTGTTTAAAC |
| LOC100131032 | 1 | 208697895 | snp | C | T | NS | 739 | 135 | | Q | | * |
| LOC100131089 | 15 | 38145272 | del | A |  | FS | 731 | 38 | | K | | N |
| LOC100131227 | 14 | 20018126 | del | A |  | FS | 63 | 21 | | F | | F |
| LOC100131354 | 1 | 204049352 | del | AGCC |  | FS | 785 | 261 | | GA | | G |
| LOC100131404 | 22 | 40684456 | del | AG |  | FS | 550 | 96 | | RV | | S |
| LOC100131409 | 2 | 145141870 | snp | C | G | NS | 17 | 5 | | Y | | * |
| LOC100131436 | 17 | 71598995 | del | C |  | FS | 1012 | 13 | | P | | Q |
| LOC100131439 | 17 | 70099921 | ref | TGGGGACAGCCACATGGTCCTG | TGGGGACAGCCACATGGTCCTG | MS | 75 | 0 | | MWLSPSLL | | QDHVASLL |
| LOC100131443 | 9 | 132762712 | del | TG |  | D | 310 | 103 | |  | | GTGACCACCTCTG |
| LOC100131446 | 12 | 47637585 | - | GCGATAGCT | ?AGANCN | D | 106 | 35 | |  | | CTAGCTACCGCGCNGNTCT? |
| LOC100131484 | 6 | 36756255 | ins |  | AG | FS | 561 | 187 | | F | | S |
| LOC100131542 | 3 | 25796097 | del | C |  | FS | 27 | 9 | | L | | W |
| LOC100131547 | 12 | 14658768 | del | AA |  | FS | 325 | 51 | | K | | S |
| LOC100131601 | 16 | 73838070 | del | C |  | FS | 420 | 34 | | V | | V |
| LOC100131672 | 9 | 111841707 | snp | C | T | D | 10 | 0 | |  | | AGTTAGAACTAGGAA |
| LOC100131780 | 9 | 34511596 | ins |  | A | FS | 105 | 2 | | G | | G |
| LOC100131811 | 4 | 184601401 | del | AC |  | FS | 1012 | 266 | | G | | G |
| LOC100131824 | 1 | 149852325 | del | G |  | FS | 951 | 26 | | P | | P |
| LOC100131891 | 16 | 34288047 | del | T |  | FS | 417 | 85 | | S | | P |
| LOC100131907 | 19 | 377973 | snp | C | T | MS | 138 | 0 | | M | | I |
| LOC100131949 | 11 | 65865235 | snp | C | T | NS | 907 | 269 | | Q | | * |
| LOC100131966 | 18 | 18066283 | del | TCCA |  | FS | 429 | 143 | | ST | | L |
| LOC100131984 | Y | 11784834 | sub | ATT | CTC | D | 4.29E+09 | -1 | |  | | AAACAGCGGTCCCCGAG |
| LOC100132033 | 17 | 43480189 | snp | C | T | NS | 2688 | 77 | | R | | * |
| LOC100132105 | 5 | 75477415 | snp | G | A | NS | 247 | 82 | | W | | * |
| LOC100132159 | 10 | 5548146 | ins |  | C | FS | 407 | 29 | | C | | W |
| LOC100132338 | 21 | 43063129 | del | G |  | FS | 251 | 83 | | G | | G |
| LOC100132339 | 16 | 53530562 | snp | G | A | NS | 387 | 129 | | Q | | * |
| LOC100132361 | 6 | 26435934 | snp | T | C | N | 1101 | 220 | | * | | Q |
| LOC100132444 | 14 | 19881316 | ins |  | C | FS | 264 | 71 | | L | | L |
| LOC100132506 | Y | 9952739 | ins |  | G | FS | 74 | 24 | | S | | S |
| LOC100132557 | X | 147946364 | ins |  | G | FS | 216 | 40 | | K | | E |
| LOC100132634 | 11 | 20648612 | del | G |  | FS | 426 | 142 | | P | | P |
| LOC100132713 | 18 | 74482672 | del | CTATGGAAGTTTCT |  | FS | 2959 | 986 | SMEVS | | | S |
| LOC100132730 | 10 | 50132517 | del | TG |  | FS | 367 | 122 | | L | | L |
| LOC100132731 | 3 | 130116788 | del | AG |  | FS | 1683 | 551 | | L | | V |
| LOC100132821 | 2 | 240503270 | del | CAG |  | FS | 599 | 199 | | CV | | C |
| LOC100132893 | 18 | 75616930 | ins |  | C | FS | 681 | 55 | | R | | R |
| LOC100132934 | 2 | 228443397 | ins |  | G | FS | 264 | 88 | | M | | H |
| LOC100132942 | 1 | 14912875 | sub | AAAAA | G | D | 841 | 0 | |  | | GAA |
| LOC100132973 | 19 | 9154524 | ins |  | G | FS | 200 | 66 | | S | | S |
| LOC100132980 | 15 | 18824191 | ins |  | GGGA | FS | 263 | 73 | | I | | RD |
| LOC100133003 | 2 | 87136403 | snp | C | G | N | 1117 | 372 | | * | | S |
| LOC100133042 | 12 | 6514251 | snp | C | G | N | 943 | 139 | | * | | S |
| LOC100133063 | 15 | 19542816 | snp | T | C | D | 1156 | 382 | |  | | AACCCCTGGTAACGG |
| LOC100133208 | 16 | 66022123 | ref | GCGATCAG | GCGATCAG | N | 1328 | 442 | | C* | | CR |
| LOC100133215 | 9 | 97823109 | ref | GTCCT | GTCCT | N | 1418 | 472 | | * | | C |
| LOC100133289 | 21 | 46313006 | sub | C | GGT | FS | 467 | 155 | | C | | WV |
| LOC100133292 | 5 | 2638079 | del | GATGGATG |  | FS | 1592 | 530 | FIHP | | | F |
| LOC100133301 | 1 | 16738360 | del | T |  | FS | 213 | 71 | | W | | G |
| LOC119358 | 10 | 91728804 | snp | T | C | N | 351 | 117 | | * | | Q |
| LOC120824 | 11 | 48956998 | snp | C | G | D | 737 | 245 | |  | | CTACGG |
| LOC123855 | 16 | 83776223 | snp | A | G | D | 2283 | 761 | |  | | TGTTTTGTCTTTCGG |
| LOC126075 | 19 | 11320666 | ins |  | A | FS | 660 | 15 | | E | | E |
| LOC127295 | 1 | 24207595 | snp | T | C | MS | 0 | 0 | | M | | V |
| LOC147645 | 19 | 56527705 | ins |  | G | FS | 2575 | 858 | | A | | A |
| LOC147670 | 19 | 61846157 | del | C |  | FS | 422 | 140 | | P | | P |
| LOC196346 | 11 | 49060265 | snp | G | T | NS | 801 | 267 | | E | | * |
| LOC283710 | 15 | 29308805 | del | GG |  | FS | 180 | 22 | | P | | P |
| LOC284297 | 19 | 60703261 | del | C |  | FS | 2059 | 657 | | P | | Q |
| LOC285346 | 3 | 44515799 | del | TC |  | FS | 1620 | 491 | | G | | G |
| LOC285908 | 7 | 65479368 | ref | AGCTGGGCCT | AGCTGGGCCT | N | 3511 | 217 | | * | | C |
| LOC339742 | 2 | 132827362 | snp | A | G | N | 736 | 245 | | * | | W |
| LOC341346 | 12 | 27514702 | ins |  | T | FS | 14 | 4 | | L | | F |
| LOC341689 | 13 | 58002084 | snp | G | C | D | 1663 | 0 | |  | | CTATTC |
| LOC341784 | 13 | 28063100 | snp | G | A | D | 385 | 0 | |  | | CTCTTTGTTTCCAAA |
| LOC344382 | 2 | 37897999 | del | A |  | FS | 438 | 146 | | R | | G |
| LOC344595 | 3 | 108446710 | snp | T | C | N | 1029 | 343 | | * | | R |
| LOC375295 | 2 | 177210558 | ins |  | C | FS | 1347 | 449 | | G | | G |
| LOC388076 | 15 | 19941660 | snp | A | G | N | 562 | 183 | | * | | R |
| LOC388177 | 15 | 91060195 | del | T |  | FS | 378 | 125 | | K | | K |
| LOC388780 | 20 | 2135942 | sub | GC | TT | NS | 152 | 50 | | PQ | | P* |
| LOC388963 | 2 | 74495232 | snp | G | A | NS | 1986 | 529 | | Q | | * |
| LOC389151 | 3 | 140222173 | ref | CTGCGGGGCCGGCTGACCATCGCCT | CTGCGGGGCCGGCTGACCATCGCCT | MS | 265 | 0 | | MVSRPRSPS | | RRWSAGPPS |
| LOC389333 | 5 | 138758662 | ref | TCAGCATGGTGGG | TCAGCATGGTGGG | MS | 115 | 0 | | MLTAL | | PTTAL |
| LOC389370 | 6 | 22752189 | del | GCGGGCTGCTGGC |  | FS | 273 | 38 | CQQPA | | | C |
| LOC391282 | 21 | 39420970 | snp | T | C | D | 8 | 2 | |  | | GCGAGT |
| LOC392288 | 9 | 19461866 | del | A |  | FS | 658 | 219 | | I | | I |
| LOC392364 | 9 | 89664168 | snp | T | C | D | 166 | 0 | |  | | TCCCTGCTTCCGCGG |
| LOC400950 | 2 | 42019257 | snp | C | T | D | 468 | 125 | |  | | TCCTCTTTCTTGCAA |
| LOC400986 | 2 | 96021170 | ref | CCTTGTCATCCATAATGGTCGGCTGCA | CCTTGTCATCCATAATGGTCGGCTGCA | MS | 248 | 0 | | MDDKEPKRW | | CSRP* |
| LOC440287 | 15 | 72179598 | del | A |  | FS | 600 | 200 | | Y | | M |
| LOC440419 | 17 | 22769813 | snp | T | A | D | 422 | 0 | |  | | GAGAGC |
| LOC440981 | 3 | 147596268 | del | T |  | FS | 546 | 182 | | K | | K |
| LOC441054 | 4 | 186590345 | del | A |  | FS | 545 | 105 | | K | | S |
| LOC441730 | 15 | 80410899 | snp | A | G | D | 4720 | 0 | |  | | GTGAGT |
| LOC441964 | 21 | 38541142 | del | TGGA |  | FS | 844 | 281 | | IH | | T |
| LOC642502 | 17 | 1708195 | snp | G | A | NS | 303 | 98 | | R | | * |
| LOC644366 | 17 | 42263261 | snp | C | T | NS | 571 | 190 | | W | | * |
| LOC644578 | 4 | 63898085 | snp | T | C | D | 249 | 83 | |  | | GCGAGG |
| LOC644790 | 1 | 45018829 | snp | C | A | NS | 202 | 48 | | E | | * |
| LOC644974 | 3 | 131297625 | del | C |  | FS | 507 | 169 | | L | | W |
| LOC645018 | 4 | 144490515 | del | AGAT |  | FS | 780 | 67 | | IY | | I |
| LOC645273 | 2 | 216876545 | snp | G | A | NS | 142 | 15 | | W | | * |
| LOC645277 | 12 | 131691471 | snp | T | C | D | 244 | 81 | |  | | CGGGGAGCCATGCGG |
| LOC645733 | 2 | 118659430 | ins |  | GGCAGGAC | FS | 453 | 151 | | V | | VLP |
| LOC645752 | 15 | 76001429 | snp | T | C | D | 848 | 282 | |  | | GTAACG |
| LOC645937 | 9 | 89749701 | ins |  | G | FS | 68 | 22 | | R | | R |
| LOC646090 | 15 | 18713927 | snp | A | G | D | 1199 | 0 | |  | | GCGTGT |
| LOC646268 | 1 | 156368733 | snp | T | G | N | 372 | 75 | | * | | S |
| LOC646360 | 10 | 37638251 | snp | A | T | NS | 133 | 13 | | K | | * |
| LOC646396 | 15 | 20049014 | snp | T | C | D | 1485 | 495 | |  | | GCATGT |
| LOC646471 | 1 | 26019808 | del | G |  | FS | 2032 | 9 | | T | | T |
| LOC646609 | 9 | 22724372 | snp | T | C | D | 53 | 0 | |  | | GCAGAT |
| LOC646805 | 16 | 20511169 | ins |  | G | FS | 830 | 182 | | V | | V |
| LOC646934 | 15 | 76817632 | snp | A | C | D | 1521 | 0 | |  | | CTCCTGTCTGCACCG |
| LOC647131 | 13 | 48447790 | ref | GCCTGGCCACAGCGGATGCTACCACAA | GCCTGGCCACAGCGGATGCTACCACAA | N | 492 | 164 | | LW* | | WPG |
| LOC647166 | 13 | 50584028 | ins |  | G | FS | 332 | 110 | | V | | V |
| LOC648154 | 1 | 220952742 | ref | TCATGGCCGCCGGGTCACGTGACTC | TCATGGCCGCCGGGTCACGTGACTC | MS | 1124 | 0 | | MIATEAVVR | | ELATEAVVR |
| LOC648665 | 8 | 6928011 | snp | G | C | D | 109 | 0 | |  | | CTAAAG |
| LOC648822 | 1 | 153388341 | del | T |  | FS | 439 | 146 | | L | | * |
| LOC653631 | 1 | 78050052 | ref | ATGG | ATGG | MS | 159 | 0 | | MS | | PS |
| LOC727827 | 6 | 469893 | ins |  | GAGT | FS | 154 | 26 | | V | | VS |
| LOC727950 | 10 | 47805966 | ref | ATG | ATG | MS | 1374 | 0 | | M | | Q |
| LOC728119 | 6 | 108585631 | ref | CGCGAACCGCTCAG | CGCGAACCGCTCAG | N | 1647 | 126 | | G* | | GA |
| LOC728135 | 3 | 75512114 | ins |  | CA | FS | 380 | 126 | | S | | SV |
| LOC728148 | 12 | 46094948 | snp | G | A | D | 393 | 131 | |  | | CATCTGTGACAAAAA |
| LOC728203 | 4 | 186147200 | sub | TG | CA | MS | 569 | 0 | | M | | T |
| LOC728275 | 6 | 164267962 | snp | C | T | NS | 321 | 107 | | Q | | * |
| LOC728285 | 17 | 36469810 | ref | GCAGCAGGAGCCGGTCATG | GCAGCAGGAGCCGGTCATG | MS | 58 | 0 | | MTGSCCG | | HDRLLLG |
| LOC728318 | 17 | 36599664 | snp | A | G | MS | 0 | 0 | | M | | V |
| LOC728450 | 20 | 11738887 | ins |  | TT | FS | 271 | 70 | | E | | E |
| LOC728459 | 15 | 96449384 | snp | A | G | MS | 176 | 0 | | M | | T |
| LOC728597 | 2 | 3728931 | del | G |  | FS | 243 | 81 | | V | | F |
| LOC728621 | 1 | 42774801 | del | GA |  | FS | 149 | 19 | | EK | | E |
| LOC728635 | 14 | 23582863 | del | G |  | FS | 364 | 121 | | R | | K |
| LOC728647 | 10 | 59943202 | del | CCGG |  | FS | 1367 | 89 | | NR | | N |
| LOC728677 | 17 | 78127131 | snp | G | A | NS | 1195 | 122 | | R | | * |
| LOC728727 | 6 | 120207435 | snp | C | T | NS | 291 | 97 | | R | | * |
| LOC728969 | 1 | 21627933 | snp | C | T | NS | 667 | 184 | | R | | * |
| LOC728991 | 17 | 76052952 | snp | C | T | NS | 1107 | 135 | | R | | * |
| LOC729026 | 2 | 181685007 | del | A |  | FS | 292 | 97 | | K | | R |
| LOC729054 | 10 | 53738611 | sub | AAT | GG | MS | 0 | 0 | | M | | G |
| LOC729112 | 4 | 122630619 | del | C |  | FS | 391 | 50 | | S | | * |
| LOC729421 | 5 | 151130449 | del | T |  | FS | 271 | 3 | | E | | G |
| LOC729479 | 13 | 79498382 | ins |  | A | FS | 146 | 16 | | Q | | T |
| LOC729534 | 3 | 135802412 | snp | T | A | N | 639 | 97 | | * | | K |
| LOC729537 | 9 | 89630040 | snp | T | A | D | 368 | 122 | |  | | GATCAC |
| LOC729568 | 22 | 20059867 | snp | A | G | N | 907 | 302 | | * | | W |
| LOC729595 | 5 | 179054405 | ins |  | AA | FS | 602 | 200 | | K | | KS |
| LOC729601 | 10 | 29201986 | del | C |  | D | 379 | 126 | |  | | TTCTCTGATCTACA |
| LOC729605 | 11 | 31794829 | ins |  | G | FS | 1536 | 512 | | R | | P |
| LOC729616 | 6 | 153346280 | snp | C | T | NS | 327 | 109 | | R | | * |
| LOC729629 | X | 1473358 | del | CCTCTCTCTTTCCTGCCTTTCCCCATCTCTCCCTTTCCTGCCTTTCCCTG |  | FS | 436 | 145 | ASLFPAFPHLSLSCLSL | | | V |
| LOC729637 | 14 | 61081591 | ins |  | C | FS | 167 | 5 | | R | | R |
| LOC729639 | 16 | 2330194 | ref | GCTGTCGGTCGCTACA | GCTGTCGGTCGCTACA | N | 1207 | 402 | | M* | | NS |
| LOC729659 | 7 | 102689898 | snp | C | T | NS | 216 | 72 | | Q | | * |
| LOC729663 | 10 | 30437799 | del | A |  | FS | 634 | 211 | | K | | R |
| LOC729675 | 4 | 144325147 | snp | G | A | NS | 531 | 177 | | R | | * |
| LOC729722 | 15 | 18344204 | snp | T | A | NS | 245 | 81 | | Y | | * |
| LOC729878 | 16 | 3440040 | sub | GAAAAG | A | FS | 240 | 67 | | EK | | K |
| LOC729978 | 16 | 11934975 | del | AT |  | FS | 1757 | 111 | | H | | R |
| LOC729987 | 1 | 98448954 | snp | G | A | D | 100 | 0 | |  | | ATAAAA |
| LOC730079 | 16 | 20685432 | snp | C | T | NS | 43 | 14 | | W | | * |
| LOC730085 | 2 | 132778692 | sub | C | TG | D | 106 | 35 | |  | | CATGCAT |
| LOC730141 | 6 | 52174605 | snp | G | A | NS | 61 | 2 | | W | | * |
| LOC730291 | 7 | 63305037 | del | C |  | FS | 22 | 7 | | P | | L |
| LOC730382 | 7 | 56720601 | ins |  | T | FS | 760 | 253 | | E | | E |
| LOC730512 | 12 | 39186403 | snp | G | A | D | 642 | 214 | |  | | ATAATT |
| LOC731852 | 19 | 6905724 | snp | G | A | NS | 318 | 90 | | W | | * |
| LOC731954 | 10 | 31040315 | sub | C | TA | FS | 242 | 80 | | H | | HT |
| LOC732199 | 11 | 17171692 | - | AGG | GGN | D | 134 | 0 | |  | | GGNTGTGG |
| LOC92973 | 9 | 35850654 | del | CATT |  | FS | 384 | 26 | | FI | | L |
| LOC96597 | 17 | 16771011 | snp | T | C | D | 682 | 0 | |  | | GTGATGGGTCGGCGG |
| LONP2 | 16 | 46861535 | del | A |  | FS | 1179 | 363 | | N | | I |
| LPP | 3 | 189606732 | del | A |  | FS | 376 | 43 | | K | | S |
| LRBA | 4 | 151728666 | del | T |  | FS | 6819 | 2115 | | K | | K |
| LRP2 | 2 | 169927152 | ref | CATCTCCGCGAC | CATCTCCGCGAC | MS | 213 | 0 | | MDRG | | VDRG |
| LRRC47 | 1 | 3702899 | ref | TGGCG | TGGCG | MS | 28 | 0 | | MA | | LA |
| LRRC59 | 17 | 45815344 | ref | AGCTCA | AGCTCA | N | 1075 | 307 | | * | | A |
| LRRIQ1 | 12 | 84162785 | del | A |  | FS | 5215 | 1701 | | N | | I |
| LRRK2 | 12 | 39000143 | del | A |  | FS | 5034 | 1638 | | R | | G |
| LRRTM3 | 10 | 68357202 | snp | C | T | NS | 1072 | 174 | | R | | * |
| LSM2 | 6 | 31882505 | ref | CCCACCATGG | CCCACCATGG | MS | 209 | 0 | | MLFY | | PLFY |
| LSS | 21 | 46438929 | del | C |  | FS | 1922 | 630 | | E | | R |
| LTA4H | 12 | 94933530 | del | A |  | FS | 1087 | 339 | | H | | H |
| MAB21L1 | 13 | 34947359 | snp | G | A | NS | 1434 | 305 | | Q | | * |
| MACC1 | 7 | 20165249 | del | C |  | FS | 1567 | 419 | | W | | W |
| MADD | 11 | 47263205 | snp | C | T | NS | 2497 | 765 | | R | | * |
| MAGEB16 | X | 35731047 | snp | C | T | NS | 1092 | 271 | | R | | * |
| MAGEC1 | X | 140822911 | del | G |  | FS | 2341 | 685 | | E | | R |
| MALL | 2 | 110230650 | ref | GAGGCCATGC | GAGGCCATGC | MS | 279 | 0 | | MASP | | AWPP |
| MAN2B1 | 19 | 12620983 | del | C |  | FS | 2714 | 800 | | G | | A |
| MAP2K3 | 17 | 21144802 | snp | C | T | NS | 553 | 72 | | Q | | * |
| MAP3K6 | 1 | 27565674 | ref | TGCG | TGCG | MS | 249 | 0 | | MA | | LA |
| MARCH1 | 4 | 164669373 | del | G |  | FS | 914 | 264 | | G | | G |
| MARCH4 | 2 | 216942838 | del | C |  | FS | 2156 | 129 | | L | | L |
| MARCH5 | 10 | 94041231 | snp | A | G | MS | 332 | 0 | | M | | V |
| MARCH7 | 2 | 160312932 | del | T |  | FS | 1007 | 295 | | S | | H |
| MARK1 | 1 | 218858439 | del | G |  | FS | 1314 | 239 | | V | | C |
| MAST3 | 19 | 18119431 | - | CAGGTGGGTGCACCCCGACCCCCCACCA | ?G | D | 3394 | 1131 | |  | | ?G |
| MAST4 | 5 | 66484238 | del | C |  | FS | 2814 | 915 | | P | | L |
| MATR3 | 5 | 138671710 | del | T |  | FS | 1024 | 235 | | F | | L |
| MAX | 14 | 64638790 | ref | ATGTCATCGTTATCGCTCATTTCCTACGGCCCAGGG | ATGTCATCGTTATCGCTCATTTCCTACGGCCCAGGG | MS | 170 | 0 | | MSDNDDIEVESD | | PWAVGNDEVESD |
| MBD6 | 12 | 56208005 | del | G |  | FS | 2568 | 781 | | G | | E |
| MBP | 18 | 72857759 | snp | A | G | N | 855 | 197 | | * | | Q |
| MCOLN2 | 1 | 85176250 | snp | C | A | D | 1452 | 403 | |  | | TTAAGG |
| MEF2D | 1 | 154705382 | del | C |  | FS | 1449 | 353 | | G | | G |
| MEGF8 | 19 | 47564600 | del | G |  | FS | 6861 | 2075 | | G | | A |
| METTL10 | 10 | 126470372 | ref | CCGTCAGCGCCCGAGCTCATTTCGCTCCACGTCCTGGACGGCCGT | CCGTCAGCGCCCGAGCTCATTTCGCTCCACGTCCTGGACGGCCGT | MS | 37 | 0 | | MSSGADGGGGAAVAA | | TAVQDVDGGGAAVAA |
| MFRP | 11 | 118721488 | del | G |  | FS | 638 | 163 | | Y | | Y |
| MGC16703 | 22 | 19693743 | snp | T | C | D | 712 | 0 | |  | | TCTGGTTTCTCTCGG |
| MGC24975 | 19 | 5735169 | del | G |  | FS | 440 | 27 | | P | | P |
| MGC3196 | 11 | 62316039 | ref | CATGGCCAGCCGACTT | CATGGCCAGCCGACTT | MS | 20 | 0 | | MAAPWR | | KAAPWR |
| MGC33894 | 17 | 21087331 | ref | CGGCCTGGGAGCCGGGCAGA | CGGCCTGGGAGCCGGGCAGA | FS | 227 | 75 | | AALDFVV | | AAPGLRG |
| MGC34821 | 11 | 62605062 | snp | A | C | NS | 1944 | 500 | | Y | | * |
| MGST2 | 4 | 140844632 | snp | C | T | NS | 576 | 108 | | R | | * |
| MIER2 | 19 | 257684 | ref | AGGAGTCAG | AGGAGTCAG | N | 1643 | 544 | | C* | | CP |
| MLH3 | 14 | 74584364 | del | T |  | FS | 1962 | 582 | | K | | K |
| MLL3 | 7 | 151505088 | del | T |  | FS | 8600 | 2793 | | P | | P |
| MLL4 | 19 | 40906472 | ins |  | G | D | 3058 | 1019 | |  | | CCCCTGTTCCCGCAGG |
| MLLT1 | 19 | 6164051 | ref | GTCATGTGGCCACGGCCTCCAGGCAG | GTCATGTGGCCACGGCCTCCAGGCAG | N | 1819 | 551 | | SCLEAVAT* | | SAWRPWPHD |
| MLXIPL | 7 | 72676745 | ref | GCGCGCCGGCCATGGCTGTCGCCGCCGCAACCGCCT | GCGCGCCGGCCATGGCTGTCGCCGCCGCAACCGCCT | MS | 48 | 0 | | MAGALAGLAAGL | | RRLRLAGLAAGL |
| MMEL1 | 1 | 2550753 | ref | CATCCCCACT | CATCCCCACT | MS | 189 | 0 | | MVES | | SVES |
| MOBKL2C | 1 | 46853265 | snp | G | A | NS | 126 | 23 | | R | | * |
| MPHOSPH6 | 16 | 80761276 | ref | GCCATGGTAGCTTC | GCCATGGTAGCTTC | MS | 49 | 0 | | MAAER | | EAAER |
| MRGPRG | 11 | 3198079 | snp | A | G | MS | 1 | 0 | | M | | T |
| MRPL19 | 2 | 75735493 | del | T |  | FS | 624 | 199 | | F | | L |
| MS4A14 | 11 | 59921928 | del | TT |  | FS | 731 | 55 | | I | | S |
| MSH2 | 2 | 47561512 | del | AG |  | FS | 2700 | 877 | | E | | A |
| MSH6 | 2 | 47884150 | del | C |  | FS | 3412 | 1086 | | P | | P |
| MSLNL | 16 | 765446 | ins |  | G | FS | 1315 | 438 | | H | | P |
| MTCH2 | 11 | 47609832 | snp | G | A | NS | 422 | 125 | | R | | * |
| MTF2 | 1 | 93372341 | snp | T | C | D | 1665 | 474 | |  | | GCAGAT |
| MTRF1 | 13 | 40689246 | ref | GTATTTATTTTGCTGATTTAAGGTGTTCATCCAAAAGTTCAGCAATG | GTATTTATTTTGCTGATTTAAGGTGTTCATCCAAAAGTTCAGCAATG | N | 1396 | 431 | | AIAELLDEHLKSAK* | | ALNFWMNTLNQQNKY |
| MUC1 | 1 | 153429253 | ref | GTCATGGTGGTGGT | GTCATGGTGGTGGT | MS | 66 | 0 | | MTPGT | | TTPGT |
| MUC19 | 12 | 39121221 | snp | C | A | NS | 2336 | 653 | | C | | * |
| MUC2 | 11 | 1091648 | ref | T | T | FS | 7269 | 2414 | | P | | F |
| MUC4 | 3 | 197001272 | ref | T | T | FS | 2048 | 529 | | A | | D |
| MUC6 | 11 | 1008094 | sub | G | TA | FS | 4756 | 1568 | | P | | L |
| MYCL1 | 1 | 40139686 | ref | AGTCCATG | AGTCCATG | MS | 217 | 0 | | MDY | | HGH |
| MYEOV | 11 | 68820305 | del | G |  | FS | 1262 | 270 | | G | | G |
| MYH11 | 16 | 15710193 | del | GG |  | FS | 5897 | 1930 | | P | | P |
| MYL2 | 12 | 109833257 | ref | CCCCTCCTAG | CCCCTCCTAG | N | 545 | 165 | | D* | | EG |
| MYL6 | 12 | 54839741 | ins |  | G | FS | 221 | 45 | | A | | G |
| MYLK | 3 | 124940528 | del | T |  | FS | 774 | 164 | | T | | P |
| MYO15B | 17 | 71114680 | snp | T | C | D | 4166 | 0 | |  | | GCGACA |
| MYO7A | 11 | 76551614 | del | C |  | FS | 1888 | 540 | | P | | P |
| MYSM1 | 1 | 58899663 | snp | A | G | D | 2292 | 756 | |  | | GCATGT |
| NANOS2 | 19 | 51109370 | ref | ATGCTCAG | ATGCTCAG | N | 498 | 137 | | R* | | RH |
| NAT10 | 11 | 34111220 | del | C |  | FS | 1895 | 563 | | P | | P |
| NAT8B | 2 | 73781438 | ref | A | A | NS | 536 | 167 | | Q | | * |
| NBEAL2 | 3 | 47022291 | del | C |  | FS | 6930 | 2250 | | P | | R |
| NCAPD2 | 12 | 6505740 | ins |  | C | FS | 2566 | 836 | | F | | L |
| NCRNA00119 | 3 | 133995148 | snp | G | A | D | 330 | 0 | |  | | ATAAGT |
| NDFIP2 | 13 | 78993031 | ref | GTCTTCAGCACCAGCACTTGAA | GTCTTCAGCACCAGCACTTGAA | NS | 459 | 135 | | VSSAPALE | | AVFSTST* |
| NDST2 | 10 | 75235367 | snp | G | A | NS | 2285 | 576 | | R | | * |
| NEIL2 | 8 | 11678119 | snp | G | T | D | 1081 | 163 | |  | | GTTCTTTCTTCCCAT |
| NEK3 | 13 | 51616058 | ref | C | C | FS | 1103 | 289 | | R | | R |
| NF1 | 17 | 26678678 | snp | C | T | NS | 5637 | 1768 | | R | | * |
| NFU1 | 2 | 69512629 | snp | A | T | MS | 362 | 0 | | M | | K |
| NIPA2 | 15 | 20572686 | del | T |  | FS | 581 | 30 | | K | | K |
| NKRF | X | 118608725 | ins |  | T | FS | 826 | 230 | | K | | K |
| NKX2-6 | 8 | 23620926 | ins |  | G | FS | 144 | 48 | | P | | P |
| NKX2-8 | 14 | 36119818 | ref | TCCAAAGTCGAGGGTAGCCCCAGGTGCCGCCCTGCGGCCTCAC | TCCAAAGTCGAGGGTAGCCCCAGGTGCCGCCCTGCGGCCTCAC | N | 908 | 238 | | W* | | CG |
| NKX6-1 | 4 | 85633429 | ref | AGGCCGGAGCCGGGAAGGTGCGGCGGGCGGCGGCGTTCA | AGGCCGGAGCCGGGAAGGTGCGGCGGGCGGCGGCGTTCA | N | 1107 | 367 | | * | | P |
| NKX6-3 | 8 | 41623107 | ref | GGCCTGGACGGCGGGCGTCA | GGCCTGGACGGCGGGCGTCA | N | 409 | 135 | | * | | A |
| NLK | 17 | 23542227 | snp | C | T | NS | 1502 | 430 | | R | | * |
| NLRP12 | 19 | 59000748 | snp | C | A | MS | 1310 | 0 | | M | | I |
| NLRP7 | 19 | 60137667 | snp | C | T | D | 2456 | 795 | |  | | ATAAGT |
| NLRP8 | 19 | 61191090 | snp | G | C | N | 3217 | 1048 | | * | | Y |
| NOS3 | 7 | 150323228 | del | C |  | FS | 456 | 54 | | P | | R |
| NOTCH3 | 19 | 15137867 | ins |  | C | FS | 5473 | 1799 | | G | | G |
| NPAL2 | 8 | 99274787 | snp | C | T | D | 1322 | 355 | |  | | ATGAGT |
| NPDC1 | 9 | 139055236 | ins |  | C | FS | 620 | 135 | | R | | R |
| NPSR1 | 7 | 34884264 | snp | C | T | NS | 1205 | 359 | | R | | * |
| NR1H4 | 12 | 99411232 | snp | A | G | MS | 348 | 0 | | M | | V |
| NRIP1 | 21 | 15260207 | del | T |  | FS | 2510 | 725 | | E | | E |
| NRM | 6 | 30766719 | ref | GCAGGGGCCATGGCGAGAAATGGAGGGG | GCAGGGGCCATGGCGAGAAATGGAGGGG | MS | 18 | 0 | | MAPALLLIPA | | PLHLLLLIPA |
| NT5M | 17 | 17190921 | ins |  | C | FS | 807 | 207 | | R | | P |
| NTNG2 | 9 | 134095785 | ref | CTG | CTG | NS | 1121 | 345 | | AA | | T* |
| OAS1 | 12 | 111841575 | snp | G | A | D | 1144 | 346 | |  | | GTCTCACCCTTTCAA |
| OAS2 | 12 | 111932670 | snp | A | G | N | 2298 | 719 | | * | | W |
| OBFC1 | 10 | 105667207 | snp | A | G | D | 303 | 44 | |  | | GCATGT |
| OCIAD1 | 4 | 48554135 | ins |  | A | FS | 1200 | 232 | | E | | R |
| ODF3B | 22 | 49315771 | ref | GGGTCAGTTGTCCGCG | GGGTCAGTTGTCCGCG | NS | 886 | 249 | | DADN* | | EDN* |
| ODZ2 | 5 | 167576348 | snp | A | G | D | 3479 | 1120 | |  | | GCTTGTCTTGCTCGG |
| OFD1 | X | 13674874 | del | A |  | FS | 1020 | 236 | | K | | S |
| OGG1 | 3 | 9782561 | snp | C | T | NS | 1327 | 339 | | R | | * |
| OLFML1 | 11 | 7466000 | del | A |  | FS | 548 | 65 | | N | | I |
| OMA1 | 1 | 58719279 | del | T |  | FS | 1607 | 506 | | Q | | Q |
| OPA1 | 3 | 194837763 | - | GGTAT | NNNNC | D | 979 | 308 | |  | | NNNNCGT |
| OPRS1 | 9 | 34627670 | ref | CCGCCGGCCCACGGCCCACTGCATCCCGGCGGG | CCGCCGGCCCACGGCCCACTGCATCCCGGCGGG | MS | 74 | 0 | | MQWAVGRRWAW | | PAGMQWAVWAW |
| OR10X1 | 1 | 156816115 | snp | C | T | NS | 197 | 65 | | W | | * |
| OR11G2 | 14 | 19736021 | ins |  | A | FS | 687 | 229 | | G | | R |
| OR13A1 | 10 | 45119072 | ins |  | A | FS | 1113 | 268 | | Y | | L |
| OR1B1 | 9 | 124431598 | ins |  | A | FS | 37 | 12 | | V | | V |
| OR2T4 | 1 | 246592261 | del | A |  | FS | 756 | 252 | | I | | S |
| OR4C3 | 11 | 48303589 | snp | G | A | NS | 521 | 173 | | W | | * |
| OR4D9 | 11 | 59039378 | snp | C | T | NS | 417 | 139 | | R | | * |
| OR4X1 | 11 | 48242806 | snp | T | A | NS | 818 | 272 | | Y | | * |
| OR51Q1 | 11 | 5400711 | snp | C | T | NS | 705 | 235 | | R | | * |
| OR52E8 | 11 | 5834554 | snp | T | A | N | 953 | 317 | | * | | Y |
| OR52N4 | 11 | 5733059 | snp | A | T | NS | 513 | 171 | | R | | * |
| OR5AR1 | 11 | 56187791 | snp | C | T | NS | 54 | 18 | | Q | | * |
| OR5D16 | 11 | 55362994 | ins |  | T | FS | 191 | 63 | | F | | F |
| OR5K2 | 3 | 99699867 | snp | T | A | NS | 653 | 217 | | Y | | * |
| OR5K3 | 3 | 99593103 | ins |  | A | FS | 904 | 301 | | I | | N |
| OR5K4 | 3 | 99556287 | del | A |  | FS | 900 | 300 | | I | | L |
| OR5M1 | 11 | 56137128 | del | GACA |  | FS | 422 | 140 | | CV | | C |
| OR5M10 | 11 | 56101421 | del | TTGAAGCA |  | FS | 344 | 114 | LASM | | | L |
| OR5M11 | 11 | 56066931 | snp | A | T | NS | 377 | 125 | | Y | | * |
| OR6C4 | 12 | 54232065 | del | A |  | FS | 788 | 262 | | K | | K |
| OR6Q1 | 11 | 57555684 | del | C |  | FS | 684 | 228 | | L | | C |
| OR7D4 | 19 | 9186207 | del | A |  | FS | 305 | 101 | | Y | | Y |
| OR7E24 | 19 | 9222750 | del | T |  | FS | 31 | 10 | | F | | S |
| OR7G3 | 19 | 9097705 | ins |  | GGTAT | FS | 921 | 307 | | I | | IP |
| OR8K1 | 11 | 55870091 | snp | T | C | MS | 1 | 0 | | M | | T |
| OSBP | 11 | 59134540 | del | C |  | FS | 939 | 153 | | G | | G |
| OTUD7B | 1 | 148205983 | del | C |  | FS | 716 | 120 | | G | | G |
| OVCH2 | 11 | 7669046 | snp | C | T | NS | 1667 | 555 | | W | | * |
| P2RX5 | 17 | 3541029 | del | G |  | FS | 612 | 109 | | T | | T |
| PADI6 | 1 | 17591260 | del | G |  | FS | 1026 | 342 | | V | | W |
| PAH | 12 | 101770783 | snp | G | A | NS | 1252 | 260 | | R | | * |
| PARN | 16 | 14612112 | del | T |  | FS | 499 | 147 | | E | | E |
| PAX8 | 2 | 113710769 | ins |  | C | D | 943 | 259 | |  | | CTCTTTGCTCCCCAGG |
| PCDH19 | X | 99549166 | del | G |  | FS | 2760 | 361 | | A | | A |
| PCDHA4 | 5 | 140167171 | sub | AGG | GA | FS | 316 | 71 | | GG | | GT |
| PCDHAC2 | 5 | 140327765 | snp | C | T | NS | 1470 | 410 | | R | | * |
| PCDHB13 | 5 | 140574600 | del | C |  | FS | 908 | 240 | | P | | L |
| PCSK1 | 5 | 95773856 | del | C |  | FS | 1008 | 267 | | W | | W |
| PCSK4 | 19 | 1435126 | sub | CTGA | GT | D | 1129 | 355 | |  | | CCCCTCTGCCCAC |
| PCSK6 | 15 | 99739818 | snp | C | G | D | 1845 | 510 | |  | | CTAAGG |
| PDE4DIP | 1 | 143787039 | snp | C | T | NS | 396 | 59 | | W | | * |
| PDLIM1 | 10 | 96987721 | snp | G | A | NS | 1048 | 313 | | R | | * |
| PDLIM2 | 8 | 22503144 | snp | C | T | NS | 842 | 236 | | R | | * |
| PDS5A | 4 | 39526885 | del | A |  | FS | 3858 | 1106 | | F | | F |
| PDZD7 | 10 | 102768889 | ins |  | G | FS | 1218 | 334 | | A | | A |
| PEMT | 17 | 17435663 | ref | ATCCG | ATCCG | MS | 54 | 0 | | MK | | RK |
| PFTK1 | 7 | 90366582 | del | G |  | FS | 770 | 208 | | G | | G |
| PHACTR2 | 6 | 144123395 | del | A |  | FS | 818 | 206 | | N | | I |
| PHACTR4 | 1 | 28658325 | del | A |  | FS | 424 | 63 | | S | | V |
| PHF2 | 9 | 95462439 | del | A |  | FS | 1621 | 491 | | K | | R |
| PHF21A | 11 | 45912188 | del | G |  | FS | 2434 | 603 | | T | | T |
| PI4KB | 1 | 149546735 | del | A |  | FS | 1224 | 385 | | F | | L |
| PIF1 | 15 | 62903585 | ref | ATCGTC | ATCGTC | MS | 66 | 0 | | ML | | EL |
| PIGB | 15 | 53418801 | del | T |  | FS | 1155 | 279 | | F | | L |
| PIGN | 18 | 57956511 | snp | T | C | D | 1564 | 372 | |  | | TACTTGGTTCCCAGG |
| PIK3R6 | 17 | 8665941 | ins |  | G | FS | 2065 | 608 | | S | | S |
| PITPNM1 | 11 | 67028225 | ref | ATCCTGA | ATCCTGA | MS | 189 | 0 | | MLI | | SLI |
| PITPNM2 | 12 | 122037918 | del | G |  | FS | 3413 | 1091 | | T | | S |
| PKD1L2 | 16 | 79799698 | snp | G | A | NS | 657 | 219 | | Q | | * |
| PLA2G12A | 4 | 110870407 | ref | GGGCCATG | GGGCCATG | MS | 246 | 0 | | MAL | | HGL |
| PLA2G2C | 1 | 20374168 | snp | G | A | NS | 105 | 35 | | R | | * |
| PLA2G4E | 15 | 40063984 | del | G |  | FS | 2239 | 746 | | A | | A |
| PLCD3 | 17 | 40548076 | ins |  | C | FS | 1735 | 540 | | R | | R |
| PLCL2 | 3 | 17027869 | del | T |  | FS | 1902 | 549 | | F | | F |
| PLEC1 | 8 | 145096858 | ref | CCATG | CCATG | MS | 170 | 0 | | MV | | HV |
| PLEKHA5 | 12 | 19176591 | snp | A | G | D | 175 | 56 | |  | | TATGTATTGTTTTGG |
| PLEKHH2 | 2 | 43780767 | del | A |  | FS | 1241 | 388 | | K | | N |
| PLIN | 15 | 88013841 | ins |  | A | FS | 788 | 221 | | V | | V |
| PLOD2 | 3 | 147361454 | ref | GCATCCCCCCATATTCGGCCCTCGA | GCATCCCCCCATATTCGGCCCTCGA | MS | 506 | 0 | | MGGCTVKPQ | | SRAETVKPQ |
| PMS2 | 7 | 5996052 | ins |  | A | FS | 1135 | 349 | | L | | L |
| PNLIPRP2 | 10 | 118373454 | ins |  | G | FS | 84 | 20 | | V | | G |
| POLD3 | 11 | 74014264 | del | A |  | FS | 972 | 299 | | R | | G |
| POLR3K | 16 | 43581 | ref | AGCATGGTCT | AGCATGGTCT | MS | 39 | 0 | | MLLF | | RPLF |
| PON3 | 7 | 94863572 | ref | AGGACCAGCGCCACGAGCTTCCCCATGGTCT | AGGACCAGCGCCACGAGCTTCCCCATGGTCT | MS | 25 | 0 | | MGKLVALVLLG | | RPWGSSWRWLG |
| PP8961 | 14 | 91110343 | del | C |  | FS | 792 | 121 | | A | | A |
| PPIG | 2 | 170171867 | snp | C | T | NS | 472 | 84 | | R | | * |
| PPL | 16 | 4884506 | del | G |  | FS | 1445 | 451 | | I | | I |
| PPM1A | 14 | 59819846 | snp | G | T | NS | 1023 | 224 | | E | | * |
| PPM1G | 2 | 27485788 | ref | CCCATG | CCCATG | MS | 207 | 0 | | MG | | HG |
| PPP2R5D | 6 | 43082307 | del | C |  | FS | 320 | 78 | | Q | | R |
| PRB3 | 12 | 11311602 | del | G |  | FS | 758 | 240 | | P | | L |
| PRB4 | 12 | 11353068 | snp | G | A | NS | 148 | 38 | | R | | * |
| PRIM2 | 6 | 57575058 | ref | G | G | NS | 1127 | 346 | | Y | | * |
| PRKCDBP | 11 | 6298278 | ref | TCATGATCCCTGAC | TCATGATCCCTGAC | MS | 34 | 0 | | MRESA | | VRESA |
| PRKDC | 8 | 49035220 | ref | CGGCTCCGGAGCCCGCCATGCCGCCGAGTC | CGGCTCCGGAGCCCGCCATGCCGCCGAGTC | MS | 57 | 0 | | MAGSGAGVRC | | DSAAWRGVRC |
| PRPF8 | 17 | 1532171 | snp | C | G | D | 547 | 144 | |  | | CTGAGA |
| PRRG1 | X | 37197538 | del | C |  | FS | 565 | 133 | | P | | H |
| PRRT2 | 16 | 29732516 | ins |  | C | FS | 941 | 213 | | A | | A |
| PRTFDC1 | 10 | 25281509 | ref | TGTTTCTCCCGGGGAACGCGGGAAGGGAAGACG | TGTTTCTCCCGGGGAACGCGGGAAGGGAAGACG | MS | 29 | 0 | | MAGSSEEAPDY | | LAGSSEEAPDY |
| PSD | 10 | 104166782 | ref | CATGCTGGGGCCGGGG | CATGCTGGGGCCGGGG | MS | 527 | 0 | | MAQGAM | | PAQGAM |
| PSMD5 | 9 | 122644994 | ref | GCCTGGGCTGCCATCTTG | GCCTGGGCTGCCATCTTG | MS | 19 | 0 | | MAAQAL | | QDGSPL |
| PTCHD3 | 10 | 27727230 | snp | A | G | N | 2343 | 767 | | * | | Q |
| PTEN | 10 | 89682883 | snp | C | T | NS | 1418 | 129 | | R | | * |
| PTGIR | 19 | 51819320 | ref | ATC | ATC | MS | 113 | 0 | | M | | E |
| PTGS2 | 1 | 184916031 | ref | GCGCGGGCGAGCATCGCAGCGGCGGGCAGGGCGCGGCGCG | GCGCGGGCGAGCATCGCAGCGGCGGGCAGGGCGCGGCGCG | MS | 134 | 0 | | MLARALLLCAVLAL | | RAAPCLLLCAVLAL |
| PTH1R | 3 | 46919250 | del | T |  | FS | 1564 | 453 | | F | | L |
| PTH2 | 19 | 54617517 | ref | ATGGGGACGGGCAGCGCGCTCAGGGCGCATCCAACACCAGC | ATGGGGACGGGCAGCGCGCTCAGGGCGCATCCAACACCAGC | N | 383 | 93 | | LLVLDAP* | | LERAARPH |
| PTPRN2 | 7 | 157384190 | snp | C | T | D | 1692 | 545 | |  | | GATTTTTCCTAACAA |
| PVRL2 | 19 | 50083289 | del | C |  | FS | 1820 | 476 | | P | | P |
| PYGL | 14 | 50448622 | snp | C | T | D | 1880 | 589 | |  | | ATGAGT |
| RAB11A | 15 | 63956902 | snp | C | T | NS | 347 | 73 | | R | | * |
| RAB24 | 5 | 176662880 | ref | GCGCTGCCCGCTCATG | GCGCTGCCCGCTCATG | MS | 369 | 0 | | MSGQRV | | HERAAV |
| RAB4A | 1 | 227488934 | del | A |  | FS | 318 | 36 | | K | | N |
| RAB5C | 17 | 37536044 | ref | ATTG | ATTG | MS | 200 | 0 | | MA | | QA |
| RABEP1 | 17 | 5205695 | snp | T | C | D | 1787 | 521 | |  | | GCGAGT |
| RANBP17 | 5 | 170273823 | snp | T | C | D | 850 | 278 | |  | | GCAAGT |
| RBM45 | 2 | 178697174 | ins |  | A | FS | 1235 | 381 | | A | | S |
| RBPJ | 4 | 26041180 | snp | C | T | NS | 1289 | 362 | | Q | | * |
| RECQL4 | 8 | 145709562 | ref | GCCACCACCACCCG | GCCACCACCACCCG | FS | 2328 | 765 | | RVVVA | | PGGGG |
| RETNLB | 3 | 109958693 | ins |  | GGATTAGG | FS | 127 | 9 | | I | | ILIP |
| REXO4 | 9 | 135261874 | ref | GCAGCAGCAGCAGGGCAGGACTGCTAGGCGTCGTCAC | GCAGCAGCAGCAGGGCAGGACTGCTAGGCGTCGTCAC | N | 1455 | 418 | | SDDA* | | TCCCC |
| RFTN1 | 3 | 16510378 | ref | ATTTCA | ATTTCA | MS | 282 | 0 | | MG | | WG |
| RGS12 | 4 | 3402235 | del | C |  | FS | 2196 | 641 | | P | | P |
| RGS21 | 1 | 190601685 | del | TT |  | FS | 441 | 89 | | F | | R |
| RGS9 | 17 | 60587447 | snp | C | T | NS | 491 | 127 | | R | | * |
| RHBG | 1 | 154620979 | ins |  | C | FS | 1310 | 424 | | R | | Q |
| RHCG | 15 | 87840777 | ref | ATGCT | ATGCT | MS | 24 | 0 | | MA | | RA |
| RIMKLB | 12 | 8793861 | snp | C | T | NS | 1574 | 104 | | R | | * |
| RIN2 | 20 | 19904399 | del | C |  | FS | 1766 | 576 | | P | | P |
| RIOK3 | 18 | 19298504 | del | A |  | FS | 1074 | 152 | | K | | R |
| RLBP1L2 | 6 | 123411576 | snp | G | A | D | 1544 | 225 | |  | | ATATTC |
| RNF112 | 17 | 19256678 | ins |  | C | FS | 570 | 123 | | P | | P |
| RNF12 | X | 73729156 | snp | G | A | NS | 927 | 239 | | R | | * |
| RNF133 | 7 | 122126205 | snp | C | T | MS | 238 | 0 | | M | | I |
| RNF169 | 11 | 74224905 | del | G |  | FS | 1622 | 536 | | G | | E |
| RNF20 | 9 | 103354312 | ins |  | G | FS | 1567 | 492 | | E | | G |
| RNF220 | 1 | 44650776 | snp | C | T | NS | 780 | 140 | | R | | * |
| RNF43 | 17 | 53790165 | del | C |  | FS | 3925 | 656 | | R | | R |
| RPH3AL | 17 | 63635 | ref | AGACACCTCAGCCCAGGCAGCTGGAGGGGCCTGCTGGAGCTGCGTCAGCAGCG | AGACACCTCAGCCCAGGCAGCTGGAGGGGCCTGCTGGAGCTGCGTCAGCAGCG | N | 1188 | 300 | | PAADAAPAGPSSCLG* | | PTQLQQAPPAAWAEVS |
| RPIA | 2 | 88817195 | snp | G | A | NS | 666 | 208 | | W | | * |
| RPL22 | 1 | 6180378 | del | T |  | FS | 82 | 12 | | K | | K |
| RREB1 | 6 | 7185801 | del | AAGA |  | FS | 4254 | 1313 | | KE | | R |
| RTN4RL1 | 17 | 1786538 | ref | CT | CT | N | 1344 | 441 | | * | | W |
| RTTN | 18 | 65848234 | del | TT |  | FS | 5804 | 1912 | | K | | K |
| RYR1 | 19 | 43626053 | del | G |  | FS | 416 | 95 | | G | | D |
| SAC | 1 | 166132511 | ins |  | A | FS | 869 | 228 | | F | | F |
| SAFB2 | 19 | 5538288 | del | G |  | FS | 3038 | 942 | | P | | P |
| SALL3 | 18 | 74857966 | del | G |  | FS | 3559 | 1186 | | G | | V |
| SAMD8 | 10 | 76580771 | del | T |  | FS | 566 | 159 | | F | | L |
| SARM1 | 17 | 23732426 | sub | TGGGT | CGTGG | FS | 615 | 182 | | SVA | | SVE |
| SBF1 | 22 | 49232435 | ref | CTCAGGCGTCCG | CTCAGGCGTCCG | N | 5778 | 1864 | | SDA* | | WTPE |
| SBNO1 | 12 | 122348658 | ins |  | T | FS | 3855 | 1285 | | K | | K |
| SCAMP1 | 5 | 77781610 | ins |  | A | FS | 888 | 243 | | N | | K |
| SCAMP3 | 1 | 153496757 | del | C |  | FS | 549 | 98 | | L | | L |
| SCAP | 3 | 47430346 | ref | CTCAG | CTCAG | N | 4091 | 1278 | | D* | | DE |
| SCHIP1 | 3 | 160965022 | del | C |  | FS | 226 | 53 | | P | | L |
| SCLT1 | 4 | 130083620 | ins |  | T | FS | 2118 | 537 | | Q | | Q |
| SCN1A | 2 | 166602816 | del | C |  | FS | 2645 | 875 | | V | | V |
| SCN3A | 2 | 165696013 | snp | G | A | NS | 3042 | 850 | | R | | * |
| SEC22B | 1 | 143823776 | snp | C | T | NS | 533 | 131 | | R | | * |
| SEC31A | 4 | 84004596 | del | T |  | FS | 1539 | 458 | | Q | | Q |
| SEMA3B | 3 | 50281752 | ref | CCAG | CCAG | FS | 318 | 25 | | PS | | PS |
| SEMA6B | 19 | 4509431 | ref | AGGGCCGGGCGGGGAGGGGACGCTCGCGGGGTCTGCATGGCGAGGGCCAGGCGACAGGAGGAGGTGACGCCT | AGGGCCGGGCGGGGAGGGGACGCTCGCGGGGTCTGCATGGCGAGGGCCAGGCGACAGGAGGAGGTGACGCCT | MS | 38 | 0 | | MQTPRASPPRPALLLLLLLLGGAH | | RRHLLLSPGPRHALLLLLLLGGAH |
| SENP1 | 12 | 46768863 | snp | G | A | NS | 812 | 122 | | R | | * |
| SETBP1 | 18 | 40897241 | ins |  | C | FS | 4214 | 1403 | | R | | Q |
| SFRS10 | 3 | 187138339 | ref | CATG | CATG | MS | 121 | 0 | | MS | | HS |
| SFRS12IP1 | 5 | 64059697 | ins |  | T | FS | 422 | 90 | | R | | K |
| SFRS2B | 11 | 94440551 | ins |  | C | FS | 849 | 171 | | R | | P |
| SFTPC | 8 | 22077004 | ins |  | GT | FS | 593 | 145 | | M | | V |
| SGEF | 3 | 155440986 | del | GTCA |  | FS | 2394 | 742 | | HL | | H |
| SGPL1 | 10 | 72306370 | snp | C | T | NS | 1722 | 504 | | R | | * |
| SGTA | 19 | 2708339 | ref | GTCACTC | GTCACTC | N | 1083 | 312 | | E* | | SD |
| SH2B2 | 7 | 101743754 | snp | C | G | FS | 1252 | 373 | | P | | K |
| SH3GLB1 | 1 | 86962647 | del | A |  | FS | 867 | 180 | | K | | R |
| SH3PXD2A | 10 | 105353385 | del | G |  | FS | 1634 | 498 | | P | | P |
| SIGLEC12 | 19 | 56696606 | sub | G | CT | FS | 248 | 64 | | R | | R |
| SIGLEC5 | 19 | 56825380 | ref | AGCAGCAGCAGGGGCAGCATGTCTCCATCCGCCA | AGCAGCAGCAGGGGCAGCATGTCTCCATCCGCCA | MS | 142 | 0 | | MLPLLLLPLLWG | | WRMETCWPLLWG |
| SKI | 1 | 2225860 | snp | C | T | NS | 1815 | 581 | | Q | | * |
| SKIV2L | 6 | 32036425 | del | G |  | FS | 862 | 157 | | G | | G |
| SLC15A4 | 12 | 127874440 | ref | TGCG | TGCG | MS | 53 | 0 | | ME | | LE |
| SLC16A5 | 17 | 70611870 | snp | G | A | D | 1651 | 454 | |  | | ATATGT |
| SLC17A5 | 6 | 74408316 | del | A |  | FS | 429 | 114 | | F | | F |
| SLC22A10 | 11 | 62814500 | snp | G | A | NS | 495 | 95 | | W | | * |
| SLC22A20 | 11 | 64738063 | del | C |  | FS | 179 | 48 | | H | | T |
| SLC22A9 | 11 | 62906256 | del | A |  | FS | 1272 | 334 | | K | | N |
| SLC23A2 | 20 | 4798575 | del | GG |  | FS | 1617 | 408 | | P | | P |
| SLC24A1 | 15 | 63704673 | del | C |  | FS | 1488 | 400 | | P | | Q |
| SLC25A13 | 7 | 95789194 | ref | CGGCCGCCATGA | CGGCCGCCATGA | MS | 137 | 0 | | MAAA | | SWRP |
| SLC27A3 | 1 | 152018489 | del | C |  | FS | 1997 | 644 | | H | | T |
| SLC29A2 | 11 | 65887513 | del | G |  | FS | 1568 | 446 | | S | | S |
| SLC30A10 | 1 | 218168402 | ref | CATCTC | CATCTC | MS | 211 | 0 | | MG | | EG |
| SLC38A10 | 17 | 76839672 | ins |  | T | FS | 2655 | 760 | | K | | K |
| SLC38A3 | 3 | 50226839 | ins |  | G | FS | 244 | 34 | | V | | G |
| SLC39A2 | 14 | 20539356 | del | C |  | FS | 895 | 236 | | L | | W |
| SLC41A3 | 3 | 127207964 | del | CAAA |  | FS | 1721 | 498 | | FC | | F |
| SLC43A3 | 11 | 56933222 | snp | C | T | D | 1653 | 456 | |  | | ATGAGT |
| SLC44A5 | 1 | 75457570 | del | C |  | FS | 1340 | 408 | | G | | G |
| SLC46A3 | 13 | 28190103 | del | A |  | FS | 383 | 10 | | F | | S |
| SLC4A3 | 2 | 220202361 | sub | A | CC | FS | 678 | 156 | | P | | P |
| SLC6A20 | 3 | 45812908 | ref | TCTCCATGGCCCCGGCC | TCTCCATGGCCCCGGCC | MS | 51 | 0 | | MEKARP | | GREARP |
| SLC8A1 | 2 | 40195885 | ref | TGATAGTTCCTTTAGAA | TGATAGTTCCTTTAGAA | N | 2940 | 972 | | F* | | LS |
| SLC9A3 | 5 | 537774 | del | C |  | FS | 801 | 263 | | V | | V |
| SLCO1C1 | 12 | 20766232 | ins |  | A | FS | 1371 | 334 | | I | | N |
| SLCO3A1 | 15 | 90260647 | ref | ACAT | ACAT | FS | 794 | 200 | | YY | | YM |
| SLCO5A1 | 8 | 70906804 | del | G |  | FS | 1273 | 219 | | P | | P |
| SLFN12 | 17 | 30773612 | del | A |  | FS | 924 | 182 | | V | | V |
| SMARCA1 | X | 128448769 | del | T |  | FS | 2216 | 707 | | Q | | Q |
| SMARCAD1 | 4 | 95390923 | snp | A | G | D | 884 | 269 | |  | | ATGACTTATTTCTGG |
| SMCHD1 | 18 | 2767819 | snp | C | T | NS | 5571 | 1794 | | R | | * |
| SMG5 | 1 | 154519074 | ref | TGTGGGGGGGCCTTGGCTCATGGT | TGTGGGGGGGCCTTGGCTCATGGT | MS | 150 | 0 | | MSQGPPTG | | TMSQGPPG |
| SMURF1 | 7 | 98486976 | ins |  | T | FS | 1065 | 248 | | Q | | Q |
| SNRPA1 | 15 | 99652904 | ref | ATCCTGCAG | ATCCTGCAG | MS | 77 | 0 | | MVK | | LVK |
| SON | 21 | 33870566 | del | A |  | FS | 7296 | 2415 | | R | | S |
| SORL1 | 11 | 120921265 | del | C |  | FS | 2090 | 656 | | H | | M |
| SOS2 | 14 | 49696158 | del | T |  | FS | 1689 | 530 | | E | | E |
| SP8 | 7 | 20792919 | snp | A | G | D | 108 | 6 | |  | | GCGAGT |
| SPDEF | 6 | 34620201 | ref | GCTGCCCATGCCGCTGC | GCTGCCCATGCCGCTGC | MS | 415 | 0 | | MGSASP | | AAAASP |
| SRD5A2 | 2 | 31659386 | ref | CTTCGCGA | CTTCGCGA | FS | 150 | 26 | | VAK | | VAK |
| SRPR | 11 | 125642303 | del | T |  | FS | 552 | 167 | | K | | K |
| SSPO | 7 | 149159194 | snp | C | T | NS | 15177 | 5059 | | R | | * |
| ST8SIA5 | 18 | 42513995 | ref | GCAGCCAT | GCAGCCAT | N | 1698 | 376 | | * | | C |
| STAMBPL1 | 10 | 90672132 | del | A |  | FS | 1636 | 404 | | K | | R |
| STAP2 | 19 | 4289741 | ref | AGAGGCCATGACGGAGCCAGGGTC | AGAGGCCATGACGGAGCCAGGGTC | MS | 97 | 0 | | MASALRPP | | DPGALRPP |
| STMN3 | 20 | 61746008 | snp | A | G | D | 196 | 38 | |  | | GCGAGT |
| STON1 | 2 | 48661862 | del | T |  | FS | 596 | 195 | | F | | S |
| STON1-GTF2A1L | 2 | 48661862 | del | T |  | FS | 700 | 195 | | F | | S |
| STX6 | 1 | 179258429 | ref | CATGGCG | CATGGCG | MS | 237 | 0 | | MSM | | RSM |
| SUCLA2 | 13 | 47473402 | ref | CCATTTCTGA | CCATTTCTGA | MS | 57 | 0 | | MAAS | | SAAS |
| SUMF1 | 3 | 4483917 | ref | GGGCGCAGCCATGTTGTCCCGCGGGCCA | GGGCGCAGCCATGTTGTCCCGCGGGCCA | MS | 25 | 0 | | MAAPALGLVC | | WPAGALGLVC |
| SUPT3H | 6 | 45090569 | snp | G | A | NS | 380 | 103 | | R | | * |
| SYCP1 | 1 | 115270664 | del | A |  | FS | 2092 | 627 | | K | | K |
| SYCP2 | 20 | 57885921 | del | T |  | FS | 3201 | 1020 | | T | | T |
| SYMPK | 19 | 51018519 | snp | G | A | NS | 2894 | 883 | | R | | * |
| SYNE1 | 6 | 152617757 | snp | G | A | NS | 20021 | 6473 | | Q | | * |
| SYT6 | 1 | 114437947 | snp | C | A | NS | 1510 | 420 | | G | | * |
| TAS2R46 | 12 | 11105411 | snp | C | T | NS | 748 | 249 | | W | | * |
| TAS2R50 | 12 | 11030706 | del | A |  | FS | 71 | 6 | | I | | I |
| TBC1D16 | 17 | 75529247 | ref | CCGACCTATCTGC | CCGACCTATCTGC | N | 2412 | 765 | | RR* | | LGR |
| TBC1D29 | 17 | 25911259 | snp | G | A | D | 161 | 4 | |  | | TGTTCCTCCCATCAA |
| TBX10 | 11 | 67158333 | del | G |  | FS | 538 | 150 | | H | | T |
| TCEAL6 | X | 101282439 | ins |  | G | FS | 681 | 173 | | A | | A |
| TCP1 | 6 | 160126995 | del | T |  | FS | 537 | 100 | | L | | L |
| TCTEX1D1 | 1 | 67014674 | snp | G | A | D | 465 | 112 | |  | | ATACGT |
| TCTN1 | 12 | 109542144 | snp | G | A | D | 449 | 113 | |  | | ATAAGT |
| TDRD10 | 1 | 152783470 | snp | G | T | D | 1489 | 217 | |  | | TTTTGCACCCCACAT |
| TELO2 | 16 | 1490626 | snp | C | T | NS | 1427 | 402 | | R | | * |
| TFAM | 10 | 59818584 | del | A |  | FS | 572 | 146 | | K | | K |
| TFAP2B | 6 | 50899215 | del | C |  | FS | 384 | 72 | | P | | P |
| TFR2 | 7 | 100077065 | ref | CATGCTTGTGT | CATGCTTGTGT | MS | 41 | 0 | | MERL | | TERL |
| TGFB2 | 1 | 216676001 | ins |  | A | FS | 1002 | 273 | | N | | K |
| TGFBR2 | 3 | 30666884 | del | A |  | FS | 764 | 127 | | K | | S |
| THAP7 | 22 | 19684160 | ref | GCAGCCCCTCAGGCCAT | GCAGCCCCTCAGGCCAT | N | 1125 | 307 | | MA* | | RGC |
| THRA | 17 | 35498280 | snp | T | C | D | 1465 | 327 | |  | | GCACCT |
| TICAM1 | 19 | 4767227 | ref | GGTGGTCAGGCAAGGACACGCGGTCATTCT | GGTGGTCAGGCAAGGACACGCGGTCATTCT | N | 2194 | 710 | | AE* | | ATT |
| TIMD4 | 5 | 156309272 | ref | TAGACTCA | TAGACTCA | NS | 776 | 239 | | AEST | | V* |
| TLL2 | 10 | 98263419 | ref | TCGCCCGGGGCATGGTGGCGCGGGGC | TCGCCCGGGGCATGGTGGCGCGGGGC | MS | 226 | 0 | | MPRATALGA | | APRHPALGA |
| TLN1 | 9 | 35714622 | snp | G | A | NS | 546 | 152 | | R | | * |
| TM4SF4 | 3 | 150675383 | del | G |  | FS | 260 | 9 | | L | | L |
| TM6SF1 | 15 | 81581760 | snp | T | C | D | 534 | 160 | |  | | GCAAGA |
| TMEM120A | 7 | 75461812 | ref | CGGGGGCTGCATGGCTGCAG | CGGGGGCTGCATGGCTGCAG | MS | 65 | 0 | | MQPPPPG | | LQPCPPG |
| TMEM160 | 19 | 52241077 | ref | GTGTCGCTCACTCGGGTGGCGGCCGACCCGCCTCATCAG | GTGTCGCTCACTCGGGTGGCGGCCGACCCGCCTCATCAG | N | 545 | 178 | | PDEAGRPPPE* | | RRVGRHPSERH |
| TMEM216 | 11 | 60922307 | ins |  | A | FS | 683 | 82 | | S | | K |
| TMEM41A | 3 | 186699420 | ref | AGGAGGCCGAGAAGCGGGCGCATGTCGGCTCCGCACCCCGGCCCGCGGGGCAGCCGA | AGGAGGCCGAGAAGCGGGCGCATGTCGGCTCCGCACCCCGGCCCGCGGGGCAGCCGA | MS | 53 | 0 | | MRPLLGLLLVFAGCTFALY | | SAAPRAGVLVFAGCTFALY |
| TMEM66 | 8 | 30060006 | ref | TGGCGCTCGATGAAGATGGCGCCGG | TGGCGCTCGATGAAGATGGCGCCGG | MS | 184 | 0 | | MAAACGPGA | | LAAACGPGA |
| TMEM9B | 11 | 8942435 | ref | TCGCTGGGGGCCCAGCGGTCCCACAGCCCG | TCGCTGGGGGCCCAGCGGTCCCACAGCCCG | MS | 129 | 0 | | MATLWGGLLR | | LATLWGGLLR |
| TMF1 | 3 | 69160064 | snp | A | G | D | 2910 | 887 | |  | | GCATTT |
| TMOD2 | 15 | 49887934 | snp | C | T | NS | 1216 | 349 | | R | | * |
| TMPRSS11A | 4 | 68511703 | snp | C | T | MS | 123 | 0 | | M | | I |
| TMPRSS12 | 12 | 49538987 | del | A |  | FS | 583 | 178 | | K | | K |
| TNRC6B | 22 | 39049066 | del | G |  | FS | 5258 | 1682 | | W | | W |
| TNXB | 6 | 32165022 | snp | G | A | NS | 2529 | 775 | | R | | * |
| TPR | 1 | 184598040 | snp | A | G | D | 1085 | 262 | |  | | GCATAT |
| TPTE2 | 13 | 18895187 | ref | AACATCATTGGAAGTCATTTCTCGCC | AACATCATTGGAAGTCATTTCTCGCC | N | 1768 | 519 | | GEK* | | SNDV |
| TRIM59 | 3 | 161639070 | del | T |  | FS | 789 | 198 | | K | | K |
| TRIM66 | 11 | 8603229 | del | G |  | FS | 2317 | 772 | | A | | A |
| TRMT6 | 20 | 5872815 | ins |  | T | FS | 572 | 150 | | K | | K |
| TROVE2 | 1 | 191304807 | del | A |  | FS | 417 | 0 | | M | | W |
| TRPA1 | 8 | 73128613 | ins |  | C | FS | 1747 | 524 | | G | | G |
| TRPC3 | 4 | 123073572 | ins |  | C | FS | 495 | 23 | | G | | G |
| TRPC6 | 11 | 100959443 | ref | TGGCGGGAACGCCCGACTGGCCTGGGCCCCGCTCCCGGG | TGGCGGGAACGCCCGACTGGCCTGGGCCCCGCTCCCGGG | MS | 425 | 0 | | MSQSPAFGPRRGS | | LSQSPAFGPRRGS |
| TRPM1 | 15 | 29156414 | snp | A | G | MS | 129 | 0 | | M | | T |
| TRPV1 | 17 | 3423920 | ins |  | G | FS | 2133 | 619 | | A | | A |
| TSHZ2 | 20 | 51305857 | del | C |  | FS | 3409 | 817 | | P | | P |
| TSPAN10 | 17 | 77225342 | del | AACT |  | FS | 771 | 227 | | NC | | A |
| TSPAN16 | 19 | 11298478 | ins |  | G | FS | 846 | 232 | | S | | V |
| TSSK6 | 19 | 19486389 | ref | AGGAATGGCTGGAACCACCCGGCTTCTAGCCGGAGTCCCCGGCGCGCAGCCAGCAGTTGCGCG | AGGAATGGCTGGAACCACCCGGCTTCTAGCCGGAGTCCCCGGCGCGCAGCCAGCAGTTGCGCG | N | 1017 | 261 | | ARNCWLRAGDSG* | | GTPARSRVVPAIP |
| TTC3 | 21 | 37446119 | del | A |  | FS | 3729 | 777 | | K | | K |
| TTC39B | 9 | 15297112 | ref | AAGAGCGCCATATT | AAGAGCGCCATATT | MS | 121 | 0 | | MALLG | | NMALG |
| TTC7B | 14 | 90322366 | del | G |  | FS | 301 | 59 | | H | | H |
| TTK | 6 | 80808623 | del | A |  | FS | 2633 | 853 | | R | | G |
| TTN | 2 | 179252569 | snp | C | T | D | 30156 | 9977 | |  | | ATACAC |
| TUBA4A | 2 | 219825096 | snp | G | A | NS | 160 | 34 | | Q | | * |
| UBE2NL | X | 142795133 | snp | T | G | NS | 295 | 88 | | L | | * |
| UBE2U | 1 | 64453185 | del | T |  | FS | 683 | 146 | | F | | S |
| UBR4 | 1 | 19409317 | ref | GCTCGTCGCCATCTTCCGTCGTACTACTGCG | GCTCGTCGCCATCTTCCGTCGTACTACTGCG | MS | 4 | 0 | | MATSGGEEAAA | | EDGDGGEEAAA |
| UBR5 | 8 | 103343453 | del | A |  | FS | 7739 | 2568 | | A | | A |
| UBXN8 | 8 | 30740386 | ins |  | T | FS | 643 | 208 | | L | | F |
| UCK1 | 9 | 133390239 | ref | GCAGCCCCTCAGTGG | GCAGCCCCTCAGTGG | N | 921 | 275 | | PH* | | PGC |
| UGT1A3 | 2 | 234303028 | del | T |  | FS | 517 | 172 | | L | | * |
| UGT2B7 | 4 | 69998925 | ref | AT | AT | FS | 814 | 266 | | PH | | PS |
| UHRF1 | 19 | 4905679 | ins |  | C | FS | 2307 | 671 | | A | | A |
| UHRF1BP1 | 6 | 34934107 | del | T |  | FS | 2230 | 665 | | L | | C |
| UNC93A | 6 | 167631548 | snp | G | C | D | 702 | 208 | |  | | CTACGA |
| UROS | 10 | 127467424 | ref | ACTCA | ACTCA | N | 991 | 265 | | * | | S |
| USH1G | 17 | 70430735 | ref | GGGCTGCCCGGTGGTACTGGTCGTTCATGGCGCCCGAAGT | GGGCTGCCCGGTGGTACTGGTCGTTCATGGCGCCCGAAGT | MS | 183 | 0 | | MNDQYHRAARDGYL | | TSGAMNDQYRDGYL |
| USP10 | 16 | 83366333 | snp | T | C | D | 2351 | 736 | |  | | GCGAGT |
| USP35 | 11 | 77602454 | del | G |  | FS | 3250 | 1001 | | G | | A |
| USP42 | 7 | 6162967 | del | A |  | FS | 3822 | 1233 | | K | | K |
| USP45 | 6 | 100023186 | ins |  | G | FS | 1495 | 320 | | F | | F |
| USP50 | 15 | 48623092 | del | T |  | FS | 632 | 150 | | L | | L |
| VMO1 | 17 | 4636364 | ref | TTGGCTCCTGCGCCCCGCTCCATCCT | TTGGCTCCTGCGCCCCGCTCCATCCT | MS | 87 | 0 | | MERGAGAKL | | RMERGAGAL |
| VN1R5 | 1 | 245486131 | ref | T | T | NS | 135 | 45 | | Q | | * |
| VPS11 | 11 | 118445151 | ins |  | C | FS | 265 | 74 | | L | | P |
| VPS13A | 9 | 79010784 | snp | G | T | NS | 614 | 118 | | E | | * |
| VPS13B | 8 | 100202881 | snp | T | G | NS | 1349 | 412 | | Y | | * |
| VPS13C | 15 | 60139827 | ref | CGGTTCAGCAAGTCCGCGACCACCGACTCCAGCACCATGGTGGCGCT | CGGTTCAGCAAGTCCGCGACCACCGACTCCAGCACCATGGTGGCGCT | MS | 74 | 0 | | MVLESVVADLLNRFLG | | SATMVLESVVADFFLG |
| WBP1 | 2 | 74541057 | del | T |  | FS | 704 | 183 | | P | | P |
| WDFY3 | 4 | 85935151 | ins |  | A | FS | 3580 | 1057 | | L | | L |
| WDR37 | 10 | 1139693 | del | G |  | FS | 1051 | 292 | | G | | G |
| WDR5 | 9 | 136009425 | ins |  | C | FS | 734 | 216 | | V | | R |
| WDR63 | 1 | 85360075 | snp | C | T | NS | 2155 | 657 | | R | | * |
| WDR67 | 8 | 124223877 | snp | G | T | D | 2925 | 945 | |  | | TTAAAA |
| WDR68 | 17 | 59014626 | del | G |  | FS | 776 | 186 | | G | | V |
| WDR82 | 3 | 52287413 | ref | TCATGGCGGCGGCT | TCATGGCGGCGGCT | MS | 282 | 0 | | MKLTD | | SQLTD |
| WNT16 | 7 | 120759120 | del | G |  | FS | 756 | 166 | | G | | A |
| XKR5 | 8 | 6660787 | del | A |  | FS | 891 | 289 | | S | | S |
| XPA | 9 | 99499382 | ref | CGGCCGCCGCCATCTC | CGGCCGCCGCCATCTC | MS | 117 | 0 | | MAAADG | | EMAADG |
| XRCC4 | 5 | 82684698 | snp | G | A | D | 1020 | 297 | |  | | TGATTTTCTTTTCAA |
| XRN1 | 3 | 143605304 | ins |  | A | FS | 2071 | 668 | | F | | F |
| XYLT2 | 17 | 45788965 | del | C |  | FS | 1686 | 525 | | Y | | Y |
| ZAN | 7 | 100223498 | del | CTTCAGCTACCGCTTGCAAGGCCGCATGACCTATG |  | FS | 7196 | 2343 | | GFSYRLQGRMTYV | | G |
| ZBTB46 | 20 | 61848725 | ref | CCTA | CCTA | N | 1968 | 589 | | * | | R |
| ZBTB7C | 18 | 43809620 | ref | GGACCAGCC | GGACCAGCC | N | 1875 | 619 | | * | | Y |
| ZC3H18 | 16 | 87218649 | ins |  | C | FS | 2184 | 679 | | R | | Q |
| ZCCHC3 | 20 | 226685 | ref | G | G | FS | 482 | 152 | | E | | E |
| ZCCHC6 | 9 | 88145749 | snp | A | T | NS | 1339 | 374 | | C | | * |
| ZCRB1 | 12 | 40993237 | ins |  | T | FS | 761 | 184 | | W | | M |
| ZDBF2 | 2 | 206880776 | del | A |  | FS | 3529 | 1093 | | I | | * |
| ZDHHC11 | 5 | 901763 | del | C |  | FS | 617 | 77 | | G | | G |
| ZFC3H1 | 12 | 70325052 | del | T |  | FS | 1508 | 383 | | K | | K |
| ZFHX2 | 14 | 23063226 | del | C |  | FS | 6165 | 2055 | | G | | G |
| ZFHX4 | 8 | 77929659 | snp | C | T | NS | 8199 | 2604 | | Q | | * |
| ZFP106 | 15 | 40530255 | del | A |  | FS | 1771 | 478 | | N | | N |
| ZFP91 | 11 | 58134997 | snp | G | A | D | 785 | 205 | |  | | TTTACTTATTTTTAA |
| ZFP91-CNTF | 11 | 58134997 | snp | G | A | D | 785 | 205 | |  | | TTTACTTATTTTTAA |
| ZFR | 5 | 32439917 | del | T |  | FS | 1153 | 357 | | K | | K |
| ZMYM4 | 1 | 35653747 | ins |  | T | FS | 4234 | 1384 | | F | | F |
| ZNF117 | 7 | 64076101 | snp | G | A | NS | 2566 | 427 | | R | | * |
| ZNF124 | 1 | 245386531 | del | T |  | FS | 967 | 276 | | T | | L |
| ZNF148 | 3 | 126433874 | del | TT |  | FS | 2869 | 794 | | * | | ? |
| ZNF16 | 8 | 146127367 | snp | G | A | NS | 1709 | 536 | | R | | * |
| ZNF174 | 16 | 3398560 | del | C |  | FS | 1449 | 288 | | L | | * |
| ZNF193 | 6 | 28308878 | snp | C | T | NS | 1276 | 376 | | R | | * |
| ZNF211 | 19 | 62845276 | snp | T | A | NS | 1741 | 536 | | Y | | * |
| ZNF227 | 19 | 49431538 | del | AG |  | FS | 1320 | 371 | | RV | | S |
| ZNF235 | 19 | 49499396 | ref | TTTTTCCCCTCCTC | TTTTTCCCCTCCTC | MS | 95 | 0 | | MTKFQ | | VTKFQ |
| ZNF274 | 19 | 63410173 | ins |  | G | FS | 895 | 145 | | A | | G |
| ZNF318 | 6 | 43433062 | snp | G | A | NS | 1044 | 322 | | R | | * |
| ZNF425 | 7 | 148454233 | ref | GCCATGGCGG | GCCATGGCGG | MS | 73 | 0 | | MAEP | | PPEP |
| ZNF428 | 19 | 48803588 | ref | TCCCCGTATGGGGGCTCTGCCTACACCTCACCCCGGGCA | TCCCCGTATGGGGGCTCTGCCTACACCTCACCCCGGGCA | N | 975 | 182 | | HARGEV* | | HRAPIRG |
| ZNF468 | 19 | 58036743 | del | T |  | FS | 765 | 204 | | K | | N |
| ZNF493 | 19 | 21397735 | ins |  | T | FS | 543 | 144 | | Y | | Y |
| ZNF516 | 18 | 72219951 | del | G |  | FS | 3340 | 1035 | | P | | P |
| ZNF540 | 19 | 42783845 | ins |  | C | FS | 563 | 77 | | G | | R |
| ZNF595 | 4 | 77313 | ins |  | A | FS | 2075 | 639 | | R | | R |
| ZNF620 | 3 | 40532441 | del | G |  | FS | 501 | 117 | | G | | A |
| ZNF768 | 16 | 30445232 | ref | CATCC | CATCC | MS | 176 | 0 | | ME | | GE |
| ZNF806 | 2 | 132792588 | del | A |  | FS | 1579 | 526 | | N | | M |
| ZNF90 | 19 | 20077081 | del | A |  | FS | 272 | 90 | | K | | N |
| ZNRF1 | 16 | 73697937 | snp | A | G | N | 1338 | 227 | | * | | W |
| ZRANB3 | 2 | 135701894 | del | T |  | FS | 2231 | 704 | | P | | P |

Supplementary Table 2: Gene mutations identified in the G089 sample (except missense mutations). Type includes SNP, deletions (del), insertions (ins), reference (ref) and no call (-). Impact includes misstart (MS), frameshift (FS), disrupt (D), nonsense (NS) and nonstop (N). Position is defined based on the start of the small variant (SV) call. Additional information on the output from complete genomics can be found on (<http://www.completegenomics.com/FAQs/Variant-Calls-SNPs-and-Small-Indels/>)

| Symbol | Chr# | Position | Type | ref | | call | Impact | | Nucl  POS | Prot  POS | Ref | | Sample |  |
| --- | --- | --- | --- | --- | --- | --- | --- | --- | --- | --- | --- | --- | --- | --- |
| AADACL3 | 1 | 12698930 | snp | A | | T | MS | | 226 | 0 | M | | L |  |
| ABCB9 | 12 | 122010730 | ref | CGCATCCTGC | | CGCATCCTGC | MS | | 310 | 0 | MRLW | | AGLW |  |
| ABCC6 | 16 | 16224770 | ref | AGGGCTCAGCAGGCGCGGCCATCGGCGC | | AGGGCTCAGCAGGCGCGGCCATCGGCGC | MS | | 23 | 0 | MAAPAEPCAG | | APMAAPAGAG |  |
| ABHD14B | 3 | 51980724 | ref | ATGC | | ATGC | MS | | 272 | 0 | MA | | AA |  |
| ABI1 | 10 | 27077503 | ref | AT | | AT | N | | 1525 | 452 | * | | Y |  |
| ABI3BP | 3 | 102194938 | snp | T | | C | MS | | 65 | 0 | M | | V |  |
| ABLIM2 | 4 | 8211302 | ref | CCACCTGCACTCATCTCAGCAGCC | | CCACCTGCACTCATCTCAGCAGCC | MS | | 143 | 0 | MSAVSQPQ | | GC* |  |
| ABO | 9 | 135122729 | ref |  | |  | FS | | 286 | 87 | T | | P |  |
| ACADL | 2 | 210798201 | ref | GTAGGGACCCTCGGAGAAGGCGTGCGGCCATGTCC | | GTAGGGACCCTCGGAGAAGGCGTGCGGCCATGTCC | MS | | 160 | 0 | MAARLLRGSLRV | | GHGRTPSPRVRV |  |
| ACBD3 | 1 | 224440997 | ref | ATC | | ATC | MS | | 47 | 0 | M | | E |  |
| ACSM4 | 12 | 7366347 | snp | C | | T | NS | | 1068 | 356 | Q | | * |  |
| ADAM8 | 10 | 134926550 | ref | GGTCTA | | GGTCTA | N | | 2619 | 856 | * | | T |  |
| ADSS | 1 | 242681737 | ref | GCCATG | | GCCATG | MS | | 294 | 0 | MA | | HG |  |
| ADSSL1 | 14 | 104267274 | snp | A | | C | MS | | 2 | 0 | M | | L |  |
| AGAP2 | 12 | 56406574 | ref | GATGGGGGTGTCTCTCCCGCTGGGCAACTATACCAGCGCAACCGG | | GATGGGGGTGTCTCTCCCGCTGGGCAACTATACCAGCGCAACCGG | N | | 2579 | 831 | PVALV* | | GRDTPI |  |
| AHDC1 | 1 | 27751203 | ref | TCACACGCATC | | TCACACGCATC | MS | | 969 | 0 | MRVK | | DACE |  |
| AIDA | 1 | 220952280 | ref | ATGGCCGGTCCCC | | ATGGCCGGTCCCC | MS | | 205 | 0 | MSEVT | | GSEVT |  |
| AKTIP | 16 | 52084123 | del | TTCTC | |  | FS | | 959 | 249 | RE | | K |  |
| ALDH3B1 | 11 | 67542641 | ins |  | | C | FS | | 306 | 77 | L | | P |  |
| ALG8 | 11 | 77528278 | ref | CCATTGCTGC | | CCATTGCTGC | MS | | 65 | 0 | MAAL | | AAAL |  |
| ALMS1 | 2 | 73528735 | ins |  | | CTC | FS | | 1687 | 525 | P | | LL |  |
| AMFR | 16 | 54954317 | ref | AGCGCT | | AGCGCT | N | | 2135 | 643 | * | | S |  |
| ANKRD23 | 2 | 96868993 | ref | GCGGGGGGCGGTGCTGCGGTCAG | | GCGGGGGGCGGTGCTGCGGTCAG | N | | 943 | 304 | C* | | CR |  |
| ANKRD6 | 6 | 90372508 | ref | TTAA | | TTAA | NS | | 723 | 127 | VK | | I* |  |
| ANO8 | 19 | 17295312 | ref | AGGGCGGGTAGAGCTAATGCCAGCCGCT | | AGGGCGGGTAGAGCTAATGCCAGCCGCT | N | | 3843 | 1228 | SGWH* | | SSTRP |  |
| ANP32A | 15 | 66900131 | ref | TGCCCATCTCCATCTCTCGCGCT | | TGCCCATCTCCATCTCTCGCGCT | MS | | 133 | 0 | MEMGRRIH | | SARDGRIH |  |
| APOL4 | 22 | 34917793 | ref |  | |  | FS | | 745 | 107 | E | | S |  |
| AQP7 | 9 | 33376398 | sub | CA | | TG | D | | 577 | 135 |  | | GCAAGT |  |
| ARHGAP19 | 10 | 99042373 | ref | TCTTCG | | TCTTCG | MS | | 29 | 0 | MA | | LA |  |
| ARL15 | 5 | 53642064 | ref | ATCCG | | ATCCG | MS | | 94 | 0 | MS | | RS |  |
| ASB15 | 7 | 123054422 | del | G | |  | FS | | 762 | 240 | D | | M |  |
| ATG9B | 7 | 150344836 | ins |  | | C | FS | | 2370 | 764 | E | | E |  |
| ATP8B3 | 19 | 1747879 | snp | T | | C | D | | 1817 | 527 |  | | CCCCGCCACCCCCGG |  |
| ATPBD3 | 19 | 56293667 | ref | CGCTAGAAGGT | | CGCTAGAAGGT | N | | 1059 | 346 | TF* | | LLA |  |
| ATPBD4 | 15 | 33451638 | ref | CTTTTCAAAAATTATATATATAATTAGATGTTCTGTAGT | | CTTTTCAAAAATTATATATATAATTAGATGTTCTGTAGT | N | | 791 | 256 | NYRTSNYIYNF* | | TEHLIIYIIFEK |  |
| BCL7C | 16 | 30812759 | ref | CGGCCATGCTGGCGGGG | | CGGCCATGCTGGCGGGG | MS | | 134 | 0 | MAGRTV | | PRRRTV |  |
| BMF | 15 | 38171269 | ref | CAGCCCACCCTCACCTAG | | CAGCCCACCCTCACCTAG | N | | 484 | 161 | PR* | | RGL |  |
| BRF2 | 8 | 37826457 | ref | ATCTCACA | | ATCTCACA | MS | | 110 | 0 | MPG | | WPG |  |
| C10orf118 | 10 | 115913011 | ref | TGACATG | | TGACATG | MS | | 652 | 0 | MSE | | HVE |  |
| C10orf67 | 10 | 23673553 | ref | CCTACGCGCGCAGCAGAC | | CCTACGCGCGCAGCAGAC | NS | | 207 | 46 | VCCARR | | GLLRA* |  |
| C10orf79 | 10 | 105879876 | ref | GCCAGCGTTTTTACATTTG | | GCCAGCGTTTTTACATTTG | N | | 5105 | 1663 | QM* | | NAG |  |
| C10orf84 | 10 | 120060355 | ref | AAGTTA | | AAGTTA | N | | 945 | 233 | * | | L |  |
| C10orf88 | 10 | 124703680 | ref | CCATTCCGCCGCC | | CCATTCCGCCGCC | MS | | 225 | 0 | METRT | | GETRT |  |
| C11orf40 | 11 | 4549286 | ins |  | | AG | FS | | 596 | 198 | N | | NS |  |
| C11orf59 | 11 | 71491916 | ref | ATGGCCG | | ATGGCCG | MS | | 52 | 0 | MGC | | RGC |  |
| C13orf18 | 13 | 45815511 | ref | TCAGGGGCATCATGTTGCTGCAGAG | | TCAGGGGCATCATGTTGCTGCAGAG | NS | | 2578 | 657 | ASAAT* | | AT* |  |
| C14orf105 | 14 | 57018132 | snp | T | | C | D | | 529 | 130 |  | | TGTTGTCTTTCATGG |  |
| C14orf181 | 14 | 68332260 | ref | ACGT | | ACGT | N | | 679 | 166 | * | | C |  |
| C14orf48 | 14 | 93534241 | snp | G | | A | MS | | 419 | 0 | M | | I |  |
| C15orf24 | 15 | 32163825 | ref | ACTACCTCCTTTTGCCAGCCCC | | ACTACCTCCTTTTGCCAGCCCC | N | | 721 | 236 | GAGKRR* | | GLAKGGS |  |
| C15orf52 | 15 | 38414626 | ref | CTCCCCAGCTCCCAGGAGCTGTGTTCAGCAT | | CTCCCCAGCTCCCAGGAGCTGTGTTCAGCAT | N | | 1614 | 532 | RC* | | SGE |  |
| C15orf57 | 15 | 38644261 | ref | AGCCCCGCATGGCCCC | | AGCCCCGCATGGCCCC | MS | | 273 | 0 | MRGSGL | | GAMPGL |  |
| C16orf35 | 16 | 78773 | ins |  | | G | FS | | 1605 | 309 | S | | S |  |
| C16orf71 | 16 | 4737022 | del | C | |  | FS | | 1737 | 425 | L | | Y |  |
| C17orf100 | 17 | 6496272 | ins |  | | G | FS | | 491 | 105 | A | | G |  |
| C19orf64 | 19 | 3543543 | del | C | |  | FS | | 1101 | 337 | T | | T |  |
| C1orf173 | 1 | 74911772 | ref | CGGGGTGAGAATGGCTCATTTTTG | | CGGGGTGAGAATGGCTCATTTTTG | MS | | 219 | 0 | MSHSHPAG | | QK* |  |
| C1orf183 | 1 | 112099847 | ref | GTTGCCTCATCTTTCCTCTCCTCCCTCTTG | | GTTGCCTCATCTTTCCTCTCCTCCCTCTTG | MS | | 85 | 0 | MRQLMSMKEV | | QEGVMSMKEV |  |
| C20orf12 | 20 | 18394000 | ref | ATT | | ATT | MS | | 209 | 0 | M | | K |  |
| C20orf96 | 20 | 206969 | snp | C | | T | D | | 703 | 188 |  | | ATAAGA |  |
| C21orf62 | 21 | 33088060 | ins |  | | T | FS | | 792 | 180 | L | | L |  |
| C22orf33 | 22 | 35728055 | ref | ATGCCCAGA | | ATGCCCAGA | MS | | 344 | 0 | MDP | | SDP |  |
| C22orf39 | 22 | 17815194 | ref | CCGCTGCCGTCCGCCATGTCTGG | | CCGCTGCCGTCCGCCATGTCTGG | MS | | 9 | 0 | MADGSGWQ | | PDMADGWQ |  |
| C2orf24 | 2 | 219749787 | ref | GGTCCATCG | | GGTCCATCG | MS | | 137 | 0 | MDL | | RWM |  |
| C2orf83 | 2 | 228184383 | snp | C | | T | NS | | 509 | 140 | W | | * |  |
| C4orf17 | 4 | 100662804 | ref | TCC | | TCC | FS | | 575 | 84 | P | | S |  |
| C5orf20 | 5 | 134810348 | snp | T | | A | NS | | 588 | 116 | R | | * |  |
| C5orf33 | 5 | 36261462 | ref | CAGCAGCATTGTGCCATCACCTA | | CAGCAGCATTGTGCCATCACCTA | MS | | 250 | 0 | MLLAASKV | | VMAAASKV |  |
| C5orf41 | 5 | 172468427 | snp | G | | A | D | | 1721 | 472 |  | | ATAAGA |  |
| C6orf132 | 6 | 42202530 | snp | C | | T | NS | | 813 | 93 | W | | * |  |
| C6orf150 | 6 | 74218622 | ref | CATGGC | | CATGGC | MS | | 139 | 0 | MQ | | AQ |  |
| C9 | 5 | 39400318 | ref | GACATGCTGC | | GACATGCTGC | MS | | 89 | 0 | MSAC | | AAAC |  |
| C9orf102 | 9 | 97678108 | snp | A | | G | MS | | 388 | 0 | M | | V |  |
| C9orf153 | 9 | 88032066 | ref | TTTAT | | TTTAT | N | | 383 | 100 | K* | | NK |  |
| C9orf5 | 9 | 110921994 | ref | GGGCCGCCGCCGTCGGCCATCGTTCCTCCGCCACAGCC | | GGGCCGCCGCCGTCGGCCATCGTTCCTCCGCCACAGCC | MS | | 32 | 0 | MADGGGPKDAPSL | | GCGGGTIKDAPSL |  |
| C9orf50 | 9 | 131422672 | ref | ATGCTTG | | ATGCTTG | MS | | 202 | 0 | MFW | | QFW |  |
| CAB39L | 13 | 48782945 | ref | GCTCTTCAAGGGGCCGTTTTCTTC | | GCTCTTCAAGGGGCCGTTTTCTTC | N | | 1493 | 331 | LKKTAP* | | LNGPLKS |  |
| CACNB1 | 17 | 34607273 | ref | TGGAGAG | | TGGAGAG | MS | | 153 | 0 | MVQ | | LVQ |  |
| CASP12 | 11 | 104266309 | snp | T | | C | D | | 1072 | 0 |  | | GTAAGT |  |
| CATSPER2 | 15 | 41710189 | ref | TTTACTTGT | | TTTACTTGT | N | | 1800 | 528 | DK* | | ASK |  |
| CBFA2T3 | 16 | 87570710 | ref | CGGCATG | | CGGCATG | MS | | 186 | 0 | MPA | | HAA |  |
| CBX4 | 17 | 75422340 | ref | GACGCCCTCCGGCTACACCGTCACGTACTC | | GACGCCCTCCGGCTACACCGTCACGTACTC | NS | | 1828 | 555 | EYVTV* | | V* |  |
| CCDC108 | 2 | 219611501 | snp | T | | G | MS | | 124 | 0 | M | | L |  |
| CCDC53 | 12 | 100943940 | ins |  | | G | FS | | 600 | 147 | P | | P |  |
| CCDC6 | 10 | 61336186 | ref | ATGGCCGCGGCGGG | | ATGGCCGCGGCGGG | MS | | 636 | 0 | MADSA | | PADSA |  |
| CCHCR1 | 6 | 31232827 | snp | C | | T | NS | | 422 | 77 | W | | * |  |
| CCNC | 6 | 100098148 | ref | GTTTTAAGATTGGCTGTAGCTAGAG | | GTTTTAAGATTGGCTGTAGCTAGAG | NS | | 1117 | 276 | NSSYSQS* | | NSYSQS* |  |
| CD164 | 6 | 109810273 | ref | TCGTG | | TCGTG | MS | | 81 | 0 | MS | | LS |  |
| CD200R1L | 3 | 114028600 | del | T | |  | FS | | 833 | 202 | H | | P |  |
| CD63 | 12 | 54408389 | ref | CATGGCT | | CATGGCT | MS | | 185 | 0 | MAV | | SAV |  |
| CDCP2 | 1 | 54377907 | ins |  | | C | FS | | 2071 | 407 | P | | P |  |
| CDKN1C | 11 | 2863293 | ref | ATGGCCCG | | ATGGCCCG | MS | | 260 | 0 | MSD | | RSD |  |
| CDSN | 6 | 31191779 | del | CTT | |  | FS | | 1615 | 529 | * | | ? |  |
| CENPB | 20 | 3715129 | ref | TCCCG | | TCCCG | MS | | 207 | 0 | MG | | LG |  |
| CEP110 | 9 | 122946963 | del | TC | |  | FS | | 3104 | 1024 | F | | * |  |
| CEP70 | 3 | 139696542 | del | GTTTT | |  | FS | | 1991 | 597 | * | | ? |  |
| CEP76 | 18 | 12692534 | ref | GAGGCAGCGACATGCTGGCAGCCGGCGTCT | | GAGGCAGCGACATGCTGGCAGCCGGCGTCT | MS | | 156 | 0 | MSLPPEKASE | | RRRLPEKASE |  |
| CES1 | 16 | 54424465 | ref | CATCGTGG | | CATCGTGG | MS | | 108 | 0 | MWL | | PWL |  |
| CLEC4C | 12 | 7774698 | snp | C | | T | NS | | 948 | 152 | W | | * |  |
| CLEC7A | 12 | 10162353 | snp | A | | C | NS | | 762 | 191 | Y | | * |  |
| CLECL1 | 12 | 9776979 | ins |  | | TAAGT | FS | | 148 | 49 | Y | | YL |  |
| CLIC5 | 6 | 46091237 | ref | TGCCGTTGGC | | TGCCGTTGGC | MS | | 315 | 0 | MTDS | | VTDS |  |
| CLTCL1 | 22 | 17569004 | ins |  | | C | D | | 3675 | 1200 |  | | CCCTATTATCCGTAGG |  |
| CNOT1 | 16 | 57134828 | del | A | |  | FS | | 4909 | 1538 | C | | C |  |
| CNTN3 | 3 | 74652951 | ref | ATCTTTAATT | | ATCTTTAATT | MS | | 80 | 0 | MMFP | | KMFP |  |
| CNTNAP4 | 16 | 74869092 | ref | CGCTACTTCTGT | | CGCTACTTCTGT | FS | | 416 | 10 | TLLLL | | TLLLY |  |
| COG3 | 13 | 45001936 | ref | TGACCTTGCGG | | TGACCTTGCGG | NS | | 2341 | 746 | SDLAA | | N* |  |
| COL18A1 | 21 | 45748861 | del | A | |  | FS | | 2880 | 943 | P | | P |  |
| COL20A1 | 20 | 61427934 | ins |  | | C | FS | | 3544 | 1148 | P | | P |  |
| COL8A2 | 1 | 36335751 | ref | GCGGGTTATGT | | GCGGGTTATGT | N | | 2113 | 702 | T* | | TR |  |
| COMMD6 | 13 | 75009941 | ref | TGG | | TGG | MS | | 50 | 0 | M | | L |  |
| COPG2 | 7 | 129938905 | ref | CAGCAGCAGACTAGCTC | | CAGCAGCAGACTAGCTC | NS | | 1736 | 548 | NGLTVSV | | KS* |  |
| COX16 | 14 | 69862801 | ref | GTCAA | | GTCAA | N | | 407 | 105 | T* | | TD |  |
| CPSF1 | 8 | 145589339 | ref | GCT | | GCT | N | | 4405 | 1443 | * | | C |  |
| CRP | 1 | 157949934 | ref | GGCCT | | GGCCT | N | | 778 | 224 | * | | C |  |
| CSNK2A1 | 20 | 412603 | ref | GTTACTG | | GTTACTG | N | | 1446 | 390 | Q* | | SN |  |
| CST1 | 20 | 23679494 | ref | CTGGGCCATGGTCTCC | | CTGGGCCATGGTCTCC | MS | | 71 | 0 | MAQYLS | | GDHYLS |  |
| CTNNBIP1 | 1 | 9833345 | ref | TTCCAAGGGCTTTGCAGCTACTG | | TTCCAAGGGCTTTGCAGCTACTG | N | | 513 | 80 | Q* | | LE |  |
| CUL2 | 10 | 35400249 | ref | ATT | | ATT | MS | | 210 | 0 | M | | K |  |
| CYFIP2 | 5 | 156654444 | ins |  | | C | FS | | 421 | 94 | Q | | P |  |
| DCDC1 | 11 | 31283891 | - | CTCCAGCAGC | | TTC? | D | | 792 | 196 |  | | CTTGCTTTCTTCTA?GAA |  |
| DDIT4L | 4 | 101327898 | sub | TT | | C | FS | | 741 | 179 | K | | S |  |
| DDN | 12 | 47679141 | ref | ACCCGCGAGCCCAGTGGCTGGCCTGGAAGTCCATGGGCTGTCGAGA | | ACCCGCGAGCCCAGTGGCTGGCCTGGAAGTCCATGGGCTGTCGAGA | MS | | 180 | 0 | MDFQASHWARGFQNRT | | SRQPMDFQASHFQNRT |  |
| DDX60L | 4 | 169561697 | - | AGTTACTTACAAAAAAAATATTGCA | | NATTA? | D | | 3558 | 1112 |  | | ?TAATN |  |
| DGKK | X | 50137887 | ins |  | | G | FS | | 3119 | 1019 | G | | G |  |
| DHDH | 19 | 54134667 | ins |  | | G | FS | | 556 | 172 | A | | G |  |
| DHX8 | 17 | 38939926 | ins |  | | C | FS | | 1831 | 586 | I | | H |  |
| DIXDC1 | 11 | 111358318 | ins |  | | C | FS | | 1112 | 271 | P | | P |  |
| DOK4 | 16 | 56064772 | ref | GCTCGCCAGCACTGTGGT | | GCTCGCCAGCACTGTGGT | N | | 1272 | 326 | * | | C |  |
| DPF1 | 19 | 43394500 | ref | GTAGGCGAGCACCACCCCAGAGTCGCGGCGAGCCGAGCCGGCCTAGG | | GTAGGCGAGCACCACCCCAGAGTCGCGGCGAGCCGAGCCGGCCTAGG | N | | 1063 | 352 | T* | | TY |  |
| DUOX1 | 15 | 43233439 | - | GGTCTGAGCTCCCC | | ?TTCNNN | D | | 3678 | 1174 |  | | GTCCCTTCCACACA?TTCNNN |  |
| DUOX2 | 15 | 43192897 | ref | GCATGCCAACCCTGCAGCCTGCGGGGTG | | GCATGCCAACCCTGCAGCCTGCGGGGTG | MS | | 203 | 0 | MLRARPEALM | | AVRARPEALM |  |
| DYNC2H1 | 11 | 102496976 | snp | T | | C | D | | 1504 | 453 |  | | GCAAAA |  |
| ECSIT | 19 | 11477972 | ref | AGCCCGTGCCCTCGCGCCGGCTCAGACT | | AGCCCGTGCCCTCGCGCCGGCTCAGACT | N | | 1371 | 431 | * | | S |  |
| EDEM3 | 1 | 182943942 | del | AGCTG | |  | FS | | 1923 | 623 | PA | | P |  |
| EFEMP2 | 11 | 65396389 | ref | GCAGGGGAGCATCCTGGGG | | GCAGGGGAGCATCCTGGGG | MS | | 130 | 0 | MLPCASC | | PQDAASC |  |
| EGF | 4 | 111085749 | snp | G | | A | NS | | 1251 | 269 | W | | * |  |
| EGR3 | 8 | 22603925 | ref | GATCCT | | GATCCT | N | | 1521 | 387 | * | | C |  |
| EIF3A | 10 | 120785501 | ref | CCTATTTAAGACACCAGTTTAAATCCATTATCTTGAGACTTAACGTC | | CCTATTTAAGACACCAGTTTAAATCCATTATCTTGAGACTTAACGTC | N | | 4287 | 1380 | RR* | | LNR |  |
| EIF6 | 20 | 33335690 | ref | GCTCGGACCGCCATGAGGC | | GCTCGGACCGCCATGAGGC | MS | | 216 | 0 | MAVRASF | | ASWRSSF |  |
| ELA1 | 12 | 50009870 | ins |  | | G | FS | | 664 | 207 | G | | G |  |
| EMID2 | 7 | 100987391 | ins |  | | G | FS | | 1373 | 393 | G | | G |  |
| EML3 | 11 | 62131035 | del | AATCTGAAGGG | |  | FS | 1771 | | 487 | SPSDS | | S |  |
| EML5 | 14 | 88328597 | ref | CATGTCGGGG | | CATGTCGGGG | MS | | 186 | 0 | MAAR | | PAAR |  |
| ENTPD2 | 9 | 139064552 | sub | TCACC | | CCACGACAAAGTTCCCAGCCACCCGCCTGCCCTTACCCACG | D | | 1084 | 342 |  | | CGTGGGTAAGGGCAGGCGGGTGGCTGGGAACTTTGTCGTGGGT |  |
| EPHA2 | 1 | 16324269 | ref | CGATGGGGCTCCAGGCCCTGTCGAGGCT | | CGATGGGGCTCCAGGCCCTGTCGAGGCT | N | | 3067 | 976 | * | | W |  |
| EPHA7 | 6 | 94124692 | - | ACTTGTGCAC | | ?AT | D | | 1200 | 329 |  | | AT?GAGT |  |
| EPHA8 | 1 | 22800510 | del | GCGG | |  | FS | | 2985 | 953 | GG | | D |  |
| ERBB2IP | 5 | 65352937 | ref | TGGATTTGGGAAGTAACGAATTCACGG | | TGGATTTGGGAAGTAACGAATTCACGG | NS | | 873 | 188 | LDLGSNEFTE | | LGFGK* |  |
| ERCC8 | 5 | 60206176 | ref | AAAAAGGTACTAAAGATGATATTCATCCTTCTTCA | | AAAAAGGTACTAAAGATGATATTCATCCTTCTTCA | NS | | 1234 | 392 | DEEG* | | D* |  |
| ERGIC3 | 20 | 33599978 | del | CCGG | |  | FS | | 700 | 210 | AG | | E |  |
| ERN1 | 17 | 59475076 | ref | CGCT | | CGCT | N | | 3046 | 977 | * | | W |  |
| ESSPL | 4 | 152420476 | ins |  | | GTCAG | FS | | 131 | 43 | S | | RSA |  |
| ETFB | 19 | 56549431 | ins |  | | G | FS | | 477 | 91 | P | | P |  |
| ETS1 | 11 | 127837461 | ref | GCCATCACTCGTCGGCATCTGGCTTGAC | | GCCATCACTCGTCGGCATCTGGCTTGAC | N | | 1574 | 434 | VKPDADE* | | SQMPTSDG |  |
| ETV1 | 7 | 13917379 | ref | ACAACTTAACTTCTGTTCT | | ACAACTTAACTTCTGTTCT | FS | | 948 | 287 | RTEGCMF | | RTEAVCL |  |
| EXDL1 | 15 | 39263413 | ref | CCCATCTCTAGGGCAGATTTAGAAAA | | CCCATCTCTAGGGCAGATTTAGAAAA | N | | 1717 | 508 | PFLNLP* | | PICPRDG |  |
| FAM119A | 2 | 208197336 | ref | AGGGCCATTCC | | AGGGCCATTCC | MS | | 171 | 0 | MALV | | GMAV |  |
| FAM124B | 2 | 224974372 | del | C | |  | FS | | 582 | 118 | L | | L |  |
| FAM126B | 2 | 201596974 | ref | CATTT | | CATTT | MS | | 188 | 0 | ML | | KL |  |
| FAM38A | 16 | 87327962 | - | CCTGGGAGAGGGTCCG | | ?CA | D | | 2299 | 766 |  | | TG? |  |
| FAM83C | 20 | 33343506 | ref | CGGGCCTCCGAACATGCCTGCCA | | CGGGCCTCCGAACATGCCTGCCA | MS | | 118 | 0 | MFGGPGPG | | WQACSGPG |  |
| FASN | 17 | 77630306 | ref | CCTAGCCC | | CCTAGCCC | N | | 7646 | 2509 | EG* | | EAR |  |
| FCGR2C | 1 | 159832004 | snp | A | | G | D | | 888 | 266 |  | | GTGAGT |  |
| FHOD1 | 16 | 65838808 | ref | CGCCATGGCTCTGCGGCCGGCTCACGCAGCGCGC | | CGCCATGGCTCTGCGGCCGGCTCACGCAGCGCGC | MS | | 112 | 0 | MAGGEDRGDGEP | | ARGGEDRGDGEP |  |
| FLG2 | 1 | 150589755 | snp | G | | T | NS | | 7202 | 2376 | S | | * |  |
| FLJ10213 | 3 | 73194172 | ins |  | | A | FS | | 673 | 83 | R | | K |  |
| FLJ11506 | 15 | 65334019 | ref | CCATAGCTGCGCTCGCGAG | | CCATAGCTGCGCTCGCGAG | MS | | 105 | 0 | MAAGVPC | | LAAGVPC |  |
| FLJ20489 | 12 | 46460634 | ins |  | | T | FS | | 841 | 119 | L | | F |  |
| FLJ30428 | 2 | 131782940 | sub | CC | | G | FS | | 584 | 39 | FQ | | L |  |
| FLJ33360 | 5 | 6390305 | ref | CATG | | CATG | MS | | 97 | 0 | MD | | HD |  |
| FLJ33534 | 2 | 11181894 | snp | A | | G | D | | 1014 | 0 |  | | GCGAAC |  |
| FLJ37396 | 6 | 109655545 | snp | G | | T | D | | 1797 | 599 |  | | TTGAGG |  |
| FLJ39080 | 8 | 75827249 | del | A | |  | FS | | 305 | 82 | H | | P |  |
| FLJ41047 | 11 | 125212970 | snp | A | | C | D | | 96 | 16 |  | | CTCTCCACCCTGCCG |  |
| FLJ43093 | 6 | 36797126 | snp | C | | A | NS | | 1264 | 421 | S | | * |  |
| FLJ43826 | 17 | 34437251 | snp | A | | G | D | | 27 | 0 |  | | TTCCTGCTCCCCCGG |  |
| FLJ45139 | 21 | 39172632 | del | A | |  | FS | | 2273 | 25 | S | | S |  |
| FLJ45422 | 6 | 30337013 | ins |  | | T | FS | | 524 | 36 | L | | L |  |
| FLJ46481 | 4 | 6026285 | snp | C | | T | D | | 497 | 135 |  | | ATAGAG |  |
| FLOT2 | 17 | 24248696 | ref | CCACCGTGTGGCAATTGCCCATGGCGCCGGCGGCAC | | CCACCGTGTGGCAATTGCCCATGGCGCCGGCGGCAC | MS | | 123 | 0 | MGNCHTVGPNEA | | VPPAPWARPNEA |  |
| FN1 | 2 | 216008742 | ref | GCAGCCCGGGCCCCGGACCCCTAAGCATGT | | GCAGCCCGGGCCCCGGACCCCTAAGCATGT | MS | | 266 | 0 | MLRGPGPGLL | | TCLGVRGPGL |  |
| FOXN4 | 12 | 108208965 | del | GCAGG | |  | FS | | 270 | 6 | PA | | P |  |
| FOXS1 | 20 | 29897002 | ref | GCATGCTGGCCGGGCTGCCA | | GCATGCTGGCCGGGCTGCCA | MS | | 75 | 0 | MQQQPLP | | WQQQPLP |  |
| FSCB | 14 | 44044843 | ref | TCAGCAGGAGGCTCTTCTGAA | | TCAGCAGGAGGCTCTTCTGAA | NS | | 1344 | 358 | LSEEPPAE | | PFRRASC* |  |
| FSIP1 | 15 | 37697721 | - | CATGATGTGGACTCTAACTGATG | | ?GTGA? | D | | 1405 | 395 |  | | GGTTTGTTT?TCAC? |  |
| FUBP1 | 1 | 78187037 | ref | ATTATT | | ATTATT | N | | 2019 | 643 | Q* | | HN |  |
| FUCA2 | 6 | 143874453 | ref | TGGGGCCGCATGTCC | | TGGGGCCGCATGTCC | MS | | 92 | 0 | MRPQE | | GHAAE |  |
| FUT2 | 19 | 53898485 | snp | G | | A | NS | | 571 | 153 | W | | * |  |
| FZD8 | 10 | 35968273 | ref | TCCGCTCAGACCTGG | | TCCGCTCAGACCTGG | N | | 2080 | 691 | SQV* | | SLSG |  |
| GAK | 4 | 833457 | ref | GCCT | | GCCT | N | | 4134 | 1311 | * | | C |  |
| GATM | 15 | 43441594 | ref | CTGTT | | CTGTT | N | | 1600 | 423 | * | | W |  |
| GBA3 | 4 | 22429601 | ref | A | | A | NS | | 1416 | 455 | Y | | * |  |
| GDNF | 5 | 37870653 | ref | ATCTTAAAGTCCCGTCCGGCGGCGGCACCTGCGCGGGCAGGCG | | ATCTTAAAGTCCCGTCCGGCGGCGGCACCTGCGCGGGCAGGCG | MS | | 123 | 0 | MKLWDVVAVCLVLLH | | VKLWDVVAVCLVLLH |  |
| GEN1 | 2 | 17817508 | ref | TGA | | TGA | NS | | 1002 | 309 | NE | | S* |  |
| GGCX | 2 | 85642054 | ref | ACCGCCATTGC | | ACCGCCATTGC | MS | | 28 | 0 | MAVS | | AMAS |  |
| GIT2 | 12 | 108918399 | ref | GCCGTTTCGACATGGCT | | GCCGTTTCGACATGGCT | MS | | 71 | 0 | MSKRLR | | SHVEIR |  |
| GJA3 | 13 | 19614112 | ref | CCGGGCACTAGAT | | CCGGGCACTAGAT | N | | 1474 | 434 | I* | | AR |  |
| GLRA3 | 4 | 175801490 | ref | CATTTGCATGCCCCCAGAGACTTAAT | | CATTTGCATGCCCCCAGAGACTTAAT | N | | 1893 | 463 | D* | | EM |  |
| GOT1L1 | 8 | 37913463 | ins |  | | T | FS | | 794 | 231 | Q | | Q |  |
| GPATCH4 | 1 | 154831674 | ins |  | | CA | FS | | 1121 | 360 | T | | TV |  |
| GPBAR1 | 2 | 218835898 | ins |  | | C | FS | | 454 | 69 | T | | H |  |
| GPHB5 | 14 | 62854161 | ins |  | | G | FS | | 155 | 51 | G | | G |  |
| GPR158 | 10 | 25927467 | del | GCCTCAGAAA | | | FRAMESHIFT | | 2966 | 968 | KPQK | | N | |
| GPR44 | 11 | 60376577 | ref | CGGGTTCTAACTCGAGGTGCTGCTCAGCGCCCGGTTC | | CGGGTTCTAACTCGAGGTGCTGCTCAGCGCCCGGTTC | NS | | 1269 | 385 | LNRALSSTSS* | | LALSSTSS* |  |
| GPRIN1 | 5 | 175959426 | ref | TTCAGCAGTGTCCATCTGCCCTCA | | TTCAGCAGTGTCCATCTGCCCTCA | MS | | 175 | 0 | MDTAEDPA | | * |  |
| GREB1 | 2 | 11644957 | snp | G | | A | D | | 1475 | 386 |  | | TCTTGCCCACTGCAA |  |
| GRIP2 | 3 | 14536633 | ins |  | | G | FS | | 1308 | 436 | P | | P |  |
| GRM7 | 3 | 7163197 | del | CTTCTTCTCTCGCGTGGTGC | | | FRAMESHIFT | | 727 | 192 | DFFSRVVP | | D | |
| GTF2B | 1 | 89129753 | ref | CGGCTGGTAGACGCCATCTTCACGGCGACTGCGGT | | CGGCTGGTAGACGCCATCTTCACGGCGACTGCGGT | MS | | 119 | 0 | MASTSRLDALPR | | TAVAVNLDALPR |  |
| HDLBP | 2 | 241852585 | ref | CAG | | CAG | NS | | 409 | 60 | AG | | S* |  |
| HERC6 | 4 | 89519256 | ref | CAGCTGGGCCGCAGGG | | CAGCTGGGCCGCAGGG | FS | | 342 | 53 | QLGRRG | | QLGRRA |  |
| HIBCH | 2 | 190892719 | snp | A | | G | MS | | 84 | 0 | M | | T |  |
| HINT1 | 5 | 130528796 | ref | TCTCGGCCTCTCTCC | | TCTCGGCCTCTCTCC | MS | | 138 | 0 | MADEI | | VADEI |  |
| HLA-DPB2 | 6 | 33204500 | snp | T | | G | D | | 756 | 0 |  | | GGGAGA |  |
| HOXA3 | 7 | 27114052 | ref | CCACTATCA | | CCACTATCA | N | | 1045 | 285 | * | | W |  |
| HRASLS5 | 11 | 63015075 | ref | GGCCCATCCCGCCTCTGCGGC | | GGCCCATCCCGCCTCTGCGGC | MS | | 160 | 0 | MGLSPGA | | AAVSPGA |  |
| HUS1 | 7 | 47985632 | ref | AAACTTCATG | | AAACTTCATG | MS | | 130 | 0 | MKFR | | HEVR |  |
| HYDIN | 16 | 69453517 | del | A | |  | FS | | 11835 | 3902 | I | | I |  |
| ICAM3 | 19 | 10305529 | ref | GCGTCA | | GCGTCA | N | | 1656 | 547 | * | | R |  |
| IFNA10 | 9 | 21197036 | snp | A | | T | NS | | 105 | 19 | C | | * |  |
| IFNA17 | 9 | 21218003 | ins |  | | T | FS | | 218 | 56 | H | | Q |  |
| IL6ST | 5 | 55307861 | ref | ATCTTGCGCGGATATTT | | ATCTTGCGCGGATATTT | MS | | 255 | 0 | MLTLQT | | KLTLQT |  |
| ILDR1 | 3 | 123223610 | ref | CCATGCCGCCC | | CCATGCCGCCC | MS | | 106 | 0 | MAWP | | GPWP |  |
| INDOL1 | 8 | 39992091 | snp | T | | A | NS | | 1318 | 358 | Y | | * |  |
| IPO8 | 12 | 30739829 | ref | TGATCCGGTTGAGGTCCATCTCCCCGGGTGGGGGCT | | TGATCCGGTTGAGGTCCATCTCCCCGGGTGGGGGCT | MS | | 170 | 0 | MDLNRIIQALKG | | SPHPGRFQALKG |  |
| IREB2 | 15 | 76545732 | ref | TTAA | | TTAA | NS | | 637 | 158 | LK | | V* |  |
| ITIH5 | 10 | 7645083 | del | C | |  | FS | | 2215 | 718 | D | | T |  |
| ITPR2 | 12 | 26726073 | ins |  | | TCAC | FS | | 1826 | 469 | R | | R* |  |
| JRK | 8 | 143743089 | del | CA | |  | FS | | 1900 | 462 | W | | E |  |
| KARS | 16 | 74239025 | ref | GCACGGCCGCCATCTTC | | GCACGGCCGCCATCTTC | MS | | 40 | 0 | MAAVQA | | EDGGQA |  |
| KCNIP2 | 10 | 103593310 | ref | CGCATGG | | CGCATGG | MS | | 352 | 0 | MRG | | PWG |  |
| KCNQ2 | 20 | 61508434 | ref | GCCGCCTCACTTCCTGGGCCCGGC | | GCCGCCTCACTTCCTGGGCCCGGC | NS | | 2724 | 849 | AGPRK* | | PRK* |  |
| KCTD19 | 16 | 65918090 | ref | CCAGAGCGGGCTCCGTACCATGGTCGCGGCTC | | CCAGAGCGGGCTCCGTACCATGGTCGCGGCTC | MS | | 51 | 0 | MEESGMAHESA | | EEESGMAHESA |  |
| KIAA0090 | 1 | 19450580 | ref | CAGCCGCCATGATGCGAGCGCATGCACCACCC | | CAGCCGCCATGATGCGAGCGCATGCACCACCC | MS | | 43 | 0 | MAAEWASRFWL | | GWCKWASRFWL |  |
| KIAA0256 | 15 | 47071700 | ref | CAGCTGGAGATAGAGAGCCGACATTTCCTGAGTTACGTAGTTTGCG | | CAGCTGGAGATAGAGAGCCGACATTTCCTGAGTTACGTAGTTTGCG | N | | 3291 | 1052 | TQTT* | | IYLQL |  |
| KIAA0644 | 7 | 28964168 | ref | AGGCGCGGGCAGCCTCCATCGC | | AGGCGCGGGCAGCCTCCATCGC | MS | | 367 | 0 | MEAARALR | | AMEAARVR |  |
| KIAA1161 | 9 | 34362872 | ref | C | | C | NS | | 235 | 22 | Y | | * |  |
| KIAA1751 | 1 | 1876878 | snp | A | | G | N | | 2442 | 762 | * | | Q |  |
| KIAA1908 | 7 | 1594157 | snp | T | | C | N | | 1479 | 264 | * | | R |  |
| KIF20B | 10 | 91458902 | - | GGC | | NGG | D | | 219 | 49 |  | | CCTTTATTTTTTAANGG |  |
| KLK14 | 19 | 56277789 | snp | C | | T | D | | 244 | 8 |  | | ATAAGT |  |
| KRT24 | 17 | 36111660 | del | A | |  | FS | | 722 | 221 | N | | K |  |
| KRTAP10-3 | 21 | 44802356 | ref | CCCGT | | CCCGT | N | | 710 | 221 | * | | W |  |
| KRTAP19-6 | 21 | 30835852 | del | G | |  | FS | | 199 | 56 | F | | F |  |
| LCAP | X | 152802902 | ins |  | | C | FS | | 539 | 11 | W | | L |  |
| LCN10 | 9 | 138754315 | snp | G | | A | NS | | 536 | 160 | Q | | * |  |
| LCP1 | 13 | 45599723 | ref | GCCT | | GCCT | N | | 2056 | 627 | * | | C |  |
| LDB1 | 10 | 103857827 | ref | CAGCAGGGCCTTTTACTGGGAGG | | CAGCAGGGCCTTTTACTGGGAGG | N | | 1740 | 372 | ASQ* | | EALL |  |
| LEF1 | 4 | 109308362 | ref | GTTGGGGCATCCC | | GTTGGGGCATCCC | MS | | 654 | 0 | MPQLS | | GMPLS |  |
| LENG9 | 19 | 59666499 | ref | CCGGCTCTCTGGCCGCTGCCATGGGTGCGGGGAACTG | | CCGGCTCTCTGGCCGCTGCCATGGGTGCGGGGAACTG | MS | | 185 | 0 | MAAAREPELPQEA | | QFPAPMAELPQEA |  |
| LEPREL2 | 12 | 6808249 | ref | CGCAGCACGGAGGCTGGGCCCCGGGGGCGCGGCGCG | | CGCAGCACGGAGGCTGGGCCCCGGGGGCGCGGCGCG | FS | | 417 | 127 | CAARRLGPGGAAR | | CAARRLGPGGAAR |  |
| LGALS7 | 19 | 43955962 | ref | TCACGGACATGGCTGGGACCGGGTTGGGCAGCC | | TCACGGACATGGCTGGGACCGGGTTGGGCAGCC | MS | | 29 | 0 | MSNVPHKSSLP | | RSNVPHKSSLP |  |
| LGI1 | 10 | 95542643 | ref | GATTGCATCATTA | | GATTGCATCATTA | NS | | 881 | 219 | DCIIT | | GLHH* |  |
| LGI4 | 19 | 40309236 | del | TGCAGGCT | | | FRAMESHIFT | | 1587 | 356 | SLH | | R | |
| LIN7A | 12 | 79855623 | ref | CTTCAGCATCAGCAACCGCT | | CTTCAGCATCAGCAACCGCT | MS | | 193 | 0 | MLKPSVT | | SGCPSVT |  |
| LMBRD1 | 6 | 70563491 | ref | CATCTT | | CATCTT | MS | | 115 | 0 | MA | | KA |  |
| LOC100128076 | 9 | 93936046 | ins |  | | CCAC | FS | | 133 | 44 | Q | | PT |  |
| LOC100128197 | 5 | 180168187 | snp | G | | A | NS | | 231 | 77 | R | | * |  |
| LOC100128252 | 19 | 61698226 | snp | A | | G | D | | 183 | 61 |  | | TCCCTCCTAATCAGG |  |
| LOC100128300 | 10 | 99619097 | del | TG | |  | FS | | 117 | 39 | W | | V |  |
| LOC100128370 | 7 | 39955769 | ref | TACGTCA | | TACGTCA | N | | 711 | 237 | * | | V |  |
| LOC100128499 | 6 | 4024677 | snp | G | | A | NS | | 767 | 189 | R | | * |  |
| LOC100128610 | 6 | 63997561 | ins |  | | G | FS | | 729 | 138 | N | | Q |  |
| LOC100128718 | 19 | 17377627 | snp | G | | A | D | | 5 | 1 |  | | TTGGGCCCTTCCCAA |  |
| LOC100128781 | 14 | 87480972 | snp | G | | T | D | | 52 | 0 |  | | TTATGT |  |
| LOC100128785 | 16 | 1095025 | snp | C | | T | NS | | 985 | 328 | W | | * |  |
| LOC100128816 | 12 | 3591205 | snp | A | | T | NS | | 211 | 70 | L | | * |  |
| LOC100128842 | 1 | 1184957 | ref | CCATGTCC | | CCATGTCC | MS | | 516 | 0 | MGG | | GRG |  |
| LOC100128890 | 8 | 7170572 | snp | A | | G | D | | 20 | 6 |  | | GCAACA |  |
| LOC100128892 | 3 | 113849658 | ins |  | | C | FS | | 85 | 28 | R | | R |  |
| LOC100128908 | 14 | 22643964 | ref | AGCATC | | AGCATC | MS | | 42 | 0 | ML | | DA |  |
| LOC100128995 | 8 | 12289411 | snp | G | | A | D | | 38 | 12 |  | | ATAAGG |  |
| LOC100129026 | 5 | 172132094 | snp | G | | A | NS | | 1745 | 372 | W | | * |  |
| LOC100129058 | 3 | 51883265 | snp | T | | C | N | | 531 | 117 | * | | Q |  |
| LOC100129194 | 3 | 32254857 | snp | T | | C | N | | 1679 | 172 | * | | Q |  |
| LOC100129240 | 4 | 186145645 | del | TG | |  | FS | | 622 | 192 | H | | H |  |
| LOC100129328 | 7 | 27739959 | ins |  | | T | FS | | 1081 | 360 | E | | E |  |
| LOC100129502 | 15 | 88242600 | snp | T | | C | MS | | 692 | 0 | M | | V |  |
| LOC100129509 | 12 | 51058522 | del | AGGGAGTGGGG |  | | FS | | 1079 | 359 | GGSGV | | G | |
| LOC100129668 | 6 | 31488140 | del | GCTG | |  | FS | | 991 | 317 | GC | | A |  |
| LOC100129733 | 6 | 126122534 | ref | TCAGC | | TCAGC | MS | | 256 | 0 | MA | | VA |  |
| LOC100129786 | 4 | 3613965 | ins |  | | TG | FS | | 141 | 47 | H | | H |  |
| LOC100129932 | 11 | 93502140 | snp | A | | C | D | | 142 | 44 |  | | GGTGAG |  |
| LOC100129940 | 12 | 64562030 | ref | TC | | TC | MS | | 57 | 0 | M | | V |  |
| LOC100129969 | 9 | 34883045 | sub | CC | | G | FS | | 409 | 117 | LA | | L |  |
| LOC100130069 | 1 | 11954657 | sub | AGGTACTTTCTCCAAG | | GTCCCC | FS | | 465 | 122 | RYFLQD | | VP |  |
| LOC100130107 | 15 | 57847773 | snp | C | | T | NS | | 934 | 254 | W | | * |  |
| LOC100130108 | 19 | 22660735 | snp | A | | T | NS | | 942 | 314 | K | | * |  |
| LOC100130193 | 1 | 19600564 | del | CT | |  | FS | | 307 | 102 | K | | K |  |
| LOC100130226 | 20 | 61675566 | del | G | |  | FS | | 360 | 120 | P | | P |  |
| LOC100130255 | 10 | 123913642 | ref | GGGGCTGCTCTGGGAGGAGCATCT | | GGGGCTGCTCTGGGAGGAGCATCT | MS | | 218 | 0 | MLLPEQPQ | | RCSSQSSQ |  |
| LOC100130262 | 1 | 247108676 | ref | GTGTCT | | GTGTCT | N | | 1677 | 165 | * | | C |  |
| LOC100130297 | 18 | 54901992 | snp | T | | C | D | | 106 | 35 |  | | GCGCGT |  |
| LOC100130315 | 2 | 133592157 | del | T | |  | FS | | 231 | 77 | S | | H |  |
| LOC100130363 | 15 | 57768010 | ins |  | | CA | FS | | 46 | 15 | R | | T |  |
| LOC100130460 | 11 | 10388945 | snp | A | | C | N | | 567 | 86 | * | | C |  |
| LOC100130581 | 17 | 38821646 | del | CGGCGCATGGA | |  | FS | 441 | | 0 | | MRRL | P | |
| LOC100130658 | 17 | 59933756 | ref | CTCGGGCTAGC | | CTCGGGCTAGC | N | | 298 | 99 | G* | | AE |  |
| LOC100130771 | 7 | 105009144 | ref | ATC | | ATC | MS | | 27 | 0 | M | | E |  |
| LOC100130773 | 22 | 24101776 | ref | CACAGAAAGGCTGCTGCTCCTGGCTCAGCACC | | CACAGAAAGGCTGCTGCTCCTGGCTCAGCACC | N | | 375 | 120 | RC* | | SLS |  |
| LOC100130825 | 12 | 130714627 | snp | G | | A | NS | | 288 | 96 | R | | * |  |
| LOC100131015 | 2 | 230404833 | sub | G | | TT | FS | | 1012 | 53 | T | | K |  |
| LOC100131018 | 7 | 5696831 | del | G | |  | FS | | 603 | 73 | S | | L |  |
| LOC100131032 | 1 | 208697895 | snp | C | | T | NS | | 739 | 135 | Q | | * |  |
| LOC100131117 | 3 | 115568565 | snp | G | | A | D | | 728 | 242 |  | | ATAAGT |  |
| LOC100131409 | 2 | 145141870 | snp | C | | G | NS | | 17 | 5 | Y | | * |  |
| LOC100131601 | 16 | 73838172 | ref | CATG | | CATG | MS | | 316 | 0 | MA | | HA |  |
| LOC100131626 | 11 | 117757378 | ref | TGGAGACATCAGC | | TGGAGACATCAGC | N | | 311 | 56 | C* | | SP |  |
| LOC100131627 | 14 | 79147921 | snp | T | | G | N | | 143 | 40 | * | | Y |  |
| LOC100131637 | 8 | 86277502 | snp | G | | C | NS | | 167 | 55 | Y | | * |  |
| LOC100131646 | 1 | 101475135 | del | G | |  | FS | | 314 | 104 | Y | | Y |  |
| LOC100131672 | 9 | 111841707 | snp | C | | T | D | | 10 | 0 |  | | AGTTAGAACTAGGAA |  |
| LOC100131811 | 4 | 184601351 | snp | C | | T | NS | | 1063 | 283 | W | | * |  |
| LOC100131949 | 11 | 65865235 | snp | C | | T | NS | | 907 | 269 | Q | | * |  |
| LOC100132033 | 17 | 43480189 | snp | C | | T | NS | | 2688 | 77 | R | | * |  |
| LOC100132078 | 11 | 110792366 | snp | T | | C | MS | | 1758 | 0 | M | | V |  |
| LOC100132159 | 10 | 5548146 | ins |  | | C | FS | | 407 | 29 | C | | W |  |
| LOC100132252 | 6 | 33959029 | snp | C | | A | NS | | 427 | 142 | S | | * |  |
| LOC100132361 | 6 | 26435934 | snp | T | | C | N | | 1101 | 220 | * | | Q |  |
| LOC100132440 | 16 | 51346201 | ins |  | | CACA | FS | | 338 | 112 | S | | SV |  |
| LOC100132501 | 18 | 51599358 | ins |  | | T | FS | | 592 | 106 | R | | K |  |
| LOC100132547 | 12 | 49045698 | - | AAAATGAA | | NAAN | D | | 0 | 0 |  | | GCCGGAGTGGCCNAAN |  |
| LOC100132557 | X | 147946364 | ins |  | | G | FS | | 216 | 40 | K | | E |  |
| LOC100132605 | 5 | 721656 | del | GACA | |  | FS | | 573 | 191 | DN | | I |  |
| LOC100132634 | 11 | 20648612 | del | G | |  | FS | | 426 | 142 | P | | P |  |
| LOC100132713 | 18 | 74482671 | del | TCTATGGAAGTTTC | | | FRAMESHIFT | | 2958 | 986 | SMEVS | | S | |
| LOC100132730 | 10 | 50131747 | ins |  | | ATGTGTGT | FS | | 16 | 5 | C | | YVC |  |
| LOC100132821 | 2 | 240503106 | del | CA | |  | D | | 763 | 254 |  | | TGTGT |  |
| LOC100132891 | 8 | 72918202 | ins |  | | T | FS | | 12 | 4 | G | | W |  |
| LOC100132893 | 18 | 75616929 | ins |  | | C | FS | | 682 | 55 | R | | R |  |
| LOC100132913 | 1 | 120637860 | ref | AAAGATGCTGCAAATCAAATCCCAAACGGG | | AAAGATGCTGCAAATCAAATCCCAAACGGG | N | | 668 | 222 | LPFGI* | | LICSIF |  |
| LOC100132945 | 4 | 3964409 | ins |  | | ACACACATGC | FS | | 60 | 20 | A | | ACVC |  |
| LOC100132980 | 15 | 18824191 | ins |  | | GGGA | FS | | 263 | 73 | I | | RD |  |
| LOC100133003 | 2 | 87136403 | snp | C | | G | N | | 1117 | 372 | * | | S |  |
| LOC100133181 | 3 | 157875661 | ins |  | | G | FS | | 2008 | 21 | P | | P |  |
| LOC100133199 | 4 | 1518978 | ins |  | | T | FS | | 1017 | 339 | A | | C |  |
| LOC100133208 | 16 | 66023674 | del | G | |  | FS | | 785 | 261 | S | | S |  |
| LOC100133260 | 7 | 73905901 | ref | TGCATTTGCTTGGGGG | | TGCATTTGCTTGGGGG | MS | | 104 | 0 | MHSAAK | | PPSAAK |  |
| LOC100133289 | 21 | 46312972 | del | TG | |  | FS | | 433 | 144 | M | | M |  |
| LOC100133296 | 10 | 3160698 | ins |  | | AC | FS | | 1241 | 217 | E | | V |  |
| LOC120824 | 11 | 48956998 | snp | C | | G | D | | 737 | 245 |  | | CTACGG |  |
| LOC123855 | 16 | 83776222 | - | CA | | ?G | D | | 2283 | 761 |  | | TGTTTTGTCTTT?GG |  |
| LOC126520 | 19 | 1485041 | snp | T | | C | D | | 1535 | 0 |  | | GCGAGC |  |
| LOC128322 | 1 | 113092240 | snp | T | | C | MS | | 1 | 0 | M | | T |  |
| LOC134466 | 5 | 150291044 | snp | C | | T | D | | 1077 | 0 |  | | TTATACATTAAAGAA |  |
| LOC145814 | 15 | 97329327 | ins |  | | C | FS | | 699 | 164 | V | | G |  |
| LOC147645 | 19 | 56527705 | ins |  | | G | FS | | 2575 | 858 | A | | A |  |
| LOC150297 | 22 | 30875524 | ref | CACTAAAGCCGGA | | CACTAAAGCCGGA | N | | 786 | 248 | LRL* | | PALV |  |
| LOC158257 | 9 | 96061444 | del | CG | |  | FS | | 490 | 163 | T | | T |  |
| LOC202051 | 5 | 138767633 | ref | GAGGGGCGTCGCCATCTTCCGCCCCCGC | | GAGGGGCGTCGCCATCTTCCGCCCCCGC | MS | | 4 | 0 | MATPLGWSKA | | EDGDAGWSKA |  |
| LOC283710 | 15 | 29308798 | del | GG | |  | FS | | 187 | 24 | P | | P |  |
| LOC284288 | 18 | 53657053 | del | G | |  | FS | | 348 | 116 | E | | S |  |
| LOC285346 | 3 | 44515799 | del | TC | |  | FS | | 1620 | 491 | G | | G |  |
| LOC285908 | 7 | 65479978 | sub | GT | | ATGC | FS | | 2909 | 17 | T | | AC |  |
| LOC339742 | 2 | 132823314 | ins |  | | C | FS | | 10 | 3 | I | | T |  |
| LOC339766 | 2 | 234369425 | - | CCTTGGTGAGGCTCTGCGGCA | | NCTCG?GAGG? | D | | 1313 | 412 |  | | NCTCG?GAGG? |  |
| LOC340947 | 10 | 38409778 | del | CT | |  | FS | | 160 | 53 | K | | K |  |
| LOC341346 | 12 | 27514702 | ins |  | | T | FS | | 14 | 4 | L | | F |  |
| LOC341689 | 13 | 58002084 | snp | G | | C | D | | 1663 | 0 |  | | CTATTC |  |
| LOC344382 | 2 | 37897999 | del | A | |  | FS | | 438 | 146 | R | | G |  |
| LOC344595 | 3 | 108446710 | snp | T | | C | N | | 1029 | 343 | * | | R |  |
| LOC348751 | 2 | 200424321 | - | CCACAATTCTCTAGTCAATTAATGACTTTAAAGGGGCATTGGCATCT | | ?ATTGGA? | D | | 68 | 22 |  | | ?TCCAAT? |  |
| LOC375295 | 2 | 177206062 | snp | A | | C | N | | 1506 | 502 | * | | E |  |
| LOC388780 | 20 | 2135942 | sub | GC | | TT | NS | | 152 | 50 | PQ | | P* |  |
| LOC390084 | 11 | 5946137 | sub | CC | | G | FS | | 161 | 53 | LE | | L |  |
| LOC390282 | 12 | 5012183 | del | CT | |  | FS | | 171 | 57 | R | | S |  |
| LOC391282 | 21 | 39420970 | snp | T | | C | D | | 8 | 2 |  | | GCGAGT |  |
| LOC399829 | 10 | 134638300 | snp | C | | T | NS | | 216 | 72 | R | | * |  |
| LOC400950 | 2 | 42019257 | snp | C | | T | D | | 468 | 125 |  | | TCCTCTTTCTTGCAA |  |
| LOC401074 | 3 | 75872431 | snp | A | | G | D | | 960 | 320 |  | | TTTTTTTTTTTGTGG |  |
| LOC401847 | 16 | 33477695 | snp | G | | C | D | | 332 | 110 |  | | CTGAGT |  |
| LOC440287 | 15 | 72179598 | del | A | |  | FS | | 600 | 200 | Y | | M |  |
| LOC440419 | 17 | 22769813 | snp | T | | A | D | | 422 | 0 |  | | GAGAGC |  |
| LOC440981 | 3 | 147596268 | del | T | |  | FS | | 546 | 182 | K | | K |  |
| LOC441054 | 4 | 186598868 | - | TA | | NG | D | | 943 | 238 |  | | GNGAGT |  |
| LOC554235 | 19 | 55706793 | ref | GCTTCTCAGCAGAGATGGATCCC | | GCTTCTCAGCAGAGATGGATCCC | N | | 700 | 173 | GIHLC* | | PSLLRS |  |
| LOC642502 | 17 | 1708195 | snp | G | | A | NS | | 303 | 98 | R | | * |  |
| LOC643100 | 4 | 76325113 | snp | T | | C | D | | 170 | 56 |  | | TCCTTGGGGACCGGG |  |
| LOC643339 | 12 | 92245931 | snp | A | | G | D | | 322 | 92 |  | | GCATGT |  |
| LOC644366 | 17 | 42263261 | snp | C | | T | NS | | 571 | 190 | W | | * |  |
| LOC644578 | 4 | 63898085 | snp | T | | C | D | | 249 | 83 |  | | GCGAGG |  |
| LOC644974 | 3 | 131297625 | del | C | |  | FS | | 507 | 169 | L | | W |  |
| LOC645018 | 4 | 144490515 | del | AGAT | |  | FS | | 780 | 67 | IY | | I |  |
| LOC645277 | 12 | 131691471 | snp | T | | C | D | | 244 | 81 |  | | CGGGGAGCCATGCGG |  |
| LOC645984 | 10 | 31259453 | ref | AGCGTGGCCAGAGTGGACGCCATGG | | AGCGTGGCCAGAGTGGACGCCATGG | MS | | 255 | 0 | MASTLATLE | | PWRPLWPRE |  |
| LOC646268 | 1 | 156368733 | snp | T | | G | N | | 372 | 75 | * | | S |  |
| LOC646357 | 13 | 23811018 | snp | C | | A | NS | | 452 | 53 | E | | * |  |
| LOC646360 | 10 | 37638251 | snp | A | | T | NS | | 133 | 13 | K | | * |  |
| LOC646450 | 3 | 48931585 | ref | TTACCCGCC | | TTACCCGCC | MS | | 236 | 0 | MLG | | VLG |  |
| LOC646609 | 9 | 22724372 | snp | T | | C | D | | 53 | 0 |  | | GCAGAT |  |
| LOC646753 | 9 | 30822123 | del | T | |  | FS | | 127 | 33 | K | | R |  |
| LOC646879 | 13 | 36266716 | del | GATG | |  | FS | | 96 | 32 | DE | | K |  |
| LOC646934 | 15 | 76817632 | snp | A | | C | D | | 1521 | 0 |  | | CTCCTGTCTGCACCG |  |
| LOC647055 | 18 | 44295289 | - | CACCTGCA | | NNNCCGNN | D | | 1715 | 571 |  | | CTGGAANNNCCGNNG |  |
| LOC647087 | 7 | 134997787 | ins |  | | C | FS | | 262 | 87 | R | | P |  |
| LOC647597 | 2 | 91300189 | snp | G | | A | NS | | 317 | 79 | R | | * |  |
| LOC648665 | 8 | 6928011 | snp | G | | C | D | | 109 | 0 |  | | CTAAAG |  |
| LOC652968 | 22 | 29015484 | ref | ATCGCGGCTCGC | | ATCGCGGCTCGC | MS | | 16 | 0 | MELH | | AELH |  |
| LOC653125 | 15 | 30472262 | ref | TGATGGTGATGTTTATCTC | | TGATGGTGATGTTTATCTC | N | | 1799 | 599 | RR* | | RPS |  |
| LOC727827 | 6 | 469893 | ins |  | | GAGT | FS | | 154 | 26 | V | | VS |  |
| LOC728117 | 21 | 45877483 | snp | G | | A | NS | | 1317 | 413 | R | | * |  |
| LOC728135 | 3 | 75512060 | del | CA | |  | FS | | 432 | 144 | C | | C |  |
| LOC728148 | 12 | 46094948 | snp | G | | A | D | | 393 | 131 |  | | CATCTGTGACAAAAA |  |
| LOC728203 | 4 | 186147200 | sub | TG | | CA | MS | | 569 | 0 | M | | T |  |
| LOC728224 | 17 | 36507772 | snp | A | | T | NS | | 89 | 29 | C | | * |  |
| LOC728275 | 6 | 164267962 | snp | C | | T | NS | | 321 | 107 | Q | | * |  |
| LOC728318 | 17 | 36599664 | snp | A | | G | MS | | 0 | 0 | M | | V |  |
| LOC728362 | 6 | 88088850 | ref | TTCAGCCCGCAGACCCCA | | TTCAGCCCGCAGACCCCA | N | | 525 | 131 | LGSAG* | | WGLRAE |  |
| LOC728450 | 20 | 11738887 | ins |  | | TT | FS | | 271 | 70 | E | | E |  |
| LOC728530 | 4 | 88075977 | ref | ACTTA | | ACTTA | N | | 657 | 219 | * | | S |  |
| LOC728591 | 13 | 35699335 | ref | TTTTAGCTCT | | TTTTAGCTCT | N | | 810 | 239 | KS* | | KLK |  |
| LOC728647 | 10 | 59943202 | del | CCGG | |  | FS | | 1367 | 89 | NR | | N |  |
| LOC728661 | 1 | 1587062 | ref | GCAGCTGGCAGCTTCCTGCTCTCAGGGATGCTG | | GCAGCTGGCAGCTTCCTGCTCTCAGGGATGCTG | N | | 1677 | 559 | QHP* | | AASC |  |
| LOC728677 | 17 | 78127497 | ref | TGGGC | | TGGGC | MS | | 829 | 0 | MS | | VS |  |
| LOC728780 | 5 | 75000877 | snp | G | | A | NS | | 1876 | 625 | W | | * |  |
| LOC728804 | 16 | 29058253 | ref | AGCCGCAGCAGCATCCAGGGTCT | | AGCCGCAGCAGCATCCAGGGTCT | MS | | 244 | 0 | MLLRLCKV | | RPWMLCKV |  |
| LOC729054 | 10 | 53738611 | sub | AAT | | GG | D | | 0 | 0 |  | | AGCGCGTGAACAATGG |  |
| LOC729111 | 19 | 46698494 | del | A | |  | FS | | 255 | 83 | C | | A |  |
| LOC729112 | 4 | 122590615 | snp | T | | A | NS | | 266 | 8 | Y | | * |  |
| LOC729479 | 13 | 79498382 | ins |  | | A | FS | | 146 | 16 | Q | | T |  |
| LOC729534 | 3 | 135802412 | snp | T | | A | N | | 639 | 97 | * | | K |  |
| LOC729568 | 22 | 20059867 | snp | A | | G | N | | 907 | 302 | * | | W |  |
| LOC729595 | 5 | 179054405 | ins |  | | A | FS | | 602 | 200 | K | | K |  |
| LOC729605 | 11 | 31794829 | ins |  | | G | FS | | 1536 | 512 | R | | P |  |
| LOC729616 | 6 | 153346280 | snp | C | | T | NS | | 327 | 109 | R | | * |  |
| LOC729637 | 14 | 61081591 | ins |  | | C | FS | | 167 | 5 | R | | R |  |
| LOC729659 | 7 | 102689898 | snp | C | | T | NS | | 216 | 72 | Q | | * |  |
| LOC729722 | 15 | 18344202 | ins |  | | A | FS | | 243 | 81 | Y | | I |  |
| LOC729789 | 3 | 54635725 | snp | T | | A | N | | 465 | 148 | * | | K |  |
| LOC729847 | 16 | 78809591 | ref | GATGTT | | GATGTT | N | | 265 | 88 | * | | F |  |
| LOC729878 | 16 | 3440040 | sub | GAAAAG | | A | FS | | 240 | 67 | EK | | K |  |
| LOC729978 | 16 | 11934975 | del | AT | |  | FS | | 1757 | 111 | H | | R |  |
| LOC729987 | 1 | 98448954 | snp | G | | A | D | | 100 | 0 |  | | ATAAAA |  |
| LOC730013 | 16 | 14826014 | ref | CAGGCGCGGCCATCGGCG | | CAGGCGCGGCCATCGGCG | MS | | 54 | 0 | MAAPAE | | RRWPPE |  |
| LOC730018 | 16 | 85893848 | ref | GGGACACGGACATGGGGACAAGTCCCCCCTTA | | GGGACACGGACATGGGGACAAGTCCCCCCTTA | MS | | 2 | 0 | MSVSLGEGSYF | | PCPCLGEGSYF |  |
| LOC730085 | 2 | 132778692 | sub | C | | TG | D | | 106 | 35 |  | | CATGCAT |  |
| LOC730141 | 6 | 52174605 | snp | G | | A | NS | | 61 | 2 | W | | * |  |
| LOC730236 | 13 | 66702739 | ins |  | | G | FS | | 24 | 8 | R | | E |  |
| LOC730382 | 7 | 56720601 | ins |  | | T | FS | | 760 | 253 | E | | E |  |
| LOC730512 | 12 | 39186403 | snp | G | | A | D | | 642 | 214 |  | | ATAATT |  |
| LOC731511 | 11 | 67269552 | del | ACACACACAT | | | FRAMESHIFT | | 177 | 59 | MCVY | | T | |
| LOC731852 | 19 | 6905724 | snp | G | | A | NS | | 318 | 90 | W | | * |  |
| LOC731954 | 10 | 31040315 | sub | C | | TA | FS | | 242 | 80 | H | | HT |  |
| LOC732174 | 6 | 170615937 | snp | G | | A | D | | 278 | 52 |  | | ATGAGT |  |
| LOC81691 | 16 | 20743339 | del | A | |  | FS | | 891 | 231 | D | | A |  |
| LOC92973 | 9 | 35850654 | del | CATT | |  | FS | | 384 | 26 | FI | | L |  |
| LOC96597 | 17 | 16769883 | snp | C | | T | D | | 951 | 0 |  | | ATAAGT |  |
| LONP1 | 19 | 5643032 | ref | GGGGTGGCCGTCACCGTTCCACGGCCAGCGC | | GGGGTGGCCGTCACCGTTCCACGGCCAGCGC | N | | 2892 | 953 | ALAVER* | | WNGDGHP |  |
| LRP6 | 12 | 12165321 | ref | CCTCCTC | | CCTCCTC | N | | 4982 | 1613 | * | | W |  |
| LRRC19 | 9 | 26988216 | del | T | |  | FS | | 193 | 34 | E | | E |  |
| LSM4 | 19 | 18294806 | ref | CCGCAGTTGCCCTGCCCTCACCATGGTGCCGGCGGGG | | CCGCAGTTGCCCTGCCCTCACCATGGTGCCGGCGGGG | MS | | 134 | 0 | MLPLSLLKTAQNH | | PLPLSLLKTAQNH |  |
| MAF | 16 | 78185841 | ref | AGCTCTCACACAAATTTCATTTT | | AGCTCTCACACAAATTTCATTTT | N | | 2014 | 401 | TK* | | TRA |  |
| MAFG | 17 | 77474348 | ref | GTCATAACCCGGGGGCACAGCGAGCAGGCGCTCTCTGCAA | | GTCATAACCCGGGGGCACAGCGAGCAGGCGCTCTCTGCAA | MS | | 209 | 0 | MTTPNKGNKALKVK | | RATPNKGNKALKVK |  |
| MAGEB16 | X | 35731047 | snp | C | | T | NS | | 1092 | 271 | R | | * |  |
| MAGI2 | 7 | 77486560 | ref | GCGCGGCTCATCTGCTGGCGGCCGAGGCGCCGGG | | GCGCGGCTCATCTGCTGGCGGCCGAGGCGCCGGG | N | | 4595 | 1447 | PGASAASR* | | PRPPADEPR |  |
| MAML2 | 11 | 95714690 | ref | GGCGCTGTGTCCCCCATCTTA | | GGCGCTGTGTCCCCCATCTTA | MS | | 1285 | 0 | MGDTAPP | | * |  |
| MAP2K3 | 17 | 21144802 | snp | C | | T | NS | | 568 | 101 | Q | | * |  |
| MAPK15 | 8 | 144873623 | - | ACCGCCATCTGAGGAGGGTGAGCCAGGCTGCTGGGGCTGGGCACCG | | ?A | D | | 762 | 240 |  | | ?A |  |
| MBTPS1 | 16 | 82645549 | ref | GCCGGTCACACC | | GCCGGTCACACC | N | | 3654 | 1050 | SV* | | STG |  |
| MCM6 | 2 | 136342724 | snp | G | | A | NS | | 616 | 180 | R | | * |  |
| MED13L | 12 | 115199313 | ref | AGTCATGATCCTCCGCGAGCCCGGC | | AGTCATGATCCTCCGCGAGCCCGGC | MS | | 207 | 0 | MTAAANWVA | | AGAAANWVA |  |
| MEIS2 | 15 | 35177458 | ref | CCCATAGATCGCGTCCTTGTCCCGCTTCAAGGCGTCGT | | CCCATAGATCGCGTCCTTGTCCCGCTTCAAGGCGTCGT | MS | | 725 | 0 | MALVFEKCELATC | | TTLVFEKCELATC |  |
| MEX3D | 19 | 1506327 | ref | ACTCAATACTG | | ACTCAATACTG | N | | 1992 | 664 | QY* | | VLS |  |
| MFAP1 | 15 | 41884577 | ref | TGGACCCTAGGTAGT | | TGGACCCTAGGTAGT | NS | | 1495 | 437 | TT* | | * |  |
| MGC16703 | 22 | 19693743 | snp | T | | C | D | | 712 | 0 |  | | TCTGGTTTCTCTCGG |  |
| MGC33894 | 17 | 21087329 | ref | G | | G | FS | | 231 | 76 | A | | A |  |
| MGC34821 | 11 | 62605062 | snp | A | | C | NS | | 1944 | 500 | Y | | * |  |
| MGC34829 | 17 | 39787822 | del | C | |  | FS | | 1575 | 525 | E | | S |  |
| MIER2 | 19 | 257684 | ref | AGGAGTCA | | AGGAGTCA | N | | 1644 | 545 | * | | P |  |
| MLL3 | 7 | 151505855 | snp | G | | A | NS | | 7833 | 2538 | Q | | * |  |
| MLL4 | 19 | 40906473 | ins |  | | G | FS | | 3059 | 1019 | G | | G |  |
| MMEL1 | 1 | 2512269 | ref | CAGCGCGGCAGGGCCTTGGCTACCACACGCG | | CAGCGCGGCAGGGCCTTGGCTACCACACGCG | N | | 2490 | 767 | RVW* | | PAAL |  |
| MMP16 | 8 | 89150851 | ins |  | | A | FS | | 1519 | 412 | L | | L |  |
| MOCS2 | 5 | 52441301 | ref | GCACAGCGGCACCATC | | GCACAGCGGCACCATC | MS | | 39 | 0 | MVPLCQ | | DGAAVQ |  |
| MPHOSPH6 | 16 | 80739824 | ref | AAGGCATCCATCTTAATCCTGGGGCT | | AAGGCATCCATCTTAATCCTGGGGCT | N | | 518 | 156 | KPQD* | | KDGCL |  |
| MPZL3 | 11 | 117628205 | ref | CCTCTCTGCTGCATCCCGGC | | CCTCTCTGCTGCATCCCGGC | MS | | 2 | 0 | MQQRGAA | | GCSREAA |  |
| MRGPRG | 11 | 3198079 | snp | A | | G | MS | | 1 | 0 | M | | T |  |
| MRPL16 | 11 | 59334684 | ref | GCGCGAGCCAGCAGCCTCCACATGGT | | GCGCGAGCCAGCAGCCTCCACATGGT | MS | | 214 | 0 | MWRLLARAS | | TMWRLLARS |  |
| MRPL43 | 10 | 102737179 | ref | CGCGCCGTCATAGCTACAGCT | | CGCGCCGTCATAGCTACAGCT | MS | | 72 | 0 | MTARGTP | | SCSYGTP |  |
| MS4A12 | 11 | 60021577 | snp | C | | T | NS | | 267 | 70 | Q | | * |  |
| MS4A14 | 11 | 59921928 | del | TT | |  | FS | | 731 | 55 | I | | S |  |
| MSL3L2 | 2 | 234440306 | ins |  | | TCCA | FS | | 547 | 182 | V | | VD |  |
| MTMR2 | 11 | 95296759 | ref | TCTCCATC | | TCTCCATC | MS | | 153 | 0 | MEK | | DGE |  |
| MUC19 | 12 | 39106474 | snp | G | | A | D | | 561 | 62 |  | | TGCTTTATTTTGTAA |  |
| MUC2 | 11 | 1089217 | ref | GTGCCCA | | GTGCCCA | FS | | 6922 | 2298 | TPT | | SAQ |  |
| MUC4 | 3 | 197001272 | snp | T | | C | FS | | 2048 | 529 | A | | G |  |
| MUPCDH | 11 | 614888 | ref | GCCCAAGACCCCATCTTGGCGGCT | | GCCCAAGACCCCATCTTGGCGGCT | MS | | 105 | 0 | MGSWALLW | | SRQDGLLW |  |
| MYBBP1A | 17 | 4388981 | ref | CTTTATTT | | CTTTATTT | N | | 4055 | 1331 | K* | | IK |  |
| MYEF2 | 15 | 46230982 | ref | CAAAGCCT | | CAAAGCCT | NS | | 1401 | 425 | RGFG | | QRL* |  |
| MYL7 | 7 | 44147424 | ref | ACCATTCTCTCTGCAGAGGTGTGGC | | ACCATTCTCTCTGCAGAGGTGTGGC | MS | | 12 | 0 | MASRKAGTR | | SASRKAGTR |  |
| MYO15B | 17 | 71114680 | snp | T | | C | D | | 4166 | 0 |  | | GCGACA |  |
| MYO1F | 19 | 8548192 | ref | TGGTGGGGGGCTGGTGTCTGGGCTCCTGGAGGCTCCTGAATGGG | | TGGTGGGGGGCTGGTGTCTGGGCTCCTGGAGGCTCCTGAATGGG | MS | | 114 | 0 | MGSKERFHWQSHNVK | | LGSKERFHWQSHNVK |  |
| NACA2 | 17 | 57022734 | snp | G | | A | NS | | 614 | 196 | R | | * |  |
| NAG | 2 | 15224619 | ref | CCCTC | | CCCTC | N | | 7140 | 2371 | * | | W |  |
| NARS2 | 11 | 77963158 | ref | AGCAGGCAGCGGACCCCCAGCATCCCGCGTCCGCCCAGGCCCTCCGC | | AGCAGGCAGCGGACCCCCAGCATCCCGCGTCCGCCCAGGCCCTCCGC | MS | | 186 | 0 | MLGVRCLLRSVRFCSS | | AEGLGGRGRSVRFCSS |  |
| NAT8B | 2 | 73781438 | ref | A | | A | NS | | 536 | 167 | Q | | * |  |
| NCRNA00119 | 3 | 133929025 | snp | G | | T | D | | 38 | 0 |  | | TATTTTTTTTTTCAT |  |
| NEFL | 8 | 24866983 | del | G | |  | FS | | 1507 | 470 | P | | P |  |
| NEK9 | 14 | 74627822 | ins |  | | T | FS | | 2357 | 781 | M | | I |  |
| NEURL2 | 20 | 43953022 | ref | GGAGGCAGCAGCCATCT | | GGAGGCAGCAGCCATCT | MS | | 271 | 0 | MAAASE | | RWLLPE |  |
| NHEJ1 | 2 | 219649345 | ref | ATTAAC | | ATTAAC | N | | 977 | 298 | S* | | IN |  |
| NINJ1 | 9 | 94926992 | ref | CCAGCTCACCTGGGTGTCCTACTG | | CCAGCTCACCTGGGTGTCCTACTG | N | | 524 | 151 | Q* | | TQ |  |
| NIPSNAP1 | 22 | 28307069 | ref | ATGTTGGAGCCGC | | ATGTTGGAGCCGC | MS | | 254 | 0 | MAPRL | | AAPRL |  |
| NKG7 | 19 | 56567589 | ref | GCAGAGCTCCATGG | | GCAGAGCTCCATGG | MS | | 171 | 0 | MELCR | | PWSSR |  |
| NKX1-2 | 10 | 126128470 | ref | GCCTTGGCCCCGCCGTCCTGCCATGCCAGCATGC | | GCCTTGGCCCCGCCGTCCTGCCATGCCAGCATGC | MS | | 32 | 0 | MLAWQDGGAKAA | | ACWHGRTAGPRA |  |
| NKX2-1 | 14 | 36059082 | ref | CATCGGGCTT | | CATCGGGCTT | MS | | 82 | 0 | MWSG | | KWSG |  |
| NKX6-1 | 4 | 85638398 | ref | CTAACATCCCACG | | CTAACATCCCACG | MS | | 6 | 0 | MLAVG | | RGTVG |  |
| NKX6-3 | 8 | 41623114 | ref | ACGGCGGGCGTCAGACGCTGTGCGCTCCCAGGCTGAGC | | ACGGCGGGCGTCAGACGCTGTGCGCTCCCAGGCTGAGC | N | | 384 | 126 | VLSLGAHSV* | | VERTASDARR |  |
| NLRP6 | 11 | 270815 | ref | AT | | AT | FS | | 1081 | 360 | F | | Y |  |
| NLRP8 | 19 | 61191090 | snp | G | | C | N | | 3217 | 1048 | * | | Y |  |
| NME6 | 3 | 48317801 | ref | GTCATTCGGAGCCCC | | GTCATTCGGAGCCCC | MS | | 46 | 0 | MTQNL | | GAQNL |  |
| NMRAL1 | 16 | 4451780 | ref | GTC | | GTC | N | | 1279 | 299 | * | | Y |  |
| NOP14 | 4 | 2934843 | ref | TGGCGC | | TGGCGC | MS | | 72 | 0 | MA | | VA |  |
| NPAL2 | 8 | 99274787 | snp | C | | T | D | | 1322 | 355 |  | | ATGAGT |  |
| NPHP4 | 1 | 5857748 | snp | A | | T | NS | | 3081 | 938 | L | | * |  |
| NPSR1 | 7 | 34884264 | snp | C | | T | NS | | 1205 | 359 | R | | * |  |
| NR1I3 | 1 | 159467659 | snp | C | | T | D | | 756 | 202 |  | | GGCTCCTAATCCTAA |  |
| NR2C1 | 12 | 93948519 | ref | AGGTGAT | | AGGTGAT | N | | 1611 | 467 | * | | Y |  |
| NRXN1 | 2 | 51108895 | ref | TGGAGCAGCGCCGTCCCCATGCTCG | | TGGAGCAGCGCCGTCCCCATGCTCG | MS | | 1477 | 0 | MGTALLQRG | | RAWGRRWRG |  |
| NSMCE1 | 16 | 27143976 | ref | GGCTAA | | GGCTAA | N | | 895 | 265 | H* | | QA |  |
| NUDCD3 | 7 | 44496718 | ref | CTCCATGTC | | CTCCATGTC | MS | | 186 | 0 | MET | | DMT |  |
| NUDT21 | 16 | 55024119 | ref | GGAATTCAGTTGTAAATA | | GGAATTCAGTTGTAAATA | N | | 818 | 223 | FIYN* | | LQLNS |  |
| NUDT5 | 10 | 12249704 | ref | GCTT | | GCTT | N | | 1074 | 219 | * | | C |  |
| OAS1 | 12 | 111841575 | snp | G | | A | D | | 1144 | 346 |  | | GTCTCACCCTTTCAA |  |
| OAS2 | 12 | 111932670 | snp | A | | G | N | | 2298 | 719 | * | | W |  |
| ODF2L | 1 | 86587017 | ref | AATTTATTCAAACATTGTTACATAAGAAGTTTTAAGAAACTGTAAAT | | AATTTATTCAAACATTGTTACATAAGAAGTTTTAAGAAACTGTAAAT | NS | | 2105 | 499 | YLQFLKTSYVTMFE* | | LQFLKTSYVTMFE* |  |
| ODF3B | 22 | 49315771 | ref | GGGT | | GGGT | N | | 898 | 253 | * | | C |  |
| OR10X1 | 1 | 156816115 | snp | C | | T | NS | | 197 | 65 | W | | * |  |
| OR11G2 | 14 | 19736021 | ins |  | | A | FS | | 687 | 229 | G | | R |  |
| OR1B1 | 9 | 124431061 | snp | G | | A | NS | | 573 | 191 | R | | * |  |
| OR1S1 | 11 | 57739769 | snp | A | | G | N | | 977 | 325 | * | | W |  |
| OR2B11 | 1 | 245681885 | del | A | |  | FS | | 21 | 7 | F | | S |  |
| OR2L8 | 1 | 246179648 | snp | T | | A | NS | | 866 | 288 | Y | | * |  |
| OR2T4 | 1 | 246592261 | del | A | |  | FS | | 756 | 252 | I | | S |  |
| OR4C3 | 11 | 48303589 | snp | G | | A | NS | | 521 | 173 | W | | * |  |
| OR4L1 | 14 | 19598290 | del | TAGATTTGCTCACTGACCA |  | | FS | | 247 | 82 | IDLLTDH | | T | |
| OR4X1 | 11 | 48242806 | snp | T | | A | NS | | 818 | 272 | Y | | * |  |
| OR51I2 | 11 | 5432009 | ins |  | | CA | FS | | 715 | 238 | N | | T |  |
| OR52B4 | 11 | 4345982 | del | G | |  | FS | | 118 | 39 | A | | A |  |
| OR52E8 | 11 | 5834554 | snp | T | | A | N | | 953 | 317 | * | | Y |  |
| OR52N4 | 11 | 5733059 | snp | A | | T | NS | | 513 | 171 | R | | * |  |
| OR5AC2 | 3 | 99289633 | snp | T | | C | N | | 927 | 309 | * | | Q |  |
| OR5K2 | 3 | 99699867 | snp | T | | A | NS | | 653 | 217 | Y | | * |  |
| OR5K3 | 3 | 99593103 | ins |  | | A | FS | | 904 | 301 | I | | N |  |
| OR5K4 | 3 | 99556287 | del | A | |  | FS | | 900 | 300 | I | | L |  |
| OR5M1 | 11 | 56137128 | del | GACA | |  | FS | | 422 | 140 | CV | | C |  |
| OR6C1 | 12 | 54000674 | ins |  | | A | FS | | 24 | 8 | E | | R |  |
| OR6C74 | 12 | 53927521 | snp | C | | T | NS | | 183 | 61 | R | | * |  |
| OR6M1 | 11 | 123181837 | sub | GAA | | C | FS | | 427 | 142 | VL | | G |  |
| OR6Q1 | 11 | 57555684 | del | C | |  | FS | | 684 | 228 | L | | C |  |
| OR7G3 | 19 | 9097705 | ins |  | | GGTAT | FS | | 921 | 307 | I | | IP |  |
| OR8G1 | 11 | 123626408 | ref | G | | G | NS | | 776 | 258 | Y | | * |  |
| OR8K1 | 11 | 55870091 | snp | T | | C | MS | | 1 | 0 | M | | T |  |
| OR9K2 | 12 | 53809856 | del | T | |  | FS | | 37 | 12 | L | | C |  |
| OSM | 22 | 28992786 | ref | ATGCTGGGTGCCCGTGCTCCGGCC | | ATGCTGGGTGCCCGTGCTCCGGCC | MS | | 52 | 0 | MGVLLTQR | | GGVLLTQR |  |
| OVCH2 | 11 | 7669046 | snp | C | | T | NS | | 1667 | 555 | W | | * |  |
| OXTR | 3 | 8784859 | ref | AGCGCGCCCTCCATGACCCTGGCGGCAGCGGTGCGCC | | AGCGCGCCCTCCATGACCCTGGCGGCAGCGGTGCGCC | MS | | 622 | 0 | MEGALAANWSAEA | | GAPLPAANWSAEA |  |
| P2RX5 | 17 | 3541029 | del | G | |  | FS | | 612 | 109 | T | | T |  |
| PABPC3 | 13 | 24569097 | ins |  | | T | FS | | 822 | 253 | K | | N |  |
| PADI6 | 1 | 17591260 | del | G | |  | FS | | 1026 | 342 | V | | W |  |
| PALM | 19 | 678549 | - | TCCCTGCTGCTCA | | CCTCT? | D | | 332 | 46 |  | | ACCTCT?G |  |
| PARD3B | 2 | 205873509 | del | A | |  | FS | | 2217 | 670 | K | | K |  |
| PATZ1 | 22 | 30052859 | ref | GGGACACAGCAGCTGCCT | | GGGACACAGCAGCTGCCT | N | | 2724 | 687 | * | | C |  |
| PAX8 | 2 | 113710769 | ins |  | | C | D | | 943 | 259 |  | | CTCTTTGCTCCCCAGG |  |
| PBOV1 | 6 | 138580879 | ins |  | | G | FS | | 441 | 115 | T | | T |  |
| PBX2 | 6 | 32265666 | ref | CCATAGCT | | CCATAGCT | MS | | 271 | 0 | MDE | | SYE |  |
| PCDHA4 | 5 | 140167162 | sub | GGGCCG | | AAGACAC | FS | | 307 | 68 | KGR | | KRH |  |
| PCDHB9 | 5 | 140548219 | ins |  | | A | FS | | 1144 | 381 | I | | N |  |
| PCSK4 | 19 | 1432750 | ref | GACAACTTCAGGTTCCAGCTGGCAG | | GACAACTTCAGGTTCCAGCTGGCAG | N | | 2312 | 750 | LPAGT* | | LEPEVV |  |
| PDE4DIP | 1 | 143626917 | snp | G | | A | NS | | 2154 | 621 | R | | * |  |
| PDZD7 | 10 | 102779948 | ref | TGCGAAACCCTGCGCCATGGCGGCTGGGCTAGG | | TGCGAAACCCTGCGCCATGGCGGCTGGGCTAGG | MS | | 215 | 0 | MAQGFAVGFDP | | PSPAAMVGFDP |  |
| PDZD8 | 10 | 119032757 | ref | AGACCTGTCTGCTACAC | | AGACCTGTCTGCTACAC | N | | 3658 | 1153 | V* | | RS |  |
| PEBP4 | 8 | 22626814 | ref | CAAAGCCGGCTATCTAGCAGG | | CAAAGCCGGCTATCTAGCAGG | N | | 768 | 225 | AC* | | AAL |  |
| PECAM1 | 17 | 59801595 | ref | CGACAGA | | CGACAGA | FS | | 912 | 233 | STP | | SVA |  |
| PEX10 | 1 | 2327055 | ref | GGCGCCGGCTCA | | GGCGCCGGCTCA | N | | 1047 | 326 | * | | A |  |
| PEX11G | 19 | 7459892 | ref | CCATGGCAACTCCGTGACGTCACCGCG | | CCATGGCAACTCCGTGACGTCACCGCG | MS | | 9 | 0 | MASLSGLAS | | GASLSGLAS |  |
| PFDN2 | 1 | 159354439 | ref | TCTTC | | TCTTC | MS | | 50 | 0 | MA | | VA |  |
| PFKP | 10 | 3139401 | - | ACAGAACC | | G? | D | | 810 | 258 |  | | TTTCTGTCTCCG? |  |
| PIGN | 18 | 57956511 | snp | T | | C | D | | 1487 | 372 |  | | TACTTGGTTCCCAGG |  |
| PIK3R6 | 17 | 8665941 | ins |  | | G | FS | | 2065 | 608 | S | | S |  |
| PKD1L2 | 16 | 79789760 | - | CATGTACCTTCAACCG | | ?CA | D | | 1543 | 514 |  | | TG? |  |
| PLA2G12A | 4 | 110870412 | ref | ATG | | ATG | MS | | 246 | 0 | M | | Q |  |
| PLA2G2D | 1 | 20313190 | ref | CTTCTAG | | CTTCTAG | N | | 451 | 144 | C* | | WK |  |
| PLCD3 | 17 | 40548073 | ref | GTC | | GTC | FS | | 1735 | 540 | RT | | RP |  |
| PLIN | 15 | 88009809 | ref | GCGGCGACTCAGC | | GCGGCGACTCAGC | N | | 1688 | 521 | S* | | SR |  |
| PMEPA1 | 20 | 55668099 | ref | CCACCACCATCACCATCATC | | CCACCACCATCACCATCATC | MS | | 144 | 0 | MMVMVVV | | DDGDGGV |  |
| PNKP | 19 | 55062239 | ref | GCCACAAGCGGCCCGGGGCCTCCACCTCGCCCATCCTG | | GCCACAAGCGGCCCGGGGCCTCCACCTCGCCCATCCTG | MS | | 106 | 0 | MGEVEAPGRLWLE | | QDGRGGGPGPLVE |  |
| PNLIPRP2 | 10 | 118373439 | ref | ACCCTAGGAAAAGAG | | ACCCTAGGAAAAGAG | FS | | 75 | 17 | GKEVCY | | GKESAT |  |
| POLR2E | 19 | 1046312 | ref | ATGGCAGCCTCCG | | ATGGCAGCCTCCG | MS | | 22 | 0 | MDDEE | | RDDEE |  |
| POLR3K | 16 | 37426 | ref | GCCC | | GCCC | N | | 365 | 108 | * | | Y |  |
| POU5F1 | 6 | 31241921 | ref | C | | C | MS | | 84 | 0 | M | | R |  |
| PPFIA2 | 12 | 80179882 | ref | AGTGGCTGGT | | AGTGGCTGGT | N | | 3934 | 1257 | * | | C |  |
| PPIAP19 | 10 | 116439871 | ref | AGATAAAACACAAGTCAAACTTATT | | AGATAAAACACAAGTCAAACTTATT | N | | 679 | 226 | E* | | VS |  |
| PPP2R1B | 11 | 111142293 | ref | ATGTTCTTTCTCCTCC | | ATGTTCTTTCTCCTCC | MS | | 84 | 0 | MAGASE | | GAGASE |  |
| PRAM1 | 19 | 8473464 | ref | TGATGGGCCATGGGATGAGTGGGACCCGAGCTGGGGCCG | | TGATGGGCCATGGGATGAGTGGGACCCGAGCTGGGGCCG | MS | | 20 | 0 | MAHHLPAAMESHQ | | ARVPLPAAMESHQ |  |
| PRB4 | 12 | 11353068 | snp | G | | A | NS | | 148 | 38 | R | | * |  |
| PRIM2 | 6 | 57506149 | ins |  | | A | FS | | 980 | 297 | N | | K |  |
| PSAPL1 | 4 | 7487446 | ref | AGGTGGGGCTG | | AGGTGGGGCTG | FS | | 144 | 16 | ASPTS | | AAPPQ |  |
| PSD | 10 | 104166764 | ref | GCGCATGGCACCCTGGGCCATGCTGGGGCCGGGGGTCAGGCT | | GCGCATGGCACCCTGGGCCATGCTGGGGCCGGGGGTCAGGCT | MS | | 527 | 0 | MAQGAMRFCSEGDC | | SLTPGPSFCSEGDC |  |
| PSG2 | 19 | 48278560 | ref | TG | | TG | MS | | 93 | 0 | M | | L |  |
| PTCHD3 | 10 | 27727230 | snp | A | | G | N | | 2343 | 767 | * | | Q |  |
| PTPN22 | 1 | 114159033 | ref | TAAATCTGGAGTTTTATTAA | | TAAATCTGGAGTTTTATTAA | N | | 2509 | 806 | I* | | IL |  |
| RAB11FIP2 | 10 | 119795659 | ref | ATCATCCTGTCCTGTTTCTCTGCCCC | | ATCATCCTGTCCTGTTTCTCTGCCCC | MS | | 440 | 0 | MMLSEQAQK | | GALSEQAQK |  |
| RAB1A | 2 | 65210548 | ref | GACATGT | | GACATGT | MS | | 240 | 0 | MSS | | TCS |  |
| RAD54L | 1 | 46499090 | sub | AC | | TA | NS | | 1243 | 194 | T | | * |  |
| RAPGEF6 | 5 | 130790851 | ref | CAAAGGCT | | CAAAGGCT | N | | 5010 | 1601 | * | | L |  |
| RASSF1 | 3 | 50353235 | ref | GACATGGCCCGGTTGGGCCCGTG | | GACATGGCCCGGTTGGGCCCGTG | MS | | 131 | 0 | MSGEPELI | | HGGEPELI |  |
| REXO4 | 9 | 135272784 | ref | TCCTGCTGCCG | | TCCTGCTGCCG | MS | | 200 | 0 | MGKA | | LGKA |  |
| RFPL1 | 22 | 28167883 | snp | C | | T | NS | | 935 | 242 | Q | | * |  |
| RGL2 | 6 | 33374355 | ref | GCGGGAGCATGGCCG | | GCGGGAGCATGGCCG | MS | | 133 | 0 | MLPRP | | RPCWP |  |
| RHBG | 1 | 154620978 | del | C | |  | FS | | 1309 | 423 | P | | P |  |
| RICTOR | 5 | 38978160 | ref | GGTC | | GGTC | N | | 5149 | 1708 | * | | S |  |
| RNF112 | 17 | 19256673 | ref | G | | G | FS | | 564 | 121 | L | | L |  |
| RNF166 | 16 | 87291455 | ref | GGCGGGTGGCTGCGCTTCCCTTCAG | | GGCGGGTGGCTGCGCTTCCCTTCAG | N | | 784 | 236 | N* | | NA |  |
| RNF213 | 17 | 75928747 | ins |  | | T | FS | | 427 | 68 | V | | C |  |
| RNF39 | 6 | 30146848 | ref | TCTAGTTGGAGACGAGACTCAGC | | TCTAGTTGGAGACGAGACTCAGC | N | | 1320 | 419 | S* | | TR |  |
| RPAP1 | 15 | 39616579 | ref | CACCTCGGACTCCCCTGGCTTCGGTCTCGACAGCATCTTGC | | CACCTCGGACTCCCCTGGCTTCGGTCTCGACAGCATCTTGC | MS | | 124 | 0 | MLSRPKPGESEVDL | | ARCCRDRSQGSPDL |  |
| RPS25 | 11 | 118394186 | ref | ACCCCTGAAGCTTACCATTGCGAAGCT | | ACCCCTGAAGCTTACCATTGCGAAGCT | MS | | 63 | 0 | MPPKDDKKK | | SPPKDDKKK |  |
| S1PR5 | 19 | 10486670 | ref | AGCAGCCCCGACTCCATGGG | | AGCAGCCCCGACTCCATGGG | MS | | 57 | 0 | MESGLLR | | PMESGLR |  |
| SALL4 | 20 | 49852336 | ref | CTGCTTGCGCCTCGACATGGTGCGAGC | | CTGCTTGCGCCTCGACATGGTGCGAGC | MS | | 67 | 0 | MSRRKQAKP | | ARTMSRAKP |  |
| SARM1 | 17 | 23732426 | sub | TGGGT | | CGTGG | FS | | 615 | 182 | SVA | | SVE |  |
| SCAMP1 | 5 | 77781608 | ref | C | | C | FS | | 885 | 242 | G | | G |  |
| SCAPER | 15 | 74874837 | - | TAT | | ?TAC? | D | | 644 | 202 |  | | TATTTTTCTCC?GTA?G |  |
| SCD5 | 4 | 83771486 | ref | GGATGGCTGTTCCAAGTTCAAGCACTGCTGTCTC | | GGATGGCTGTTCCAAGTTCAAGCACTGCTGTCTC | N | | 1296 | 325 | GDSSA* | | DLEQPS |  |
| SEC22B | 1 | 143823776 | snp | C | | T | NS | | 533 | 131 | R | | * |  |
| SEC24C | 10 | 75199173 | sub | AA | | G | FS | | 2666 | 829 | N | | A |  |
| SEMA3B | 3 | 50281761 | ins |  | | C | FS | | 328 | 28 | P | | P |  |
| SEP15 | 1 | 87101804 | ref | AATGCTCAGTTCTTCAGCA | | AATGCTCAGTTCTTCAGCA | N | | 682 | 123 | C* | | SI |  |
| SEPHS2 | 16 | 30364547 | ref | ATGGCGCCTGCCCGG | | ATGGCGCCTGCCCGG | MS | | 176 | 0 | MAEAS | | PAEAS |  |
| SERPINB11 | 18 | 59530817 | ref | T | | T | NS | | 329 | 89 | E | | * |  |
| SF1 | 11 | 64289861 | ref | AAGTC | | AAGTC | N | | 2296 | 639 | * | | Y |  |
| SF3A1 | 22 | 29082766 | ref | GGGTCCGGCCGGCATGACTGCGACGCTCAG | | GGGTCCGGCCGGCATGACTGCGACGCTCAG | MS | | 132 | 0 | MPAGPVQAVP | | LSVAVVQAVP |  |
| SF3B1 | 2 | 197991886 | ref | CAGCAGTTCTGACTTCAAGCAGCAGAATAGAAGCCTAGAAAATT | | CAGCAGTTCTGACTTCAAGCAGCAGAATAGAAGCCTAGAAAATT | N | | 463 | 138 | GFYSAA* | | GLKSELL |  |
| SFRS2B | 11 | 94440548 | ref | AG | | AG | FS | | 845 | 170 | S | | S |  |
| SGEF | 3 | 155440986 | del | GTCA | |  | FS | | 2394 | 742 | HL | | H |  |
| SH2B2 | 7 | 101741171 | snp | G | | C | D | | 1071 | 312 |  | | TCAAGCTCACTGCAC |  |
| SH3YL1 | 2 | 232799 | snp | T | | C | MS | | 568 | 0 | M | | V |  |
| SHF | 15 | 43247434 | ref | ATCTA | | ATCTA | N | | 1764 | 423 | * | | D |  |
| SIGLEC11 | 19 | 55156104 | ref | GCAGCAGCATCTCTGGGCTCTGGGGCT | | GCAGCAGCATCTCTGGGCTCTGGGGCT | MS | | 127 | 0 | MLLLPLLLP | | SPRVPLLLP |  |
| SIGLEC12 | 19 | 56696606 | sub | G | | CT | FS | | 248 | 64 | R | | R |  |
| SLC15A4 | 12 | 127874435 | ref | CTCCATGCGACG | | CTCCATGCGACG | MS | | 53 | 0 | MEGS | | RRGS |  |
| SLC16A12 | 10 | 91182799 | ref | GTCATGTGAGGC | | GTCATGTGAGGC | N | | 1841 | 483 | SLT* | | TSHD |  |
| SLC19A2 | 1 | 167701699 | ref | TCAGCAGTATATTATGA | | TCAGCAGTATATTATGA | NS | | 1725 | 496 | S* | | C* |  |
| SLC22A10 | 11 | 62814500 | snp | G | | A | NS | | 495 | 95 | W | | * |  |
| SLC24A1 | 15 | 63704673 | del | C | |  | FS | | 1488 | 400 | P | | Q |  |
| SLC25A29 | 14 | 99828368 | ref | AGCGTCACAGGCTGG | | AGCGTCACAGGCTGG | N | | 1192 | 300 | SSL* | | SCDA |  |
| SLC25A30 | 13 | 44883540 | ref | TTCCAGTTGAGGGCTGACATT | | TTCCAGTTGAGGGCTGACATT | MS | | 136 | 0 | MSALNWK | | NVSPQLE |  |
| SLC26A6 | 3 | 48647822 | ref | AGCCCCATGGCTCGCAAGTTG | | AGCCCCATGGCTCGCAAGTTG | MS | | 100 | 0 | MGLADAS | | QLAADAS |  |
| SLC2A12 | 6 | 134353980 | ref | GGCCATTAGGT | | GGCCATTAGGT | N | | 2019 | 616 | T* | | MA |  |
| SLC38A3 | 3 | 50226839 | ins |  | | G | FS | | 244 | 34 | V | | G |  |
| SLC6A18 | 5 | 1293756 | snp | C | | G | NS | | 1079 | 318 | Y | | * |  |
| SLC7A5 | 16 | 86424050 | ref | CCTCCGGCAGCCACTCGGCCTCCTGGCTATGTCTCCTGG | | CCTCCGGCAGCCACTCGGCCTCCTGGCTATGTCTCCTGG | N | | 1583 | 503 | PQET* | | PGCRR |  |
| SLC9A1 | 1 | 27300900 | - | GCAGCTGT | | ?TGC | D | | 2511 | 644 |  | | CTGTCCCTCCCGCA? |  |
| SLC9A11 | 1 | 171736854 | ref | TTCAA | | TTCAA | N | | 3741 | 1123 | S* | | SE |  |
| SLIT1 | 10 | 98750853 | ref | CAGCGCT | | CAGCGCT | N | | 4835 | 1534 | * | | L |  |
| SMPD4 | 2 | 130655640 | ref | TCATCAAGCTGCGCGCAG | | TCATCAAGCTGCGCGCAG | MS | | 520 | 0 | MTTFGA | | LPTFGA |  |
| SMTN | 22 | 29816137 | ref | CAGAGGCC | | CAGAGGCC | FS | | 1055 | 279 | RGP | | QRP |  |
| SOCS1 | 16 | 11256183 | ref | ACGGCGGGCGCTGCCGGTCAAATCTGGAAG | | ACGGCGGGCGCTGCCGGTCAAATCTGGAAG | N | | 777 | 207 | PFQI* | | PSARR |  |
| SON | 21 | 33870555 | ins |  | | A | FS | | 7285 | 2412 | A | | S |  |
| SOX21 | 13 | 94161469 | ref | GGGGTCATAG | | GGGGTCATAG | N | | 911 | 275 | L* | | DP |  |
| SPATA4 | 4 | 177343004 | del | CTCT | |  | FS | | 943 | 278 | RE | | K |  |
| SPATA6 | 1 | 48710263 | ref | GCATCCGT | | GCATCCGT | MS | | 165 | 0 | MPK | | TAK |  |
| SPON1 | 11 | 14058070 | ins |  | | C | FS | | 1141 | 201 | C | | L |  |
| SPTLC2 | 14 | 77152656 | ref | CTCCGGGCTCCGGCCGCATCTTCC | | CTCCGGGCTCCGGCCGCATCTTCC | MS | | 188 | 0 | MRPEPGGC | | GRCGRSRC |  |
| SRD5A2 | 2 | 31659375 | ref | GTAGCCGGAGG | | GTAGCCGGAGG | FS | | 159 | 29 | PSGY | | PPAT |  |
| SSPO | 7 | 149107603 | ins |  | | C | FS | | 1120 | 373 | L | | P |  |
| SSX2IP | 1 | 84909470 | ref | ACAGTCATCCAATCTCCCATAG | | ACAGTCATCCAATCTCCCATAG | MS | | 266 | 0 | MGDWMTVT | | LWEIG* |  |
| ST8SIA5 | 18 | 42514000 | ref | CATCAGCAGCAGCTGCAGGTGCCCGTGTGCACGC | | CATCAGCAGCAGCTGCAGGTGCCCGTGTGCACGC | N | | 1667 | 366 | RVHTGTCSCC* | | RAHGHLQLLLM |  |
| STK25 | 2 | 242096122 | ref | TGG | | TGG | MS | | 215 | 0 | M | | L |  |
| STX1B | 16 | 30911640 | ref | GCCTACAAGCCCAGCGT | | GCCTACAAGCCCAGCGT | N | | 910 | 284 | TLGL* | | AGLVG |  |
| SURF6 | 9 | 135188521 | ref | AGACT | | AGACT | N | | 1163 | 361 | * | | C |  |
| SYT13 | 11 | 45264329 | ref | ACCATGGTGCCC | | ACCATGGTGCCC | MS | | 49 | 0 | MVLS | | GQLS |  |
| SYT17 | 16 | 19102436 | snp | C | | T | NS | | 748 | 139 | Q | | * |  |
| TAP2 | 6 | 32904605 | ref | GGCCT | | GGCCT | N | | 2233 | 703 | * | | C |  |
| TAS2R48 | 12 | 11065552 | snp | C | | T | NS | | 884 | 294 | W | | * |  |
| TBC1D16 | 17 | 75529250 | ref | ACCT | | ACCT | N | | 2418 | 767 | * | | C |  |
| TCEAL6 | X | 101282439 | ins |  | | G | FS | | 681 | 173 | A | | A |  |
| TCERG1 | 5 | 145866923 | ins |  | | A | FS | | 2847 | 935 | K | | K |  |
| TCF7L2 | 10 | 114902111 | del | AAATACTACGAGCTGGCCCG | |  | FS | | 1427 | 374 | KYYELAR | | E | |
| TDH | 8 | 11256302 | snp | A | | G | D | | 384 | 0 |  | | TTATGTTCCTTGCGG |  |
| TFB1M | 6 | 155620654 | ref | GCAGCAGCTAGAGTCTGTAATTCTC | | GCAGCAGCTAGAGTCTGTAATTCTC | N | | 1087 | 341 | ENYRL* | | TDSSCC |  |
| TFPI2 | 7 | 93357902 | ref | CCCCAGGGGGCGAGCGGGGTCCATGGTGCAGGGGGTCGGGCGGCCC | | CCCCAGGGGGCGAGCGGGGTCCATGGTGCAGGGGGTCGGGCGGCCC | MS | | 75 | 0 | MDPARPLGLSILLLFL | | GPPDPLHHLSILLLFL |  |
| TG | 8 | 133994538 | snp | C | | T | NS | | 4265 | 1408 | Q | | * |  |
| TGIF1 | 18 | 3442222 | del | T | |  | FS | | 632 | 81 | P | | P |  |
| TIGD6 | 5 | 149355073 | del | T | |  | FS | | 1292 | 343 | Q | | Q |  |
| TLR3 | 4 | 187242321 | snp | T | | A | D | | 2587 | 828 |  | | GAAGGT |  |
| TLR8 | X | 12834746 | snp | A | | G | MS | | 68 | 0 | M | | V |  |
| TM2D2 | 8 | 38972177 | ref | ATC | | ATC | MS | | 294 | 0 | M | | E |  |
| TM7SF3 | 12 | 27058337 | ref | CCGCTACGACCAGCAGCTGCAGGAACCCCATTGCTGCCTG | | CCGCTACGACCAGCAGCTGCAGGAACCCCATTGCTGCCTG | MS | | 37 | 0 | MGFLQLLVVAVLAS | | QAAMGFLQLLVLAS |  |
| TMED5 | 1 | 93418385 | ref | ATC | | ATC | MS | | 447 | 0 | M | | E |  |
| TMEM170A | 16 | 74038944 | ref | TAAGGATGTATGCTATAGAGTAGC | | TAAGGATGTATGCTATAGAGTAGC | N | | 509 | 141 | ATL* | | HTSL |  |
| TMEM216 | 11 | 60922307 | ins |  | | A | FS | | 683 | 82 | S | | K |  |
| TMPRSS11A | 4 | 68511703 | snp | C | | T | MS | | 123 | 0 | M | | I |  |
| TP53RK | 20 | 44751454 | ref | CGCCATGACTG | | CGCCATGACTG | MS | | 223 | 0 | MAAA | | QSAA |  |
| TPCN1 | 12 | 112192033 | snp | G | | A | D | | 1035 | 249 |  | | ATGAGT |  |
| TPCN2 | 11 | 68591543 | - | TA | | CN | D | | 842 | 242 |  | | CGCTTCTGCTCTCNG |  |
| TPTE2 | 13 | 18898610 | ref | TCATATACATTAATTAATA | | TCATATACATTAATTAATA | NS | | 1310 | 366 | VLINVYD | | IIN* |  |
| TRAK2 | 2 | 201953489 | ref | AGGTCAGTTAACTGCTGAACCTCAGTCCTCCTTC | | AGGTCAGTTAACTGCTGAACCTCAGTCCTCCTTC | NS | | 3113 | 910 | LKED* | | LVN* |  |
| TREH | 11 | 118034254 | ref | CCAGGCAGT | | CCAGGCAGT | FS | | 1741 | 565 | HCLA | | HCLG |  |
| TRPM1 | 15 | 29156414 | snp | A | | G | MS | | 129 | 0 | M | | T |  |
| TRPM7 | 15 | 48641159 | ref | ATTAATATTA | | ATTAATATTA | N | | 5859 | 1865 | * | | N |  |
| TRPV1 | 17 | 3423920 | ins |  | | G | FS | | 2385 | 619 | A | | A |  |
| TSC22D1 | 13 | 43906756 | ref | GGCAGCT | | GGCAGCT | N | | 648 | 144 | * | | S |  |
| TTC22 | 1 | 55039421 | ref | CATGAC | | CATGAC | MS | | 35 | 0 | MA | | VA |  |
| TTC32 | 2 | 19960216 | ref | AAGTTAAAAATATCAATA | | AAGTTAAAAATATCAATA | NS | | 582 | 150 | Y* | | * |  |
| TTC37 | 5 | 94826063 | ref | ATGTTA | | ATGTTA | N | | 4962 | 1564 | * | | H |  |
| TUBA1A | 12 | 47869025 | ref | CCATGG | | CCATGG | MS | | 99 | 0 | MR | | PR |  |
| TUBGCP2 | 10 | 134966430 | ref | CTCATAGTT | | CTCATAGTT | MS | | 357 | 0 | MSE | | NYE |  |
| TXNRD2 | 22 | 18244616 | ref | AGGGATGGCGCTTACCC | | AGGGATGGCGCTTACCC | N | | 1602 | 523 | G* | | IP |  |
| TYRO3 | 15 | 39649514 | snp | A | | T | D | | 1476 | 417 |  | | ACCTCCCTCCCACTG |  |
| UBE2CBP | 6 | 83832133 | ref | GTCTCCGCCGCAGAAGCCGCCATGGCAGGCTTCCAGTC | | GTCTCCGCCGCAGAAGCCGCCATGGCAGGCTTCCAGTC | MS | | 108 | 0 | MAASAAETRVFLE | | DWKPAMAARVFLE |  |
| UBE2V1 | 20 | 48163068 | ref | CCATCTTGCGTCGCT | | CCATCTTGCGTCGCT | MS | | 45 | 0 | MAATT | | SAATT |  |
| UBR4 | 1 | 19409306 | ref | TCTTCGCCGCCGCTCGTCGCCATCTTCCGTCGT | | TCTTCGCCGCCGCTCGTCGCCATCTTCCGTCGT | MS | | 4 | 0 | MATSGGEEAAA | | EDGDERRRAAA |  |
| UBXN1 | 11 | 62202967 | ref | CCATGGCG | | CCATGGCG | MS | | 132 | 0 | MAE | | RPE |  |
| UBXN8 | 8 | 30740386 | ins |  | | T | FS | | 643 | 208 | L | | F |  |
| UCP2 | 11 | 73363815 | snp | T | | C | D | | 1194 | 271 |  | | ATTTTTCTCCTCTGG |  |
| UGT2A1 | 4 | 70547375 | snp | A | | T | NS | | 638 | 191 | Y | | * |  |
| UHRF1 | 19 | 4905679 | ins |  | | C | FS | | 2143 | 658 | A | | A |  |
| UNC93B1 | 11 | 67521739 | ins |  | | G | FS | | 968 | 296 | F | | L |  |
| UQCR | 19 | 1556407 | ref | TCGCGGCGGAGTCGCAC | | TCGCGGCGGAGTCGCAC | MS | | 23 | 0 | MVTRFL | | VVTRFL |  |
| USO1 | 4 | 76889984 | del | GT | |  | D | | 371 | 65 |  | | AATC |  |
| USP29 | 19 | 62334593 | snp | C | | A | NS | | 3094 | 912 | Y | | * |  |
| VAV3 | 1 | 108309000 | ref | TTCCACGGCTCCATGCCCGACGGCTCC | | TTCCACGGCTCCATGCCCGACGGCTCC | MS | | 54 | 0 | MEPWKQCAQ | | GAVGQQCAQ |  |
| VDR | 12 | 46559161 | snp | A | | G | MS | | 161 | 0 | M | | T |  |
| VPS11 | 11 | 118445151 | ins |  | | C | FS | | 265 | 74 | L | | P |  |
| VPS13B | 8 | 100202881 | snp | T | | G | NS | | 1349 | 412 | Y | | * |  |
| VRK3 | 19 | 55174160 | ref | ACCT | | ACCT | N | | 1769 | 474 | * | | C |  |
| VTA1 | 6 | 142561402 | ref | CACGCTCCAGCTAATACACCAGCAGAAGTGCCTCACAGCACAGG | | CACGCTCCAGCTAATACACCAGCAGAAGTGCCTCACAGCACAGG | NS | | 669 | 218 | HAPANTPAEVPHSTG | | PRSS* |  |
| WASF4 | X | 47542989 | snp | C | | T | NS | | 1098 | 42 | Q | | * |  |
| WDR51A | 3 | 52163409 | ref | CCGCGCAGGGCGCAGCCATGGCGGGGCTGGCG | | CCGCGCAGGGCGCAGCCATGGCGGGGCTGGCG | MS | | 32 | 0 | MAAPCAEDPSL | | RQPRHGEDPSL |  |
| WDR68 | 17 | 59014626 | del | G | |  | FS | | 776 | 186 | G | | V |  |
| WDR74 | 11 | 62363615 | ref | CATG | | CATG | MS | | 168 | 0 | MA | | HA |  |
| WHSC2 | 4 | 1980482 | ref | ATCT | | ATCT | MS | | 63 | 0 | MA | | RA |  |
| WNT16 | 7 | 120752706 | ins |  | | CCCA | FS | | 50 | 0 | M | | TH |  |
| XBP1 | 22 | 27522137 | ref | CT | | CT | FS | | 545 | 165 | AG | | AV |  |
| XKR5 | 8 | 6660787 | del | A | |  | FS | | 891 | 289 | S | | S |  |
| XPA | 9 | 99499386 | ref | CGCCGCCATCTC | | CGCCGCCATCTC | MS | | 117 | 0 | MAAA | | EMAA |  |
| ZACN | 17 | 71589391 | snp | C | | T | NS | | 877 | 279 | Q | | * |  |
| ZAN | 7 | 100209412 | ref |  | |  | FS | | 5933 | 1922 | C | | F |  |
| ZBTB45 | 19 | 63717211 | ref | CGAGGCCAGGCCCCAGCGCCATCAGGGCGCAG | | CGAGGCCAGGCCCCAGCGCCATCAGGGCGCAG | N | | 1817 | 508 | PAP* | | RLAS |  |
| ZC3H8 | 2 | 112729028 | ref | TGA | | TGA | MS | | 92 | 0 | M | | L |  |
| ZCCHC3 | 20 | 226690 | ref | CG | | CG | FS | | 484 | 153 | P | | R |  |
| ZFHX3 | 16 | 71378561 | ref | GCTTACAATCTGAAGGTGTCCGTTCCTACACTG | | GCTTACAATCTGAAGGTGTCCGTTCCTACACTG | N | | 11754 | 3693 | TSVGTDTFRL* | | TCRNGHLQIVS |  |
| ZNF117 | 7 | 64076101 | snp | G | | A | NS | | 2566 | 427 | R | | * |  |
| ZNF233 | 19 | 49470635 | sub | GTTG | | ATA | FS | | 2109 | 660 | SL | | SY |  |
| ZNF268 | 12 | 132289529 | ref | AGCTT | | AGCTT | FS | | 1514 | 394 | KAF | | KA? |  |
| ZNF274 | 19 | 63410173 | ins |  | | G | FS | | 690 | 72 | A | | G |  |
| ZNF428 | 19 | 48803605 | ref | TGCCTAC | | TGCCTAC | N | | 990 | 187 | V* | | VA |  |
| ZNF429 | 19 | 21512274 | del | A | |  | FS | | 1716 | 526 | H | | L |  |
| ZNF491 | 19 | 11778245 | snp | G | | T | NS | | 762 | 159 | E | | * |  |
| ZNF595 | 4 | 75997 | ref |  | |  | FS | | 759 | 201 | C | | V |  |
| ZNF653 | 19 | 11477598 | ref | CATC | | CATC | MS | | 53 | 0 | MA | | DA |  |
| ZNF681 | 19 | 23719583 | del | CA | |  | FS | | 748 | 202 | C | | W |  |
| ZNF747 | 16 | 30453480 | ref | CCCCAGCGACGGATCGGTCATCTCCCCTGGCCAGGCCTGGGCATGCGG | | CCCCAGCGACGGATCGGTCATCTCCCCTGGCCAGGCCTGGGCATGCGG | MS | | 194 | 0 | MTDPSLGLTVPMAPPL | | PHAQAWPLTVPMAPPL |  |
| ZNF770 | 15 | 33062919 | ref | GCCATCATATTCTTCAATGATATACTCTGAT | | GCCATCATATTCTTCAATGATATACTCTGAT | MS | | 311 | 0 | MMAENNLKMLK | | IRVENNLKMLK |  |
| ZNF806 | 2 | 132791950 | del | C | |  | FS | | 941 | 313 | P | | P |  |
| ZNF821 | 16 | 70451403 | ref | CAGGCAGGAGGGTGTGGTTCAGTGCAGAG | | CAGGCAGGAGGGTGTGGTTCAGTGCAGAG | N | | 1478 | 367 | SLH* | | SSCL |  |
| ZNF831 | 20 | 57199622 | ins |  | | C | FS | | 153 | 51 | T | | H |  |
| ZZZ3 | 1 | 77871610 | ref | GATCGGGAAGCAGCCATACTAT | | GATCGGGAAGCAGCCATACTAT | MS | | 476 | 0 | MAASRSTR | | IVWLLPTR |  |
