# Supplementary material for: A mononucleotide repeat in PRRT2 is an important, frequent target of mismatch repair deficiency in cancer
Source: Oncotarget. 2016 Nov 19;8(4):6043–56. doi: 10.18632/oncotarget.13464 (PMC5351611; doi:10.18632/oncotarget.13464)
Supplement: Supplementary file 3 [file oncotarget-08-6043-s003.doc]

Table S5) Mutation profiling of the selected gene panel in all colorectal, endometrial and ovarian cancer cell lines used to assess the mutation status. The MMR proficient cell lines are depicted in blue and the MMR deficient cells lines are in orange. ND represents not defined. WT represents the wildtype. M1, M2 and M3 correspond to deletion of one, two or three nucleotides, respectively. P1 and P2 correspond to insertion of one or two nucleotides.

|  | **Colorectal Cancer** | | | | | | | | | | | | |
| --- | --- | --- | --- | --- | --- | --- | --- | --- | --- | --- | --- | --- | --- |
| **Gene** | **CACO-2** | **HT-29** | **SW-480** | **HCT116** | **LOVO** | **LS174T** | **CO115** | **RKO** | **TC7** | **TC71** | **4224** | **5583S** | **DV-90** |
| PRRT2 | wt | wt | wt | wt | m1wtp1p2 | m1wt | m1wt | wt | wt | wt | wt | wt | m1wt |
| TSHZ2 | wt | wt | wt | m1wt | m1wt | m1wt | m1wt | m1wt | m1wt | m1wt | m1wt | m1wt | m1wt |
| ANLN | wt | wt | wt | wt | wt | wt | wt | wt | wt | wt | wt | wt | wt |
| CEP164 | wt | wt | wt | m1 | m1wt | m1wtp1 | m1 | m1 | m1wt | m1wt | wt | wt | m1wt |
| CNOT1 | wt | wt | ND | m1m2 | m1m2 | m1m2 | m1m2m3 | m1m2 | m1m2 | m1m2 | wt | m1m2 | m1m2 |
| KCNMA1 | wt | wt | wt | m1m2wt | m1m2wt | m1m2wt | m1m2wt | m1m2wt | wt | wt | wt | wt | m1m2wt |
| ANUBL1 | wt | wt | wt | wt | wt | wt | wt | wt | wt | m1wt | wt | m1wt | wt |
| EPRS | wt | wt | wt | wt | wt | wt | wt | wt | wt | wt | wt | wt | wt |
| TTC3 | wt | wt | wt | m1wt | m1wt | wt | m1wt | m1wt | wt | m1wt | wt | ND | ND |
| SFRS12IP1 | wt | wt | wt | wt | wt | m1 | m2m1wt | wt | m1 | m1 | wt | m1wt | m1wt |
| ZFR | wt | wt | wt | wt | wt | wt | wt | m1wt | m1wt | m1wt | wt | wt | m1wt |
| DAB2IP | wt | wt | wt | m1wt | m1wt | wt | m1p1 | m1wt | m1p1 | wtp1 | wt | wt | wt |
| PHACTR4 | wt | wt | wt | m1wt | m1wt | m1wt | m2 | m1wt | ND | wtp1 | wt | m1wt | wt |
| MLL3 | wt | wt | wt | m1wt | m1wt | wt | m1wt | m1wt | m1wt | m1wt | wt | m1wt | m1wt |
| PDS5A | wt | wt | wt | wt | wt | wt | wt | wt | wt | wt | wt | wt | wt |
| TROVE2 | wt | wt | wt | m1wt | wt | wt | m1wt | wt | m1wt | wt | wt | wt | m1wt |
| USP42 | wt | wt | wt | wt | wt | wt | m1wt | wt | m1wt | m1wt | wt | wt | m1wt |

|  | **Endometrial Cancer** | | | | | | | **Ovarian Cancer** | | | | |
| --- | --- | --- | --- | --- | --- | --- | --- | --- | --- | --- | --- | --- |
| **Gene** | **ECC-1** | **SCRC** | **KLE** | **HEC59** | **AN3CA** | **HEC1B** | **RL95-2** | **CAOV-3** | **SKOV-6** | **SKOV3** | **OVCAR3** | **2774** |
| PRRT2 | wt | wt | wt | m1wt | wtp1p2 | m1wt | m1wt | wt | wt | wt | wt | m1wtp1 |
| TSHZ2 | wt | wt | wt | m1wt | m1wt | m1wt | ND | wt | wt | m1wt | m1wt | m1wt |
| ANLN | wt | wt | wt | wt | wt | wt | wt | wt | wt | wt | wt | wt |
| CEP164 | wt | wt | wt | wt | m1wt | m1wt | m1 | wt | wt | m1wt | wt | m1 |
| CNOT1 | wt | wt | wt | m1m2 | m1wt | m1wt | m1m2m3 | wt | wt | wt | wt | m1wt |
| KCNMA1 | wt | wt | wt | wt | wt | wt | m1m2wt | wt | wt | wt | wt | m1m2wt |
| ANUBL1 | wt | wt | wt | wt | wt | wt | wt | wt | wt | wt | wt | wt |
| EPRS | wt | wt | wt | wt | wt | wt | wt | wt | wt | wt | wt | wt |
| TTC3 | ND | ND | wt | ND | m1wt | wt | wt | wt | wt | wt | wt | ND |
| SFRS12IP1 | wt | wt | wt | m1wt | wt | wt | wtp1 | wt | wt | wt | wt | m1 |
| ZFR | wt | wt | wt | m1wt | m1wt | m1wt | wt | wt | wt | wt | wt | m1wt |
| DAB2IP | wt | wt | wt | m1wt | wt | wt | wt | wt | wt | wt | wt | wt |
| PHACTR4 | wt | wt | wt | m1wt | m1wt | wt | m1wt | wt | wt | wt | wt | m1wt |
| MLL3 | wt | wt | wt | wt | m1wt | m1wt | m1wt | wt | wt | wt | wt | m1m2 |
| PDS5A | wt | wt | wt | wt | m1wt | wt | wt | wt | wt | wt | wt | wt |
| TROVE2 | wt | wt | wt | m1 | m1wt | m1wt | m1wt | wt | wt | m1wt | wt | wt |
| USP42 | wt | wt | wt | m1wt | m1wt | wt | m1wt | wt | wt | wt | wt | wt |
